# Large Thermo- and Mechanosalient Actuation via Cooperative Twist Elasticity-Induced Packing Motif Conversion

**DOI:** 10.1021/jacs.6c05413

**Published:** 2026-07-01

**Authors:** Kyoungtae Hwang, Indranil Bhattacharjee, Sooyeon Ra, Jin Hyeok Jang, Jinwoo Park, Haseong Kim, Minwoo Jang, Min Wook Lee, Dong Ryeol Whang, Dohyun Moon, Johannes Gierschner, Hyungbum Park, Sang Kyu Park

**Affiliations:** † Institute of Advanced Composites Materials, 58975 Korea Institute of Science and Technology (KIST), Wanju, Jeonbuk 55324, Republic of Korea; ‡ Madrid Institute for Advanced Studies, IMDEA Nanoscience, Ciudad Universitaria de Cantoblanco, C/Faraday 9, 28049 Madrid, Spain; § Department of Mechanical Engineering, 34958 Incheon National University, Incheon 22012, Republic of Korea; ∥ Department of Electronic Materials Engineering, 34955 Hoseo University, Asan 31499, Republic of Korea; ⊥ Beamline Department, 92226 Pohang Accelerator Laboratory (PAL)/Pohang University of Science and Technology (POSTECH), Pohang 37673, Republic of Korea

## Abstract

Dynamic molecular crystals capable of undergoing cooperative structural transformations offer exciting prospects for next-generation actuators, sensors, and stimuli-responsive materials. However, realizing large-scale deformation in the solid state—particularly through torsional mechanisms—remains rare. Here, we examine a cyanostilbene derivative, αDDDCS, that exhibits solid-state twist elasticity enabled by cooperative conformational torsion and packing motif conversion. This system features three enantiotropic polymorphs that interconvert through thermoelastic and mechanosalient phase transitions, reflecting a competition between thermodynamic stability and kinetic accessibility, including a Y → C thermoelastic transformation involving a 27% lattice elongation—among the largest reported to date. Single-crystal X-ray diffraction, variable-temperature characterization, and quantum chemical calculations reveal that the transformation proceeds through a π-stacking-to-μ-herringbone transition, governed by a distinct torsional barrier and polymorph-specific free energy landscape. Remarkably, this is the first demonstration of twist elasticity accessed via mechanosalient actuation. Our findings establish conformational twist as a viable molecular design element for achieving high-strain responsiveness in dynamic crystals.

## Graphical abstract

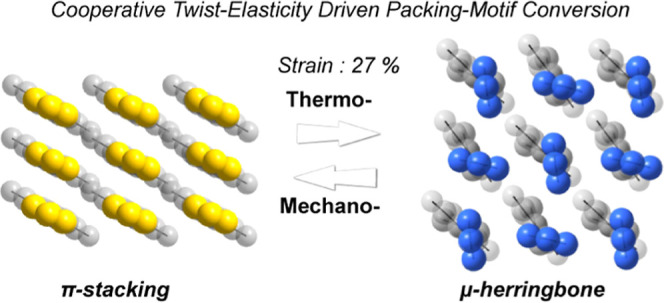

## Introduction

Dynamic crystals represent an emerging class of solids that undergo structural reorganization coupled to mechanical deformation under thermal, optical, or mechanical stimuli.^1,2^ Within this broader family, molecular martensites constitute a distinctive subset that displays martensitic-like transitions arising from cooperative molecular rearrangements.^3,4^ These systems can translate angstrom-scale molecular motions into pronounced macroscopic actuation, providing an attractive platform for soft robotics, adaptive devices, and stimulus-responsive actuators. However, achieving larger deformations (strain, ε = Δ*L*/*L*_0_ × 100, where *L*_0_ is the reference length) above 20% remains a critical challenge for practical implementation. To address this challenge, the present study aims to develop a new class of thermoelastic molecular martensites—materials capable of thermally induced martensitic transitions—that exhibit large mechanical deformation and to elucidate the molecular-level mechanisms governing this behavior.

To date, numerous thermoelastic molecular crystals have been reported. Owing to their nonsphericity and intrinsic flexibility, molecular systems undergo martensitic transitions through combinations of translational, rotational, and conformational motions, thus, far more complex than purely displacive transitions in atomic crystals.^4^ Most known examples exhibit only modest (<10%) deformation because their transformations conserve the overall packing motif (Figure 1 and Table S1). The most common mechanism is pitch-angle modulation (“molecular sliding”), observed across diverse packing motifs (crystals 1–14).^5–18^ Comparable strain levels also occur in systems dominated by molecular rotation (crystals 15–19)^19–23^ or by conformational changes (crystals 20–25).^24–29^ Despite differing mechanistic modes of action, these transitions invariably preserve the parent packing motif, which inherently limits the achievable macroscopic deformation.

**Figure 1:**
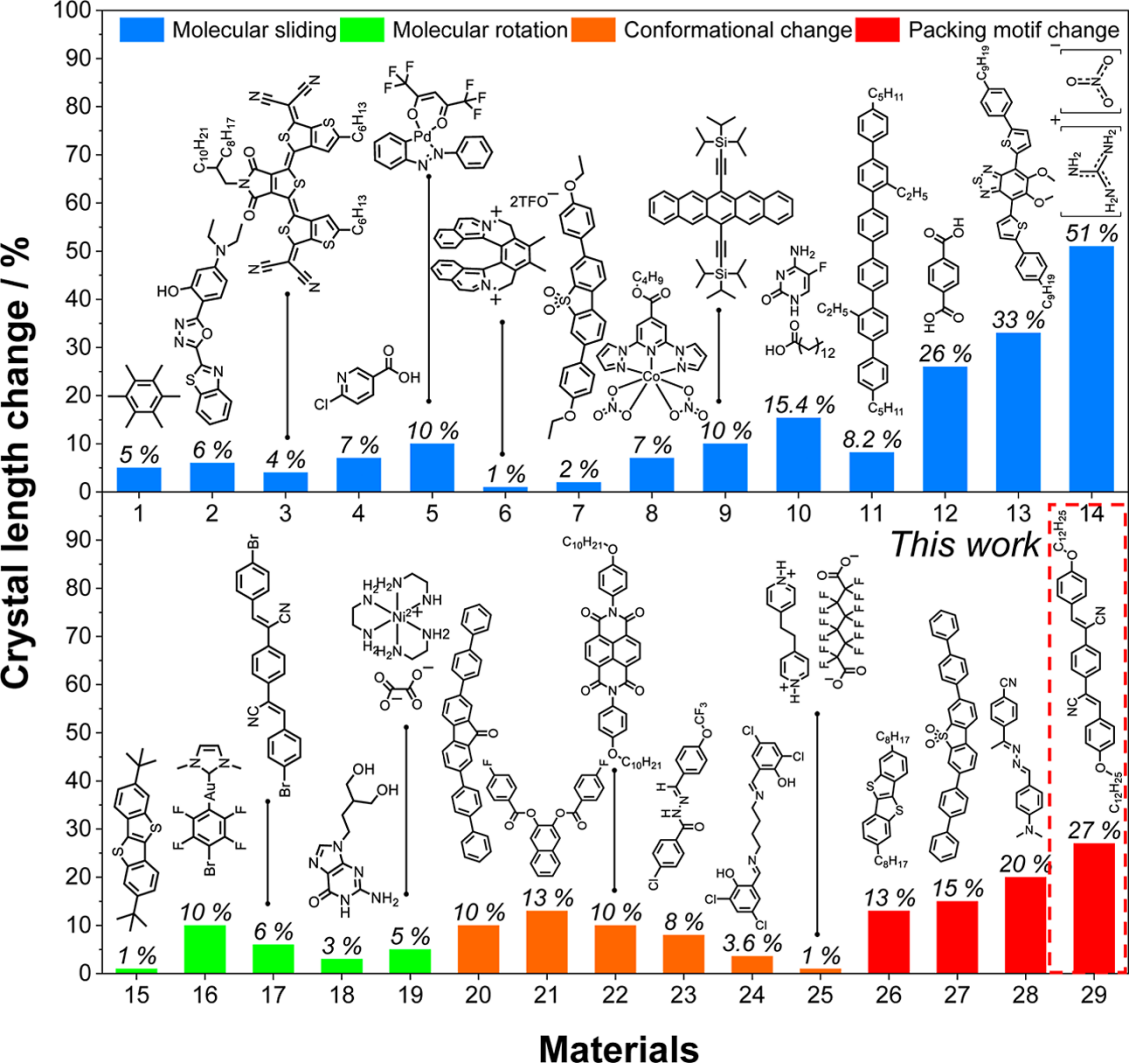
Mechanistic classification of thermosalient molecular crystals. Crystals (1–29) are grouped by a dominant structural transformation pathway. The reported crystal elongation (Δ*L/L*_0_ × 100, %) is summarized alongside the corresponding molecular structures. References are provided in the Introduction.

These observations suggest that preserving the original packing motif during the transition may inherently limit the achievable thermoelastic strain, although crystals 12–14 in Figure 1 and Table S1, which exhibit significant changes in pitch angle, have demonstrated exceptional deformations exceeding 20%. A promising strategy to overcome this limitation is to design phase transitions that involve changes in the packing motif itself. For example, a transition from dense π-stacking, where planar molecules are aligned in parallel with short intermolecular separations, to more open herringbone structures, where molecular planes intersect at a defined angle, can lead to substantial volume changes. Rare examples supporting this approach include 2,7-dioctylbenzothieno­[3,2-*b*]­benzothiophene and 4-dimethylaminobenzaldehyde­(4-cyanophenyl-ethylidene)­hydrazone (E,E) crystals (26 and 28 in Table S1), both of which undergo transitions from herringbone to π-stacked structures, exhibiting approximately 13% and 20% deformations, respectively, as well as a 3,7-di­([1,1′-biphenyl]-4-yl)­dibenzo­[*b*,*d*]­thiophene 5,5-dioxide crystal (27 in Table S1), which undergoes a transformation from a partially herringbone to a fully herringbone configuration through torsional reorganization, resulting in a deformation of approximately 15%.^30–32^ These cases highlight the potential of cooperative packing motif conversion as an effective mechanism for enhancing thermoelastic strain.

Based on these insights, we recognized cyanostilbene derivatives—a long-term subject of our studies—as promising candidates for achieving large thermoelastic deformation through torsion-driven packing motif transformation. The concept of “twist elasticity” was introduced to describe the ability of cyanostilbene molecules to undergo significant torsional or conformational changes during self-assembly in response to external stimuli.^33,34^ Contorted cyanostilbene molecules adjust their torsional coordinates to facilitate the formation of strong intermolecular dipolar interaction networks, such as –CN···HC–, during crystallization. This behavior is enabled by their flexible π-conjugated backbone, which can accommodate steric congestion from the cyano-substituents through adaptive opening of the vinyl C–C=C angles, thereby allowing even sterically constrained backbones to undergo planarization. These structural features not only support the formation of diverse packing motifs—such as μ-herringbone, or 1D,^35,36^ 2D,^37^ and X (crossed)^36^ π-stacking—but also give rise to pronounced solid-state luminescence enhancement through suppression of large-amplitude motion in the rigid crystal environment.^35,38–40^ Despite its structural adaptability and photophysical advantages, twist elasticity has not yet been exploited as a driving force for cooperative martensitic transitions, nor has it been reported in any known solid-state phase transitions. If successfully implemented in the solid state, twist elasticity could offer a promising opportunity to advance the design of highly deformable dynamic crystals.

Here, we report a rare example of cooperative, thermoelastic solid-state twist elasticity involving conformational torsion and packing motif conversion, realized in a cyanostilbene-based molecular martensite, (2Z,2′Z)-2,2′-(1,4-phenylene)­bis­(3-(4-(dodecyloxy)­phenyl) acrylonitrile) (αDDDCS). While αDDDCS was introduced earlier,^39^ its crystal structures remained unreported, limiting molecular-level understanding of its transformation behaviors. In this work, we show that αDDDCS exhibits three enantiotropic polymorphs (B-, Y-, and C-phases) that interconvert through thermoelastic and mechanosalient transitions (i.e., mechanically triggered cooperative transitions, accompanied by sudden shape changes), reflecting a competition between thermodynamic stability and kinetic accessibility.^1^ Among them, the Y → C transformation features an exceptional lattice elongation of 27%, placing it among the largest deformations reported to date in dynamic molecular crystals. Through single-crystal X-ray diffraction (SCXRD), variable-temperature analyses, and computational modeling, we reveal that this transformation proceeds via a highly cooperative conformational twist coupled with a μ-herringbone-to-π-stacking packing conversion. The B → Y and C → Y transitions are further distinguished by their dual mechanochromic and mechanosalient character, most probably governed by a distinct conformational energy barrier and polymorph-dependent thermodynamic stability. Notably, this represents the first mechanosalient realization of solid-state twist elasticity, establishing a new mechanism for accessing high-strain actuation in molecular materials.

## Results and Discussion

### Section 1: Transition Pathway

αDDDCS (Figure 2a) was selected as a model compound, as its pronounced optical response (Figure S2)—most notably a large emission shift (Δ*E*_em_ ∼ 0.39 eV)—strongly suggests substantial structural reorganization in the solid state.^39^ Variable-temperature analyses reveal three enantiotropic polymorphs (Y-, B-, and C-phases) that differ not merely in optical properties but, crucially, in molecular conformation and packing topology (Figure 2b,c). The low-temperature yellow-emissive phase (Y-phase) adopts a nearly planar backbone arranged in a π-stacked motif, whereas the low-temperature blue-emissive phase (B-phase) features a twisted backbone organized in a μ-herringbone (μ-HB) packing, where individual phenyl-rings interact locally in an edge-to-face fashion;^35,41^ for details, see Section 2. Upon heating, both phases converge to a common high-temperature cyan-emissive phase (C-phase), with properties that are strongly consistent with a μ-HB arrangement. Importantly, the Y → C transition entails a packing-topology conversion (π-stack → μ-HB) and produces a large thermoelastic strain of 27%, whereas the B → C transition occurs within a conserved μ-HB framework and results in only minimal strain (1.2%). This contrast enables a direct comparison between motif-converting and motif-preserving martensitic pathways within a single molecular system.

**Figure 2:**
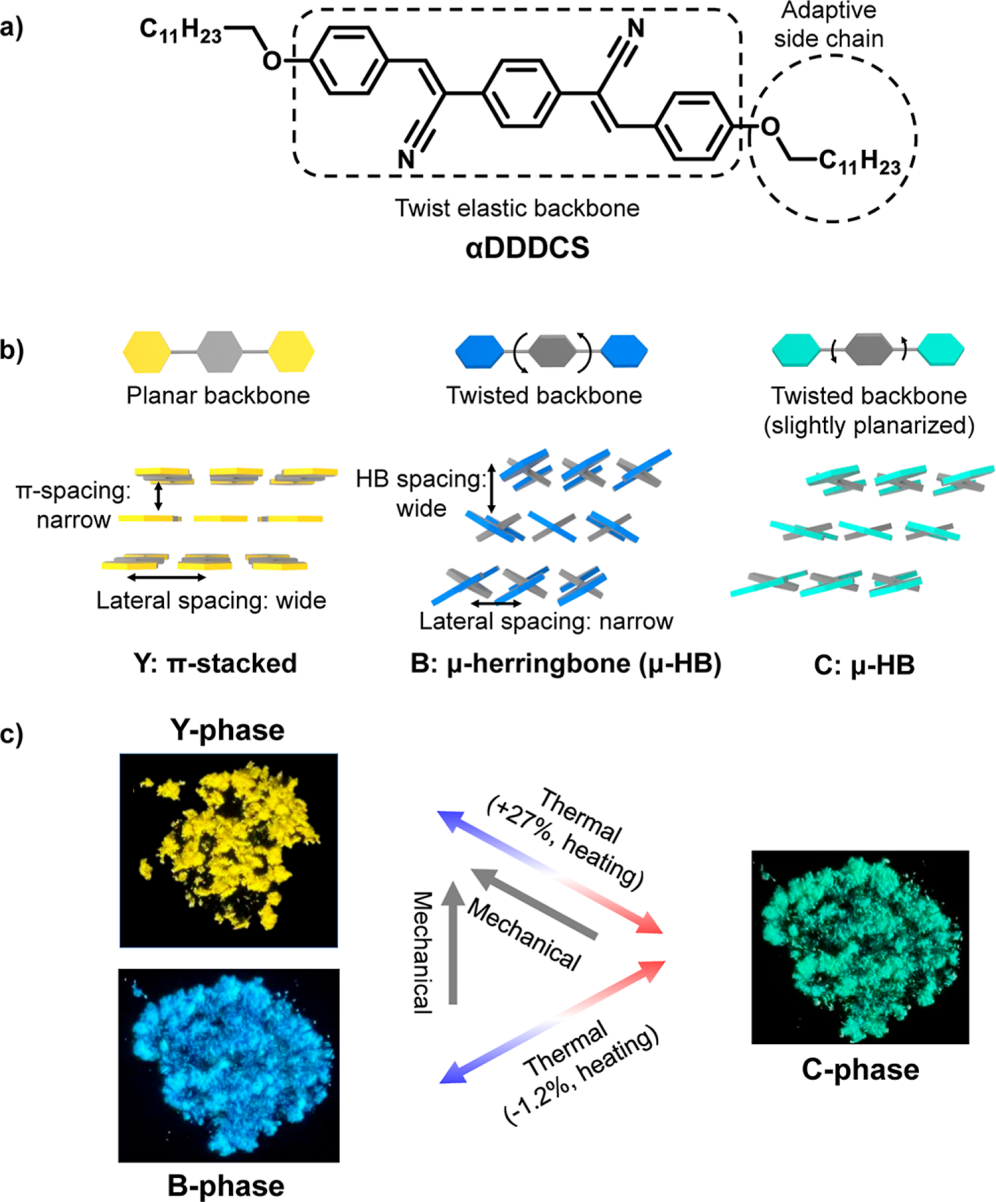
Outline of the chemical structure, polymorphs, and phase transition of αDDDCS. (a) Chemical structure of αDDDCS, consisting of a twist-elastic π-conjugated backbone and adaptive alkoxy side chains. Here, α denotes cyano substitution at the olefinic carbon adjacent to the inner phenyl ring. (b) Schematic conformations and packing motifs of the three enantiotropic polymorphs: the yellow-emissive phase (Y-phase) adopts a nearly planar backbone with a π-stacked packing, the blue-emissive phase (B-phase) features a twisted backbone arranged in a μ-herringbone (μ-HB) motif, and the cyan-emissive phase (C-phase) consists of a slightly planarized twisted backbone in a μ-HB motif. (c) Fluorescence images of Y-, B-, and C-phase crystals and a schematic phase map summarizing their interconversion: Upon heating, the Y- and B-phases transform into the high-temperature C-phase with large (+27%) and small (−1.2%) thermoelastic strain, respectively. Notably, mechanical input can trigger a B/C → Y conversion.

The transition behavior of the Y- and B-phases was first examined by differential scanning calorimetry (DSC) combined with variable-temperature fluorescence microscopy (VT-FM) (Figure 3a–d and S3; Movies S1 and S2). The as-prepared Y-phase exhibits a single first-order transition to the C-phase at 94.8 °C (T_Y → C_, point (iii)), accompanied by pronounced crystal morphing. Upon cooling, however, the C-phase shows only limited conversion back to the Y-phase (T_C → Y_, point (iv)) and instead transforms predominantly into the B-phase (T_C → B_, point (v)). Thus, after the first thermal cycle, an initially Y-phase sample is largely converted into the B-phase, and subsequent cycles follow a B-phase-dominated pathway.

**Figure 3:**
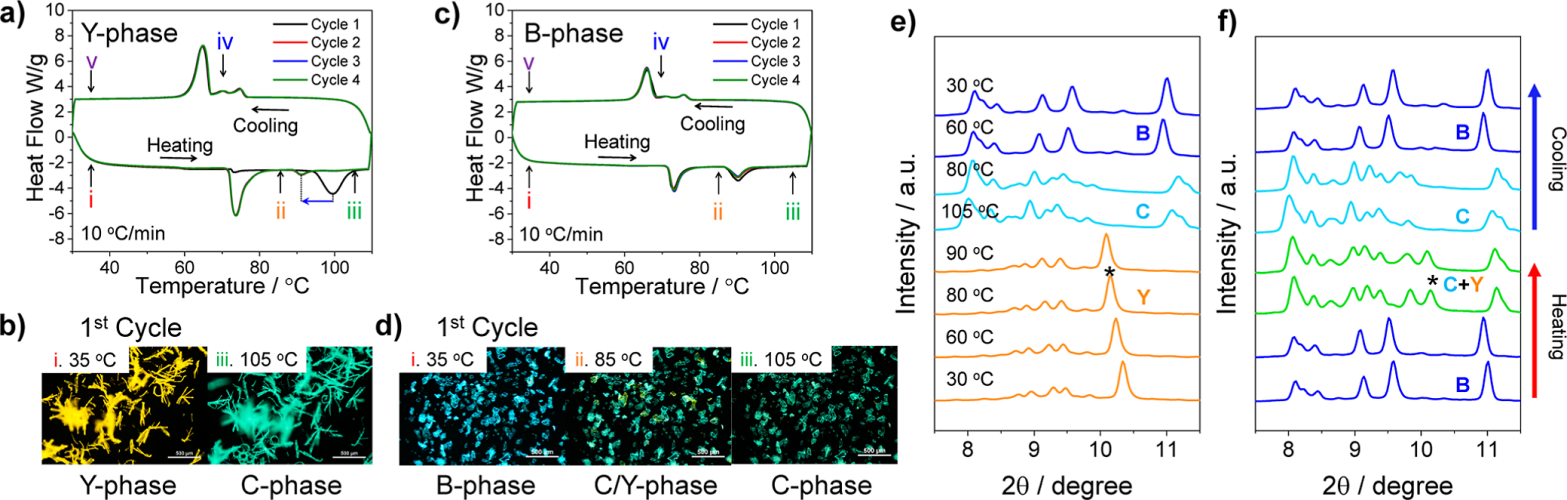
Thermally driven phase transitions among the three polymorphs of αDDDCS. (a) DSC traces of Y-phase crystalline powders showing a first-order transition upon heating (T_Y → C_: 94.8 °C) and cooling (T_C → B_: 66.7 °C). (b) VT-FM images capturing pronounced deformation during the Y → C transition. (c) DSC traces of the B-phase crystalline powders showing two first-order transitions upon heating (T_B → C_: 71.5 °C, T_Y → C_: 87.5 °C). (d) VT-FM images capturing minimal deformation and transient C–Y coexistence during the B → C transition. (e,f) Variable-temperature powder X-ray diffraction (VT-PXRD) patterns of the B- and Y-phase crystalline powders, respectively.

The B-phase shows a more complex first heating cycle, with two endothermic events. The lower-temperature transition (71.5 °C, point (ii)) is assigned primarily to the B → C transformation. In parallel, a minor fraction of the B-phase undergoes mechanically triggered B → Y conversion under local constraint, such as adhesion to the substrate or neighboring crystals (Movie S2 and Section 5). This transiently generated Y-phase subsequently transforms into the C-phase upon further heating, giving rise to the second endothermic event (87.5 °C, point (iii). After reaching the C-phase, the cooling pathway again favors C → B conversion, with only minor C → Y conversion.

Meanwhile, two minor features in the DSC traces should be noted. The weak endothermic feature near 70 °C during the first heating cycle of the Y-phase is attributed to trace B-phase impurity formed under the Y-phase growth conditions, as supported by fluorescence microscopy and its agreement with the intrinsic B → C transition temperature (Figure 3a,c and S4; Supplementary Note 1). In addition, the marked shift in the Y → C transition temperature between the first and subsequent heating cycles (Figure 3a, blue arrow) is attributed to differences in prior thermal and mechanical history rather than to an additional polymorphic phase,^16^ as further supported by VT-PXRD analysis below and discussed in Supplementary Note 1.

VT-PXRD analysis further corroborates the transition pathways and confirms the structural identity of the Y-phase generated through different routes (Figure 3e,f). Upon heating, diffraction patterns of both the Y- and B-phases evolve into an identical set of reflections corresponding to the high-temperature C-phase, whereas subsequent cooling predominantly regenerates the B-phase pattern regardless of the starting phase, consistent with the DSC and VT-FM observations. Notably, at 80 °C, a characteristic Y-phase reflection (marked with an asterisk) appears at the same 2θ position in both the mechanically generated Y-phase produced during B-phase heating (Figure 3f) and the directly crystallized Y-phase (Figure 3e), indicating that they share the same crystal structure. This assignment is further supported by detailed analysis of the lamellar reflection region (Figure S5). Specifically, the mixed Y/C-phase pattern of the B-origin sample at 80 °C during heating is well reproduced by the sum of two reference patterns: the directly crystallized Y-phase collected at 80 °C during heating and the C-phase collected from the B-origin sample at 80 °C during cooling. No additional reflections are required to account for the mixed-phase pattern, thereby excluding the involvement of another polymorph.

To elucidate the martensitic nature and mechanical consequences of the phase transitions, variable-temperature cross-polarized optical microscopy (VT-CPOM) was performed on single crystals. Both Y → C and B → C transformations occur abruptly in a single-crystal-to-single-crystal fashion (Movies S3,S4,S5,S6), consistent with diffusionless martensitic behavior. Under mechanical constraint introduced by a thin adhesive layer between the crystal and the substrate, the transitions slowed sufficiently to enable clear visualization of phase-front propagation (Movies S7 and S8), further substantiating their martensitic character. Notably, despite their similar mechanistic signatures, the macroscopic responses differ markedly: the Y → C transition produces a substantial length change of approximately 27%, whereas the B → C transition results in only minimal deformation (∼1%). The large Y → C deformation is accompanied by slight striation and microcrack formation during the initial transformation, but five-cycle VT-CPOM measurements show that subsequent B ↔ C cycling proceeds without further noticeable damage accumulation; crystals initially prepared in the B-phase undergo repeated B ↔ C transformations without observable defect formation (Figure S6). Consistent with this retained mechanical integrity, supplementary mechanical-output measurements show that the Y → C transition exhibits substantial force-generating capability, with a force density of 3.6 ± 3.0 × 10^8^ N·m^–3^. Glass-plate pushing experiments further indicate that the threshold for stable force generation without noticeable bending or catastrophic failure lies between 1.6 and 5.8 × 10^8^ N·m^–3^, while bead-displacement experiments demonstrate measurable mechanical work output, yielding a conservative work density estimate of 63–168 J·m^–3^, likely underestimated by bending dissipation in the thin, high-aspect-ratio crystals (Figures S7–S9, Tables S3–S5, Movies S9,S10,S11, Supplementary Note 2).^24^ Within αDDDCS, this direct comparison reveals a striking contrast between motif-converting and motif-preserving martensitic pathways, with only the former associated with substantial thermoelastic strain—a disparity whose structural basis is clarified by SCXRD in the following section.

### Section 2: Structural Analysis

To define the structural basis of the contrasting thermoelastic responses identified in Section 1, we determined the room-temperature crystal structures of the Y- and B-phases of αDDDCS by synchrotron SCXRD. The extreme thinness of the crystals had previously precluded structural analysis, but synchrotron measurements enabled reliable structural refinement of both polymorphs.

The B-phase adopts a severely twisted molecular geometry (Figure 4a), characterized by large backbone torsion angles (θ_i_/θ_o_ for the inner/outer single bonds of the cyanostilbene core; −152.4(6)°/–145.7(6)°). Both alkoxy side chains adopt the anti conformation, and the molecule possesses inversion symmetry. This pronounced molecular distortion disfavors efficient face-to-face π-overlap and instead stabilizes a μ-HB packing motif (Figure 4b–d).^41^ In this arrangement, molecules stand nearly upright with minimal long-axis displacement (x-slip, Δ*x* = 0.56 Å), while substantial sliding occurs along the molecular short axis (y-slip, Δ*y* = 3.37 Å). The packing is reinforced by C–N···H–C dipolar interactions that extend along the crystallographic *c*-axis to form molecular sheets (Figure 4d), while π-interactions occur predominantly in an edge-to-face manner. The resulting μ-HB topology thus emerges directly from the contorted backbone geometry and symmetric molecular arrangement.

**Figure 4:**
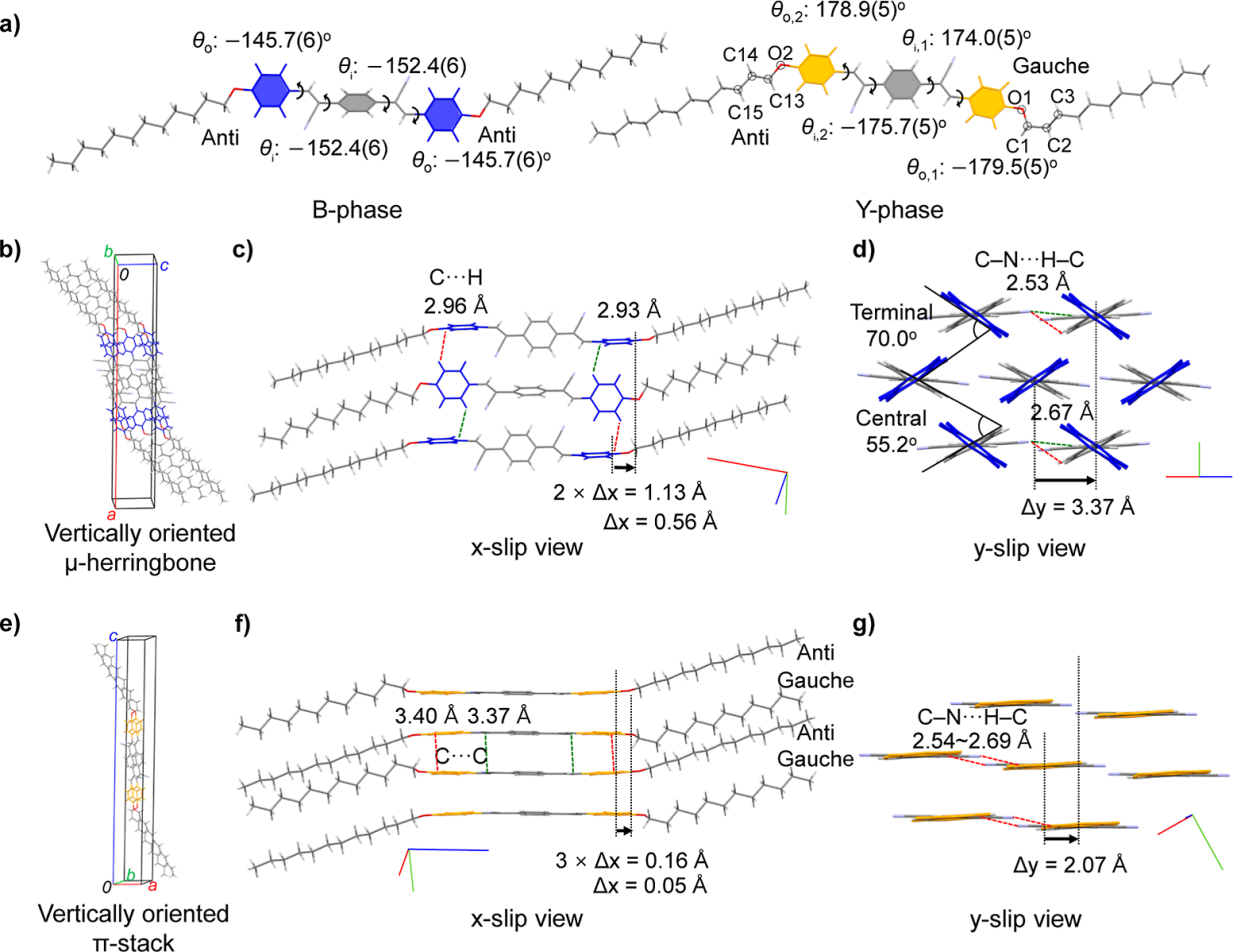
Molecular and packing structures of B- and Y-phase crystals. (a) Twisted geometry in the B-phase (θ_i_ = −152.4(6)°, θ_o_ = −145.7(6)°) and nearly planar geometry in the Y-phase (θ_o,1_ = −179.5(5)°, θ_i,1_ = 174.0(5)°, θ_i,2_ = −175.7(5)°, θ_o,2_ = 178.9(5)°). (b–d) μ-Herringbone (μ-HB) packing motif of the B-phase: (b) vertically oriented μ-HB arrangement, (c) edge-to-face contacts (C···H, 2.93 and 2.96 Å) in the x-slip (Δ*x* = 0.56 Å) view, and (d) herringbone angles in the y-slip (Δ*y* = 3.37 Å) view (central ring: 55.2°, terminal ring: 70.0°) with C–N···H–C interactions (2.53, 2.67 Å). (e–g) π-Stack packing motif of the Y-phase: (e) vertically oriented π-stack arrangement, (f) short π-stacking distance (3.37–3.40 Å) with minimal x-slip (Δ*x* = 0.05 Å), and (g) C–N···H–C interactions along [−210] with a y-slip (Δ*y* = 2.07 Å). Intermolecular packing parameters of the B- and Y-phases (e.g., *x*- and *y*-slips) are illustrated and summarized in detail in Figure S12 and Table 1

In contrast, the Y-phase adopts an almost planar molecular conformation (|θ| < ∼6°) (Figure 4a). The two alkoxy chains adopt distinct gauche and anti conformations, breaking inversion symmetry and introducing subtle conformational asymmetry. The near-planar backbone enables slipped π-stacking, characterized by minimal x-slip (Δ*x* = 0.05 Å; Figure 4f) and moderate y-slip (Δ*y* = 2.07 Å; Figure 4g), producing short π-contact distances (3.37–3.40 Å) indicative of strong π-electronic coupling. Although C–N···H–C dipolar contacts are retained between laterally paired molecules along the [−210] direction (Figure 4g), the overall packing arrangement differs fundamentally from that of the B-phase. These structural differences are also accompanied by distinct mechanical responses, as supported by nanoindentation measurements (Figure S10, Supplementary Note 3).

Taken together, the Y- and B-phases differ not only in torsional geometry but also in packing topology—slipped π-stacking versus μ-HB. Although full structural refinement of the C-phase by SCXRD was not achieved because of progressive diffraction deterioration, thermally induced sagging of the extremely thin crystals, and sample–mount interactions near the phase transition temperature, complementary structural information was obtained from VT-PXRD measurements collected at 378 K (Figure S11). Indexing and Pawley-type refinement yielded unit cell parameters closely related to those of the B-phase, as summarized in Table 1. Considering the axis transformation *b*_B_ → *c*_C_ and *c*_B_ → *a*_C_, the PXRD-derived C-phase lattice parameters correspond to changes of approximately −2.0% and +0.1%, respectively, relative to the B-phase. These values are in good agreement with the minimal macroscopic deformation (∼1%) observed during the B → C transition at the crystal level (Movie S5). Such small lattice changes suggest that the B → C transition largely preserves the μ-HB-type packing topology of the B-phase, rather than involving a major redefinition of molecular packing. Together with the photophysical analysis presented in Section 3, these results support a C-phase structure that remains closely related to the B-phase. In contrast, the Y → C transformation requires collective changes in both molecular conformation and intermolecular registry, providing a structural rationale for the markedly different thermoelastic strains observed for the two pathways.

**Table 1:** Crystallographic and Intermolecular Arrangement Parameters for the B-, Y-, and C-Phases

	B-phase	Y-phase	C-phase^e^
Z	2	2	
V/Å^3^	2078.4	2118.8	2256.2
ρ/g cm^–3^ ^a^	1.120	1.099	
a/Å	43.06	5.76	6.74
b/Å	7.17	7.63	47.65
c/Å	6.73	48.27	7.03
α	90.0	89.2	90.0
β	90.5	87.3	90.7
γ	90.0	89.1	90.0
length			
C_α_C_ω_/Å^b^	15.99	16.13	
Slips^c^			
Δ*z*/Å	3.31	3.33	
Δxy/Å	3.41	2.07	
Δ*x*/Å^d^	0.56 (3.5%)	0.05 (0.3%)	
Δ*y*/Å	3.37	2.07	
center-to-center			
1st neighbor	4.92	3.80	
2nd neighbor	4.92	5.76	
angle	86.4°	88.2°	

a ρ = Z·M·V^–1^·N_A_^–1^, where M = 701.01 g/mol, N_A_ = 6.022 × 10^23^ mol^–1^.

b Distance between the terminal C-atoms of the DSB core.

c Average slips from translationally equivalent molecules (i, i+2)/2 [Δ*z* is perpendicular to the backbone plane, and Δ*x* is along the long molecular axis].

d [% of the molecular backbone length C_α_C_ω_].

e Unit cell parameters were derived from VT-PXRD indexing and Pawley refinement at 378 K (B-phase origin); full structural refinement of the C-phase was not feasible.

Structural parameters for the B- and Y-phases were obtained from synchrotron single-crystal X-ray diffraction analysis, whereas the C-phase parameters were derived from VT-PXRD indexing and Pawley refinement performed at 378 K.

### Section 3: Photophysical Analysis

The SCXRD results provide a structural basis for understanding the staggering fluorescence shift observed among the αDDDCS polymorphs, and, in turn, offer important clues for inferring the structure of the unresolved C-phase. Figure 5 shows the UV–vis absorption and photoluminescence (PL) spectra; key photophysical parameters are summarized in Table 2, including absolute PL quantum yield (Φ_F_) and decay traces of each phase; details are given in Figures S13, S14 of the Supporting Information. PL spectra and lifetimes were obtained directly from single crystals, while the absorption spectra were measured using nanocrystal (NC) suspensions to circumvent the high optical density of the single crystals.^35^ It is noted that the PL spectra of the NC suspensions resemble those of the single crystals (see Figure S15), so that the NC absorption can be correlated to the crystal structure analysis.^35^

**Figure 5:**
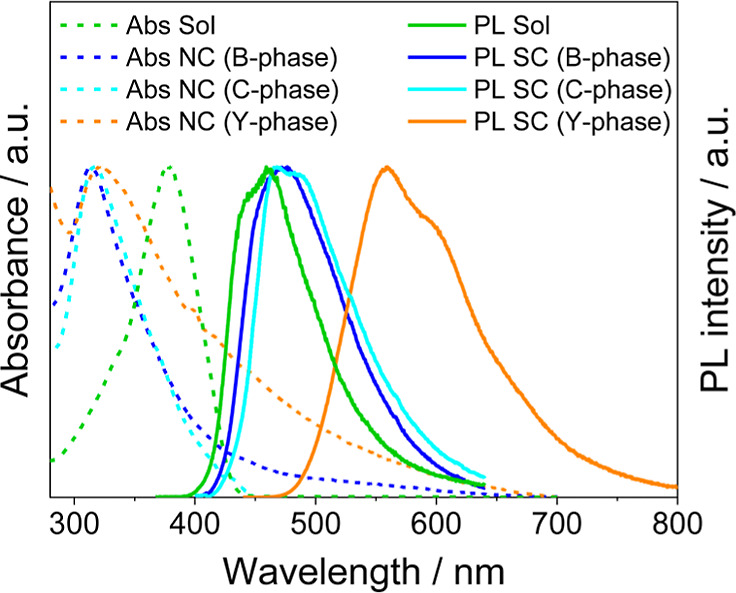
UV–vis absorption (dotted lines) and photoluminescence spectra (solid lines) of the B-, Y-, and C-phases (λ_ex_ = 365 nm (for B and C), 420 nm (for Y)).

**Table 2:** Photophysical Properties of αDDDCS in Solution (THF, c = 2 × 10^–5^ mol·L^–1^), Nanocrystal Suspension (NC, in H_2_O), and Single Crystals (B-, C-, and Y-Phases)

	solution		NC	single crystal					
	*E*_abs_ [eV] (λ_abs_ [nm])	*E*_em_ [eV] (λ_em_ [nm])	*E*_abs_ [eV] (λ_abs_ [nm])	*E*_em_ [eV] (λ_em_ [nm])	ΔE_cryst, em_ [eV]^a^	τ_avg_ [ns]^b^	Φ_F_	*k*_r_ [10^7^ s^–1^]^c^	*k*_nr_ [10^7^ s^–1^]^c^
B	3.26 (380)	2.81 (441)^42^	3.96 (313)	2.61 (475)	–0.20	6.44	0.91	14	1.4
C (85 °C)			3.91 (317)	2.65 (468), 2.57 (482)	–0.16	3.87	-	-	-
Y			3.88 (320)	2.22 (558), 2.10 (591)	–0.59	21.32	0.46	2.2	2.5

a ΔE_cryst, em_ = E_vert, em_ (crystal) – E_vert, em_ (solution).

b Intensity-averaged lifetimes were obtained from biexponential decay times through τ_avg_ = ∑A_i_τ_i_^2^/∑A_i_τ_i_; detection wavelengths were 465 nm (B,C) and 555 nm (Y).

c Radiative (*k*_r_) and nonradiative rates (*k*_nr_) were calculated via *k*_r_ = Φ_F /_ τ_r_, τ_r_ = (*k*_r_ + *k*_nr_)^−1^.

Both B- and Y-phases exhibit hypsochromic shifts in the NC absorption maxima (*E*_max.A_) relative to solution, indicating H-aggregation.^35^ This is consistent with the “side-by-side” packing of the long molecular axes of the nearest-neighbor pairs with minimal x-slip (*vide supra*).^35^ While the absorption maxima are quite similar for the three phases, the PL spectral position of Y (*E*_max,F_ = 2.22 eV, λ_em_ = 558 nm) shows a very considerable bathochromic shift in comparison to the B-phase (*E*_max,F_ = 2.61 eV, λ_em_ = 475 nm), corresponding to a polymorph shift of ΔE_poly_ = −0.39 eV. The C-phase emission peaks at 2.57 eV (482 nm), thus rather similar to B. Although similarly large Δ*E* were observed in polymorphs of DCS-based materials,^34,38,43–45^ the underlying reasons have not been explicitly revealed by now, essentially due to lack of detailed structural information. In fact, spectral shifts are a complex matter,^35^ with excitonic, geometrical, polarizability,^46^ and possible excimeric contributions.^47–49^ In the current case, the anisotropic polarizability shift is expected to be very similar across the different polymorphs due to the predominant side-by-side arrangement of neighbor molecules in all cases, while the excimer shift should be rather small, as strong excimers exhibit very broad, unstructured Gaussian-shaped features,^47,48^ which are not observed for αDDDCS.

Since full structural information is available for the B- and Y-phases, excitonic and geometrical contributions can be disentangled through single-point TD-DFT calculations (Figures S16–S19).^34,36,38,50^ For this, the crystal shift ΔE_cryst_ is estimated from the lowest excited state (S_1_) of small (tetramer) clusters in comparison to the fully relaxed monomer (for details of the procedure, see Supporting Information).

This screening method allows for quite reliable screening, particularly for different polymorphs of the same compound.^34,38^ In the current case, a bathochromic shift of ΔE_poly_ = −0.48 eV for B_T_ → Y_T_ (Table 3) is observed, in qualitative agreement with the experiment. In order to disentangle intra- and intermolecular contributions, we first compare E_vert_ of the fully relaxed monomer (F_M_) with those of the partially relaxed monomers adapting the geometries found in the crystals (B_M_ and Y_M_, respectively). As summarized in Table 3, E_vert_ of Y_M_ is found at 0.13 eV below that of F_M_ due to the planarization in the Y-phase, while B_M_ is found at 0.14 eV above F_M_, due to strong twisting in the B-phase (see Figure 4). Thus, the monomeric shift from B_M_ → Y_M_ amounts to −0.27 eV and therefore already accounts for more than half of the overall calculated crystal shift. In a second step, we placed Y_M_ in the tetramer arrangement of B-phase, i.e., B_T_(Y_M_); this gave almost the same S_1_ energy as in Y_T_. This finding indicates that, even within the same packing arrangement (B_T_), excitonic interactions are governed not only by the molecular geometry itself but also by geometric change-induced modifications of the intermolecular interactions. This can be rationalized by the increase in the oscillator strength (*f*_1_) upon planarization, i.e., 1.79 for B_M_ to 2.01 for Y_M_.

**Table 3:** TD-DFT Calculations (CAM-B3LYP) for Monomer and Tetramer

	geometry	name		E_vert_	*f*	ΔE_ES_	ΔE_cryst_
monomer	fully relaxed	F_M_	S_1_	3.50 eV	1.95		
	B	B_M_	S_1_	3.64 eV	1.79		
	Y	Y_M_	S_1_	3.37 eV	2.01		
tetramer	B	B_T_	S_1_	3.50 eV	0.01	0.28 eV	0.00 eV
			S_4_ ^a^	3.78 eV	5.34		
	Y	Y_T_	S_1_	3.02 eV	0.002	0.40 eV	0.48 eV
			S_4_ ^a^	3.42 eV	4.17		
	Geom.^b^ = Y Arrang.^b^ = B	B_T_(Y_M_)	S_1_	3.07 eV	0.13	0.42 eV	
			S_5_ ^a^	3.49 eV	4.10		

a x is the state with the highest oscillator strength.

b Geom. = geometry, Arrang. = arrangement.

Monomer: lowest vertical transition energy (E_vert_(S_1_)) with oscillator strength (f). Tetramer: lowest (E_vert_(S_1_)) and most intense transition (E_vert_(S_*x*_)) with the corresponding f. Exciton splitting is defined as ΔE_ES_ = E_vert_(S_*x*_) – E_vert_(S_1_); the crystal shift as ΔE_cryst_ = E_vert_(S_1, tetramer_) – E_vert_(S_1, monomer_).

Furthermore, the TD-DFT tetramer calculations reproduce the experimentally observed relative increase in exciton splitting (ΔE_ES_, Table 3) from B- to Y-phase, confirming that the Y-phase exhibits stronger excitonic coupling, being close to an ideal H-aggregate (for more details, see Supplementary Note 4 in the Supporting Information). In parallel, the rate constant analysis reveals that the substantial reduction of Φ_F_ in the Y-phase originates exclusively from a decreased radiative rate constant (*k*_r_), while the nonradiative rate (*k*_*n*r_) remains similarly small in both polymorph crystals (Table 2), owing to minimized quenching via internal conversion and trap-assisted processes.^35,38,40,51^ This reduction of *k*_r_ in Y is consistent with the significantly reduced *f*_1_ calculated by TD-DFT (Table 3) due to nearly ideal H-aggregation;^35,52,53^ for a more detailed discussion, see also Supplementary Note 5 in the Supporting Information. Moreover, the presence of a distinct rise time in the Y-phase (τ_rise_ ≈ 5.9 ns; Figure 5 and Figure S14)—absent in the B- and C-phases—provides evidence for dynamical excimer formation and relaxation in a nearly perfect π-stacked arrangement. In all, we emphasize once more that the large spectral shift between the αDDDCS polymorphs results from a combination of three factors, being (i) a rather large oscillator strength of the parent monomer, (ii) small interplane separations in the range of about 3.3 Å, and (iii) strong geometry change between the polymorphs, i.e., going from strongly twisted to planar structures.

Finally, the similarity between the C- and B-phases in absorption, PL spectra, and PL decay traces provides photophysical support for the B-like μ-HB-type framework inferred from the VT-PXRD analysis (see also Supplementary Note 6). This assignment is in line with the diffraction and microscopy results showing only minor structural changes during the B → C transformation, including slight contraction along the interplane-separation direction and reduced x-slip. Such subtle lattice and registry changes may be accompanied by limited planarization of the molecular torsion, accounting for the minor bathochromic shift observed for the C-phase relative to the B-phase.

### Section 4: Thermoelastic Transition Mechanism

Building on the structural analysis above, we examined the thermoelastic transition mechanism using selected-area electron diffraction (SAED) and molecular dynamics (MD) simulations. SAED of the B-phase (Figure 6a,b) confirms that the crystal diagonals align along the [010] and [001] directions. The crystal edges are assigned to the (011) and (01–1) planes, and the interfacial angle between these planes (86.4° and 93.6°, SCXRD) agrees well with the measured corner angles of the crystal (85.5 ± 0.4° and 94.2 ± 0.9°). During the B → C transition, the diagonal lengths change only marginally (−1.3% along *b* and +1.2% along *c*), while the corner angles remain essentially invariant, indicating preservation of the overall crystal geometry. This behavior is consistent with the B-like μ-HB-type packing framework inferred from the VT-PXRD/Pawley analysis and the closely related twisted molecular geometry supported by the photophysical analysis in Section 3.

**Figure 6:**
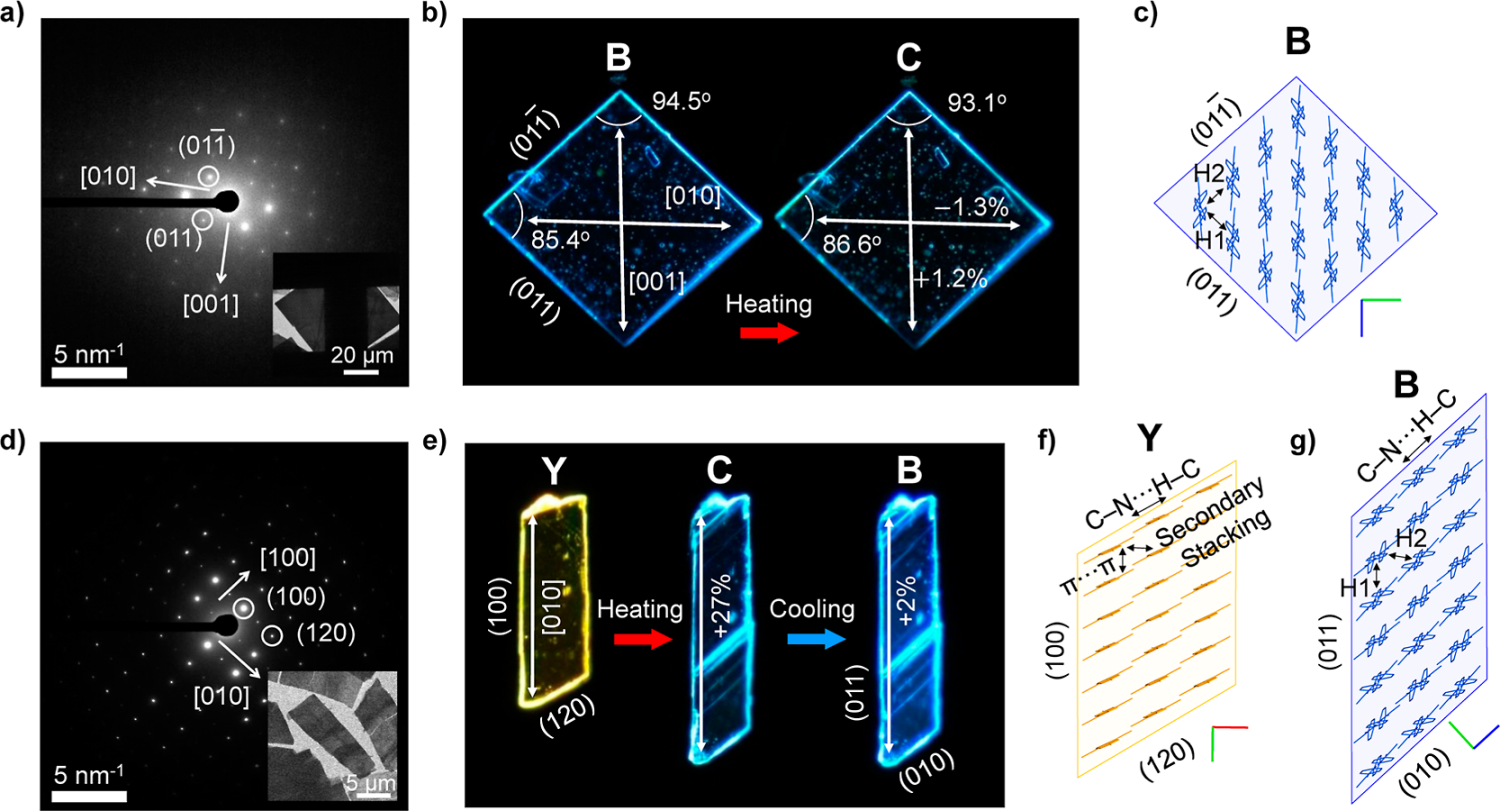
Thermoelastic transitions and deformation behavior of αDDDCS crystals. (a) Lattice orientation of the B-phase from SAED; the inset shows the corresponding transmission electron microscopy (TEM) image. (b) Fluorescence micrographs of B- and C-phase crystals. (c) Molecular packing structure viewed along the *a*-axis, with habit planes and lattice directions assigned from SAED and SCXRD. (d) Lattice orientation of the Y-phase from SAED; the inset shows the corresponding TEM image. (e) Fluorescence micrographs of the Y → C → B transition. (f,g), Molecular packing structures of the Y- and B-phases viewed along the *c*- and *a*-axes, respectively. Habit planes and lattice vectors are assigned based on SAED and SCXRD.

We now focus on the motif-converting Y → C transition, where twist elasticity drives substantial structural reorganization. SAED analysis shows that the long axis of the elongated plate-like Y-phase crystal is parallel to the crystallographic *b*-axis, identifying this direction as the primary π-stacking axis (Figure 6d–f). VT-FM measurements reveal that the crystal elongates by ∼27% during Y → C and by an additional ∼2% during C → B, yielding a total ∼29% increase relative to the Y-phase. Although the Y- and B-phases exhibit metrically similar unit cells (Table 1), this large macroscopic deformation is more clearly understood by considering a common supercell framework that preserves one-to-one molecular correspondence across the transition.^15^ Using the following transformation matrices, we obtained the corresponding common supercells summarized in Table 4.

**Table 4:** Crystallographic Parameters of the Common Supercells Constructed for the Y- and B-Phases

phase	*a’*	*b’*	*c’*	α′	β′	γ′	*V’*
Y	11.520 Å	7.630 Å	48.274 Å	89.19°	87.29°	89.12°	4237.54 Å^3^
B	9.835 Å	9.835 Å	43.063 Å	90.31°	89.69°	86.37°	4156.99 Å^3^

$A_{Y}=\left(\begin{array}{ccc} 2 & 0 & 0\\ 0 & 1 & 0\\ 0 & 0 & 1 \end{array}\right)$ for the Y-phase, and $A_{B}=\left(\begin{array}{ccc} 0 & 1 & -1\\ 0 & 1 & 1\\ 1 & 0 & 0 \end{array}\right)$ for the B-phase.

Within this common supercell framework, the [010] vector of the Y-phase maps onto the [011] direction of the B-phase (corresponding to the *b’* of Table 4), with the corresponding lattice vector increasing from 7.63 to 9.83 Å (28.8%), in excellent agreement with the total macroscopic elongation after completion of the Y → C → B pathway. This mapping demonstrates that the primary π-stacking axis of the Y-phase converts into one of the herringbone axes (H1) of the B-phase, while the secondary stacking direction aligns with the other (Figure 6f,g). Overlay analysis further reveals that the corresponding center-to-center displacement between neighboring molecules along this direction is ∼1.10 Å (Figure S20), indicating that the relatively limited molecular-scale motion can collectively generate large macroscopic deformation. Notably, the lateral C–N···H–C interaction network forming molecular sheets is preserved across the transformation, indicating that key intermolecular contacts survive the packing motif conversion.

To elucidate how a densely packed π-stack reorganizes into a μ-HB motif, we performed MD simulations. Although the simulated transition temperature is lower than in experiment due to finite-size effects, the transformation pathway is preserved. In the low-temperature NVT segment (0–30 ps), the π-stacking, secondary stacking distances, and all four dihedral angles remain essentially constant (Figure 7a–d, Figures S21, S22). For clarity, the four torsional coordinates are relabeled as θ_i,top_, θ_i,bottom_, θ_o,top_, and θ_o,bottom_ according to their vertical positions in the simulation cell (Figure 7a). Upon entering the NPT segment (from 30 ps), this stability breaks down abruptly. Unlike the experiment—where a gradual ∼4% expansion of the π–π distance and a pretransition PL blue shift reveal an elastic-softening (Figure S23)—the simulation exhibits an abrupt expansion of the slipped π-stack. Upon entering the NPT segment, the system rapidly enters a transient state in which all four dihedral angles broaden sharply (e.g., θ_o,top_ spanning −50° to +50° at 40 ps; Figure 7d) accompanied by a sharp rise in their standard deviations (σ; Figure 7c). This collective broadening indicates that accumulated torsional strain has surpassed the stability limit of the slipped π-stack, triggering torsional unlocking.

**Figure 7:**
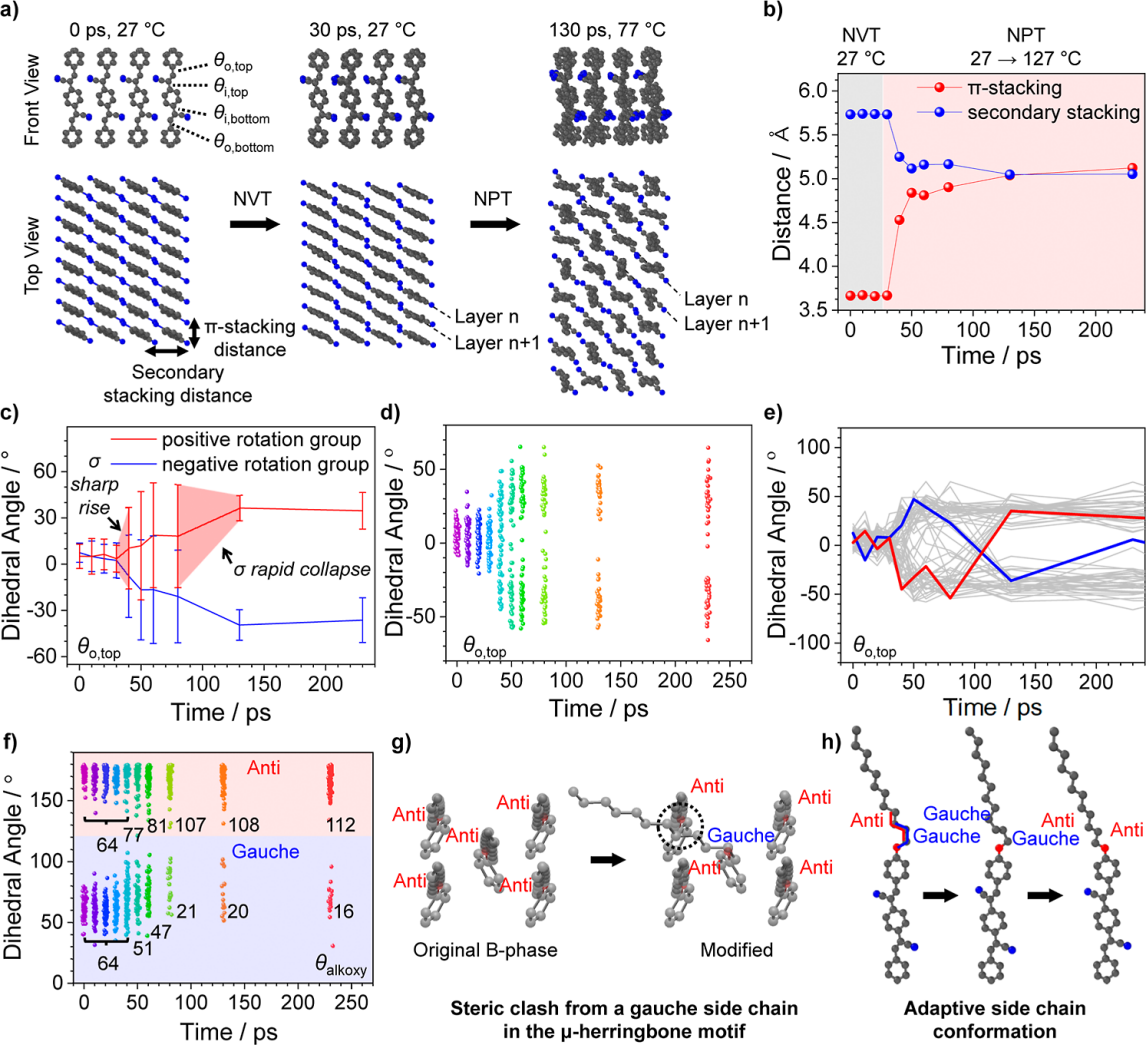
MD simulation of the Y → C transition mechanism. (a) Structural snapshots capturing a stable π-stack at NVT (0 ps), followed by stability breakdown at the onset of NPT (30 ps) and formation of a layer-correlated μ-herringbone arrangement (130 ps). (b) Changes in π-stacking and secondary stacking distances. (c) Mean and standard deviation of θ_o,top_. (d) Distributions of θ_o,top_ for 64 molecules. (e) Individual θ_o,top_ traces for 64 molecules. (f) Dihedral angle distributions of 128 side chains. (g) Model structure of steric clash caused by a gauche side chain in the μ-herringbone motif (dotted circle). (h) Side-chain conformations illustrating how gauche side chains avoid steric clash.

Although this transient state is experimentally short-lived, the simulation captures it clearly and reveals that torsional unlocking is highly correlated rather than random. Molecules within each C–N···H–C-linked intralayer twist in the same direction, while adjacent layers twist oppositely (layer *n* and *n* + 1 in Figure 7a), establishing a cooperative, layer-correlated twist that seeds μ-HB formation. Time-resolved analysis of all four dihedral angles (Figure S24) shows that, shortly after onset, most molecules adopt this correlated torsional rotation pattern. Representative trajectories of θ_o,top_ for all 64 molecules (Figure 7e) reveal rapid segregation into two oppositely signed groups, with only a few transient outliers briefly deviating due to thermal fluctuations before returning to the correlated rotational states (see red and blue traces in Figure 7e). By ∼130 ps, nearly all molecules partition into two equal populations (32/32), indicating the emergence of collective order. This evolution is quantitatively supported by the sharp rise and subsequent collapse of torsional variance (σ; Figure 7c and S21) as the system transitions from transient unlocking to a correlated lock-in state. Concurrently, the π-stacking distance and secondary stacking distances converge (Figure 7b), indicating completion of the symmetric μ-HB metric. Together, these results demonstrate that μ-HB formation arises through a fully cooperative torsional reorganization stabilized by layer-by-layer correlation.

Side-chain reorientation accompanies the transition but likely functions primarily as an adaptive stabilizer rather than a driving force. During the transient state, the population of gauche conformers decreases, and after completion of the transition, most side chains adopt the anti conformation (120° criterion; Figure 7f), consistent with the steric requirements of the μ-HB geometry. A hypothetical B-phase structure with a retained gauche side chain (Figure 7g) reveals steric clash (dotted circle), indicating that gauche-to-anti conversion facilitates formation of the μ-HB geometry. Nevertheless, a small fraction of molecules transiently retain nominally gauche conformations even at ∼130 ps; these avoid steric conflict through compensatory adjustments of other torsional coordinates (Figure 7h). Thus, side-chain motion flexibly accommodates packing conversion by minimizing steric frustration rather than dictating the transition pathway.

As an experimental indicator of alkyl-chain disordering and conformational rearrangement, we examined the intensity ratio of the 2885 cm^–1^ ν_a_(CH_2_) band to the 2850 cm^–1^ ν_s_(CH_2_) band, *I*[ν_a_(CH_2_)]/*I*[ν_s_(CH_2_)], by variable-temperature Raman spectroscopy (Figure S25).^54^ During the Y → C transition, this ratio decreases, indicating increased rotational and conformational disorder of the terminal alkyl chains at elevated temperatures. A similar but more pronounced change was observed during the B → C transition, consistent with the more ordered all-anti nature of the initial B-phase. These observations support the involvement of alkyl-chain disordering in both transitions and suggest that side-chain reorientation is closely associated with an adaptive response that accommodates packing motif conversion and/or thermally activated molecular motion.

Taken together, the MD simulations—interpreted alongside experimental evidence for pretransition elastic softening—show that the twist-elasticity-assisted packing motif conversion is not a simple geometric adjustment but a fully cooperative martensitic reorganization. The process begins with elastic softening of the π-stack as thermal libration accumulates, possibly assisted by low-frequency lattice vibrations or phonon softening, as suggested in related dynamic crystal systems.^15,55–57^ Once the stability limit of the π-stack is exceeded, torsional unlocking ensues, followed by development of a layer-correlated cooperative twist in which molecules within each C–N···H–C-linked layer twist in the same direction while adjacent layers rotate oppositely, establishing the μ-HB registry. As the primary and secondary stacking distances converge, torsions settle into a correlated lock-in state that is consistent with the μ-HB metric, while side-chain reorientation proceeds in parallel as a likely adaptive stabilizer that mitigates steric hindrance. Overall, this framework provides an atomistic view of a twist-elasticity-driven martensitic transition, revealing how coordinated torsional dynamics reorganize a densely packed lattice while largely preserving structural integrity.

### Section 5: Mechanosalient Transitions

The sporadic occurrence of the B → Y transition during heating raises a key question regarding the phase hierarchy of this system. While DSC and VT-FM establish that the C-phase becomes thermodynamically favored over B- above ∼70 °C and over Y-phase above ∼90 °C, they do not resolve the relative stability of B- and Y-phases at ambient condition. Gibbs free energy calculations (see the DFT calculation section in SI) yield −71.8 kcal/mol for the B- and −72.1 kcal/mol for the Y-phase, indicating that the Y-phase is slightly more stable at room temperature (Figure 8a). Nonetheless, the B-phase persists metastably, likely due to substantial steric barriers imposed by its tightly packed μ-HB lattice, which hinder the large torsional rearrangements required for the B → Y conversion. Indeed, thermal annealing slightly below the B → C transition temperature did not induce observable B → Y conversion (Figure S26), supporting that this transition is strongly kinetically frustrated and not readily triggered by thermal activation alone.

**Figure 8:**
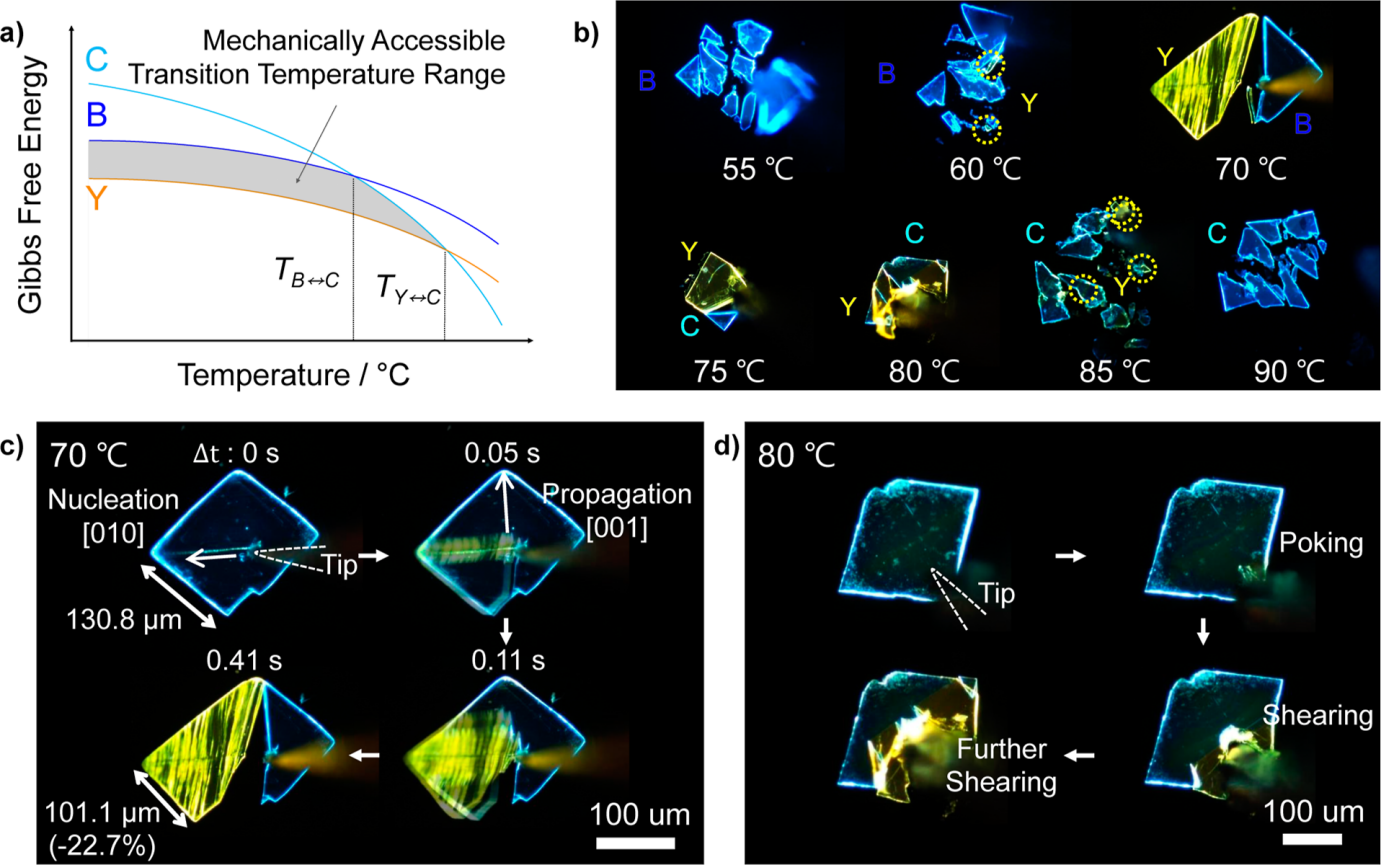
Mechanically induced transitions of αDDDCS crystals. (a) Schematic Gibbs free energy diagram of the αDDDCS polymorphs, constructed from DSC data and computational analysis. The gray region denotes the temperature range where B → Y or C → Y transitions are expected to be mechanically accessible. (b) Mechanical probing test on αDDDCS crystals under varying temperatures. Mechanically induced transitions were observed in the B-phase at 60–70 °C and in the C-phase at 75–85 °C. Regions where the Y-phase locally formed at 60 and 85 °C are marked with dotted circles. (c) At 70 °C, the B → Y transition is initiated by mechanical probing-induced nucleation along the [010] direction and autonomously propagates along the [001] direction. The observed deformation, calculated as Δ*L*/*L*_0_ × 100, is −22.7%, smaller than the +29% value for the Y → B transition, due to the difference in the reference length *L*_0_: in this case, taken from the B-phase rather than the Y-phase. (d) At 80 °C, the C → Y transition displays a ferroelasticity-like response, wherein Y-phase formation occurs only within regions subjected to mechanical shear.

These considerations suggest that the B/C → Y transitions may proceed via a mechanosalient pathway when subjected to appropriate mechanical perturbation. Localized mechanical probing between 70 and 85 °C reveals pronounced mechanochromic and mechanosalient responses (Figure 8b, Movie S12). Below 60 °C, B-phase crystals fracture brittly upon mechanical probing; however, thermal expansion at elevated temperatures appears to reduce internal constraints, enabling access to the B → Y pathway. Above, 60 °C, small yellow-emissive fragments emerge, and by 70 °C, nucleation along the [010] is followed by rapid autonomous propagation, accompanied by a pronounced contractile strain of −22.7% and line-defect formation (Figure 8c). The high defect density in the resulting Y-phase likely reflects substantial interfacial stress associated with the large intramolecular torsional rearrangements.

The C → Y transition exhibits a distinct response. At 75 °C, mechanical probing induces diagonal splitting followed by spontaneous propagation, whereas at 80 °C, the system enters a ferroelastic-like regime requiring sustained external input for continued advancement (Figure 8d, Movie S12). No transition occurs at 90 °C, consistent with thermodynamic stabilization of the C-phase. These temperature-dependent behaviors highlight the interplay between mechanical activation and thermodynamic driving force.

Viewed mechanistically, B/C → Y transformations represent reverse pathways of the thermoelastic mechanism. When the Y-phase is thermodynamically favored, shear applied along the [010] direction—between adjacent C–N···H–C-linked layers—can be accommodated through torsional-to-planar adjustment, promoting Y-phase formation. Movie S13 directly illustrates layer-by-layer phase front propagation under shear along [010], whereas shear applied in arbitrary directions results only in fracture (Movie S14). Together, these results establish that packing motif conversion can be mechanically activated when not thermodynamically prohibited, providing a direct link between twist elasticity and mechanosalient behavior in molecular crystals.

## Conclusions

In summary, we demonstrate that αDDDCS represents a rare thermoelastic molecular martensite governed by cooperative solid-state twist elasticity. The unusually large thermoelastic strain (∼27%) arises from a concerted torsional reorganization coupled with a π-stack to μ-herringbone packing conversion that proceeds in a martensitic, diffusionless manner. Photophysical and quantum-chemical analyses indicate that the pronounced polymorph-dependent spectral shift (ΔE_em_ = 0.39 eV) is governed primarily by changes in torsional molecular geometry, while aggregate symmetry modulates the excitonic response; within this framework, the C-phase can be consistently described as retaining a B-like distorted H-type μ-herringbone arrangement with only modest torsional relaxation. Variable-temperature experiments and MD simulations further reveal a cooperative sequence of softening, torsional unlocking, and layer-correlated twisting and demonstrate that the reverse packing conversion can be mechanically triggered. Taken together, these results highlight conformational twist as a practical molecular design strategy for achieving high-strain responsiveness and mechanically accessible packing conversion in dynamic crystals.

## Supplementary Material

































## Notes

Details of materials, synthesis, characterization, crystal growth, photophysical measurements, single-crystal and powder X-ray crystallography, DFT calculations, and MD simulations; lattice parameters of the B- and Y-phases; TD-DFT results; NMR spectra; VT-FM images; PL, PLQY, and TR-PL data; *p*-XRD patterns; and MD simulation results (ZIP)
(MP4)
(MP4)
(MP4)
(MP4)
(MP4)
(MP4)
(MP4)
(MP4)
(MP4)
(MP4)
(MP4)
(MP4)
(MP4)
(MP4)
(PDF)
