# Supplementary material for: Large Thermo- and Mechanosalient Actuation via Cooperative Twist Elasticity-Induced Packing Motif Conversion
Source: J Am Chem Soc. 2026 Jul 1;148(27):28602–15. doi: 10.1021/jacs.6c05413 (PMC13383629; doi:10.1021/jacs.6c05413)
Supplement: Supplementary file 16 [file ja6c05413_si_016.pdf]

Supporting Information

Large Thermo- and Mechanosalient Actuation via Cooperative Twist Elasticity-Induced Packing-Motif Conversion

Kyoungtae Hwang,[a] Indranil Bhattacharjee,[c] Sooyeon Ra,[a] Jin Hyeok Jang,[a] Jinwoo Park,[b] Haseong Kim,[b] Minwoo Jang,[a] Min Wook Lee,[a] Dong Ryeol Whang[d], Dohyun Moon,[e] Johannes Gierschner,[c]* Hyungbum Park,[b]* Sang Kyu Park[a]**

a Institute of Advanced Composites Materials, Korea Institute of Science and Technology (KIST), Wanju, Jeonbuk 55324, Republic of Korea

b Department of Mechanical Engineering, Incheon National University, Incheon 22012, Republic of Korea

c Madrid Institute for Advanced Studies, IMDEA Nanoscience, C/ Faraday 9, Ciudad Universitaria de Cantoblanco, 28049 Madrid, Spain

d Department of Electronic Materials Engineering, Hoseo University, Asan 31499, Republic of Korea

e Beamline Department, Pohang Accelerator Laboratory (PAL)/POSTECH, Pohang 37673, Republic of Korea

Table of Contents

1. Methods Pg. 2
2. Supplementary Note 1 Pg. 12
3. Supplementary Note 2 Pg. 14
4. Supplementary Note 3 Pg. 16
5. Supplementary Note 4 Pg. 17
6. Supplementary Note 5 Pg. 18
7. Supplementary Note 6 Pg. 20
8. Synthesis Pg. 23
9. Supplementary Tables Pg. 24
10. Supplementary Figures Pg. 32
11. Captions for Supplementary Movies Pg. 60
12. References Pg. 62

1. Methods

Materials

All reagents were obtained from commercial suppliers (Aldrich, Acros, or TCI) and employed without additional purification. 4-hydroxybenzaldehyde (98%, Sigma-Aldrich), 1,4-phenylenediacetonitrile (99%, Sigma-Aldrich), potassium iodide (≥99%, Sigma-Aldrich), potassium carbonate (≥99%, Sigma-Aldrich), anhydrous N,N-dimethylformamide (99.8%, Sigma-Aldrich), tert-butanol (≥99.0%, Sigma-Aldrich), tetrabutylammonium (1 M in methanol, Acros), and 1-bromododecane (>98%, TCI) were used for the synthesis of αDDDCS, following a previously reported procedure.[1]

General methods

The synthesized compound was characterized by a combination of spectroscopic and thermal analysis techniques. ^{1}H-NMR spectra were acquired on an Agilent 600 MHz spectrometer, and the molecular mass of αDDDCS was determined by MALDI-TOF/TOF™ mass spectrometry (5800 system). Thermal behavior was assessed using differential scanning calorimetry (TA Instruments Q20). Thermoelastic and mechanosalient properties of αDDDCS crystals were observed under a polarized optical microscope (Eclipse LV100N POL, Nikon) equipped with a high-speed digital camera (DS-Fi3, Nikon) and a temperature-controlled stage (LTS420, Linkam Scientific). To simultaneously observe the fluorescence of blue and yellow, VT-FM was performed by using long-pass filter (UV-2A, Nikon) with light source (Nikon Intensilight C-HGFI). The crystallographic axes in the single crystal were determined using a 200 kV Glacios cryo-TEM (Thermo Fisher Scientific). For analysis of alkyl side chain disordering, VT-Raman spectra were collected using an InVia Raman microscope (Renishaw, UK) equipped with a 1200 g/mm grating and 514 nm (B-phase) or 785 nm (Y-phase) excitation

lasers, with temperature controlled using an LTS420 temperature-control stage. To evaluate the mechanical properties of the B- and Y-phase single crystals, nanoindentation measurements were carried out using an iNano nanoindenter (Nanomechanics Inc., now KLA Corporation, USA) equipped with a Berkovich diamond indenter. The single crystals were fixed onto SiO_2 substrates using epoxy glue, and indentation experiments were performed at room temperature on the exposed crystal faces. Prior to measurement, the indenter area function and frame compliance were calibrated using a fused silica standard. The contact stiffness, elastic modulus, and hardness were extracted from the load–displacement curves using the Oliver–Pharr method.

Crystal growth

Single crystals of αDDDCS were grown via the liquid–liquid diffusion method. Using a glass vial, αDDDCS was dissolved in dichlorobenzene at concentrations below 1 mg/mL for the B-phase, and at concentrations above 6 mg/mL, predominantly yielding the Y-phase, although minor B-phase domains were occasionally observed. An equal volume of methanol was carefully layered on top of the αDDDCS solution. The vial was tightly sealed with plastic wrap and stored in the dark at room temperature. After the crystals reached a suitable size, they were collected by vacuum filtration using a Buchner funnel and filter flask, and subsequently dried under ambient conditions.

Load-cell measurements

αDDDCS Y-phase single crystals were positioned such that approximately half of the crystal length was supported on a cleaned SiO_2 substrate, after which one end was fixed using epoxy glue and sufficiently cured at ambient temperature. The prepared samples were mounted on an

LTS420 Linkam stage, and the free end of the crystal was carefully positioned to approach, but not initially contact, a miniature load cell (LSB-200, Futek) using a manual XYZ stage equipped with the load cell. After alignment, the samples were heated using the Linkam stage to a temperature immediately below the Y→C transition and allowed to stabilize sufficiently. The Y→C transformation was then induced by additional heating while the force evolution was monitored in real time. The measured force signal was amplified using a Futek IAA105 amplifier, and data acquisition was carried out using a DATAQ DI-2108-P system. Upon reaching the Y→C transition temperature, the crystal came into contact with the load cell and began to exert force. The maximum force was extracted from the resulting force–time curve and subsequently normalized by the crystal volume to obtain the force density.

Glass-plate pushing experiments

Glass-plate pushing experiments were performed using a configuration similar to that employed for the load-cell measurements. Y-phase single crystals were positioned such that approximately half of the crystal length was fixed using epoxy glue onto imide tape attached to a glass substrate. The prepared samples were mounted on an LTS420 Linkam stage and a cleaned glass plate with a precisely measured mass was carefully positioned to approach, but not initially contact, to the free end of the crystal. The samples were heated using the Linkam stage while the entire process was monitored and recorded using an optical microscope (Eclipse LV100N POL, Nikon). During heating, the displacement of the glass plate and the presence or absence of crystal bending were carefully examined to evaluate the force-generation capability and mechanical stability of the crystals during the Y→C transformation. The maximum pushing force was estimated from the maximum static friction force (f_s^{max}) between the two glass surfaces, following the approach reported by Duan et al.[2] The static friction coefficient

(μ_s) between the glass surfaces was assumed to be 0.9, and the force was calculated according to the following equation:

$$F = f_s^{max} = \mu_s m g$$

where m is the mass of the glass plate and g is the gravity acceleration.

Bead-displacement measurements

Bead-displacement measurements were performed using a sample configuration similar to that employed for the glass-plate pushing experiments.[2] Y-phase single crystals were prepared in the same manner; however, instead of a glass plate, zirconia beads with masses estimated by volume were manually positioned such that they were in contact with the free end of the crystal opposite to the epoxy-fixed side. The samples were heated using the Linkam stage while the entire process was recorded using an optical microscope (Eclipse LV100N POL, Nikon). The velocity (v) of the beads displaced during the Y→C transformation was subsequently determined from the recorded optical images. Using the estimated bead mass (m), the kinetic energy (E_k) was calculated according to the following equation:

$$E_k = \frac{1}{2} m v^2$$

The resulting kinetic energy was normalized by the crystal volume to estimate the work density.

Photophysical characterization

The αDDDCS solution in THF was prepared at a concentration of 2 × 10^{-5} mol L^{-1}. Nanocrystal (NC) suspensions of the B and Y-phase were prepared by dispersing the crystals in distilled H_2O (20 mg mL^{-1}) via bath sonication for 1 hour. To minimize reabsorption and

scattering, the suspensions were filtered through a syringe filter (pore size ≤ 5 μm) to remove large particles. The C-phase suspension was obtained by heating the B-phase suspension during measurements.

UV-Vis absorption spectra of αDDDCS solution and the NC suspensions were measured by a V-670 spectrophotometer (JASCO). Photoluminescence (PL) spectra of αDDDCS solution and the NC suspensions were acquired by a NanoLog spectrofluorometer (Horiba Jobin Yvon). For single-crystal samples, PL spectra were obtained by an FS5 spectrofluorometer (Edinburgh Instruments). All PL spectra were corrected for the wavelength characteristics of the detection unit. Time-resolved photoluminescence (TRPL) decays were recorded on an FS5 spectrofluorometer using time-correlated single-photon counting (TCSPC). A 375 nm pulsed diode laser (EPL-375) was used as the excitation source, and the decay profiles were accumulated until the peak intensity reached 10,000 counts. The absolute PL quantum yields (QYs) of the single crystals were determined in the FS5 spectrofluorometer setup, using a calibrated integrating sphere (SC-30).

X-ray crystallography (Single crystal)

For the SCXRD of the αDDDCS crystal, the crystal was directly picked up from the as-synthesized batch with a Mitegen loop attached to goniohead, and transferred to a nitrogen stream (298 and 100 K). The data collection was carried out using a synchrotron-based X-ray source produced from a PLS-II 2D bending magnet with a Si (111) double crystal monochromator (0.70000 Å) and Rayonix MX225HS CCD area detector. The PAL BL2D-SMDC program[3] was used for one set of data collection at the following conditions: detector distance of 66 mm, 1-axis omega scan with $\Delta\omega$ of 3°, and the exposure time of 1 sec/frame.

HKL3000sm (ver. 717.6)[4] was used for cell refinement, reduction, and absorption correction. The structures were solved by the intrinsic phasing method using SHELXT-2018/2[5] and refined by full matrix least-squares on F^2 using SHELXL-2019/3.[6] All non-hydrogen atoms were refined anisotropically, and all H atoms were placed in geometrically idealized positions and constrained to ride on their parent atoms with C—H = 0.93 – 0.98 Å and with U_{iso} (H) values of 1.2 and 1.5 U_{eq} of the parent atoms. The crystallographic experiment data and refinement parameters are summarized in **Table S2**.

Variable temperature X-ray crystallography (Crystalline powder)

Synchrotron-based powder X-ray diffraction (PXRD) measurements were conducted at the BL2D-SMC beamline of the Pohang Accelerator Laboratory (PAL), Korea. Crystalline samples were loaded into 0.4 mm diameter capillary tubes for analysis. Debye–Scherrer diffraction patterns were collected using a Rayonix MS225HS CCD area detector with a monochromatic X-ray (λ = 0.70000 Å) over a full 360° rotation, with exposure times ranging from 4 to 16 seconds. Temperature variation during measurements was controlled using an Oxford Instruments Cryojet 5 system, and phase transitions were visually monitored via a mounted camera during the heating and cooling. Data collection was carried out using the PAL BL2D-SMDC program, and the two-dimensional diffraction patterns were converted to one-dimensional profiles using the Fit2D program,[7] based on calibration measurements with a NIST Si 640c standard sample.

Structural analysis of the high-temperature C-phase was performed using the Reflex Plus module implemented in the Materials Studio software package. The experimental PXRD pattern collected at 378 K was indexed using the X-Cell algorithm,[8] and Pawley refinement

was subsequently carried out to refine the lattice parameters, peak profile, and line-shape parameters.[9] Peak profiles were modeled using a Pseudo-Voigt function, and asymmetry corrections were applied using the Berar–Baldinozzi function.[10] For the C-phase refinement, a monoclinic P2/c space group was employed (Rel. FOM = 0.408, FOM = 206, 28 peaks indexed out of 28 detected peaks, impurity = 0). The refined lattice parameters were determined to be a = 6.7369 Å, b = 47.654 Å, c = 7.0282 Å, β = 90.65°, and V = 2256.2 $Å^3$, with agreement factors of R_{wp} = 4.39% and R_p = 2.30%. The calculated PXRD pattern showed good agreement with the experimentally observed diffraction pattern in both peak position and relative intensity.

DFT calculations

The molecular geometry of αDDDCS was fully optimized (F_M in **Table 3**) by density functional theory (DFT), in the C_2 symmetry point group, using the B3LYP functional and the 6-31G(d,p) basis set, as implemented in the Gaussian 16 program package.[11] For the optimization in the crystal geometries of the B and Y phase (B_M and Y_M in **Table 3**), the torsional angles around the inner and outer single bonds in the vinylene unit ($\theta_{i,o}$) were fixed to those found in the respective x-ray structures, while all other coordinates were fully relaxed during optimization. For the tetramer calculations, nearest neighbor tetramer clusters were extracted from the x-ray structure, and subsequently replaced by the partially optimized B_M and Y_M molecules, respectively (B_T and Y_T in **Table 3**); additionally, for $B_T(Y_M)$, the B-phase tetramer arrangement was replaced by Y_M molecules. All single point time-dependent (TD) DFT calculations were performed with the CAM-B3LYP functional and 6-31G(d,p) basis set within the Gaussian 16 program package. The crystal shift ΔE_{cryst} was estimated from the

lowest excited state (S_1) of small tetramer clusters relative to that of the fully relaxed monomer, according to the following equation:

$$\Delta E_{cryst} = E_{vert}(S_1, \text{tetramer}) - E_{vert}(S_1, \text{monomer}) \quad (1)$$

Furthermore, DFT calculations were performed using the DMol3 module in Materials Studio 2024, to evaluate the thermodynamic stability of the B-phase and Y-phase structures. Dispersion interactions were accounted for by employing the Tkatchenko–Scheffler (TS) correction within the DFT-D framework.[12] Geometry optimizations were first carried out for each phase prior to thermochemical analysis. The exchange–correlation energy was described by the Perdew–Burke–Ernzerhof (PBE) functional within the generalized gradient approximation (GGA)[13], and all calculations were conducted under an all-electron scheme. The double numerical plus polarization (DNP) basis set was used (basis file 4.4). Self-consistent field (SCF) convergence was achieved using an SCF tolerance of 1.0×10^{-8}, with density mixing applied via a charge-mixing parameter of 0.2. To accelerate SCF convergence, direct inversion in the iterative subspace (DIIS) was enabled with a DIIS size of 6. Geometry optimization was performed in Cartesian coordinates. The convergence thresholds were set to an energy change of 1.0×10^{-5} Ha, a maximum force of 0.002 Ha/Å, and a maximum displacement of 0.005 Å, with a maximum step size of 0.3 Å. Under these conditions, approximately 3000–4000 optimization iterations were typically required to obtain fully optimized structures. Following structural optimization, vibrational frequency calculations were performed for the optimized geometries to obtain thermochemical quantities. Based on the frequency-derived thermochemical corrections, the Gibbs free energy at 298.15 K was evaluated using the $G(T) = H(T) - TS(T)$ formalism, where $H(T)$ and $S(T)$ were determined from the vibrational contributions within the harmonic thermochemical treatment implemented in

DMol3. The relative stability of the B-phase and Y-phase under ambient conditions was assessed by comparing the resulting Gibbs free energies.

MD simulations

Molecular dynamics (MD) simulations were conducted using the LAMMPS package[14] (version 29 Aug 2024). The simulation domain was created by replicating the Y-phase crystallographic unit cell into a 4 × 4 × 2 supercell, containing a total of 64 molecules. To describe both intra- and intermolecular interactions, the GAFF2.1 force field[15] was employed. Atomic partial charges were determined via the electrostatic potential (ESP) fitting method, with underlying electronic structures calculated using density functional theory (DFT) at the B3LYP/cc-pVDZ level of theory, as implemented in the ORCA software [16, 17] suite. For non-bonded interactions, the Lennard-Jones potential was treated with a switching function initiated at 9.0 Å and a final cutoff distance of 15.0 Å. The Coulombic interactions were evaluated using a real-space cutoff of 15.0 Å, while long-range electrostatic interactions were treated using the Ewald summation method[18, 19]. The relative root-mean-square (RMS) error for per-atom force in long-range electrostatics calculation was maintained at 1.0×10^{-8}. Prior to the dynamic runs, the simulation cell was transformed into a triclinic geometry and subjected to energy minimization. The convergence thresholds for energy and force were set to 1.0×10^{-7} kcal/mol and 1.0×10^{-7} kcal/mol/Å, respectively. The system reached convergence within approximately 4,000 iterations, within the maximum limit of 10,000 steps. Following minimization, the system was equilibrated in the NVT ensemble at 300 K for 30 ps with an integration time step of 0.1 fs. Stability was confirmed as the potential energy reached a steady state after approximately 5.7 ps. Subsequently, thermal cycling was performed under the NPT ensemble

at a constant pressure of 1.0 atm. The NPT simulations employed a fully flexible triclinic barostat, in which the normal stress component was controlled independently, while the shear components were set to zero, allowing fluctuations in both the cell lengths and tilt factors. The thermal cycle consisted of a heating stage from 300 K to 450 K over 300 ps, followed by a cooling stage back to 300 K over an additional 300 ps. The relaxation time constants for the thermostat and barostat were specified as 100 fs and 1,000 fs, respectively.

2. Supplementary Note 1. Assignment of minor DSC features

A weak endothermic feature is observed near 70 °C during the first heating cycle of the as-prepared Y-phase sample (**Figure 3**a). This feature is not assigned to an independent transition of the Y-phase, but rather to a trace amount of B-phase impurity formed under the Y-phase growth conditions. Fluorescence microscopy of the as-prepared Y-phase powder reveals a small population of blue-emissive crystals within the predominantly yellow-emissive sample (**Figure S4**), indicating the presence of a minor B-phase fraction. Consistent with this assignment, the temperature of the weak endothermic feature coincides with the intrinsic B→C transition observed for the B-phase at 71.5 °C (**Figure 3**c). Thus, the small endothermic event near 70 °C in the Y-phase DSC trace is attributed to the B→C transition of a minor B-phase impurity rather than to an additional polymorphic transition.

The second feature is the marked shift in the Y→C transition temperature between the first and subsequent heating cycles of the Y-phase sample (**Figure 3**a, blue arrow). The as-prepared Y-phase transforms to the C-phase at 94.8 °C, whereas Y-phase generated after prior transformation events—either during subsequent thermal cycles or transiently through mechanically triggered B→Y conversion in the B-phase sample—undergoes the Y→C transition at essentially the same lower temperature. This behavior is attributed to differences in prior thermal and mechanical history, likely involving stress redistribution associated with preceding transformations.[20] Importantly, VT-PXRD confirms that the mechanically generated Y-phase and the directly crystallized Y-phase share the same crystal structure, as evidenced by the characteristic Y-phase reflection appearing at the same 2θ position at 80 °C (**Figure 3**e,f). In addition, the mixed Y/C-phase diffraction pattern observed during B-phase heating can be reproduced by the Y- and C-phase reference patterns without invoking additional reflections (**Figure S5**). These results support that the shift in $T_{Y\rightarrow C}$ reflects history-dependent

transformation behavior of the same Y-phase structure, rather than the involvement of an additional polymorph.

3. Supplementary Note 2. Mechanical output characterization of the Y→C transition

To quantitatively evaluate the mechanical output associated with the large thermoelastic deformation of the Y→C transition, the force density and work density of the Y-phase crystals were investigated using three complementary approaches: (i) load-cell measurements, (ii) glass-plate pushing experiments, and (iii) bead-displacement measurements for estimation of work density, with the latter two methods performed following the approach reported by Duan et al.[2]

Representative force–time curves obtained from the load-cell measurements are shown in **Figure S7**, and the corresponding crystal dimensions and calculated force densities are summarized in **Table S3**. The measured average force density was $3.6 \pm 3.0 \times 10^{8}$ N m^{-3}, which is comparable to the upper range reported for high-performance dynamic molecular crystals.[21-23]

To further validate the force-generation capability, an additional glass-plate pushing experiment was performed.[2] In this method, the force generated by the crystal was estimated from the maximum static friction between stacked glass plates and the glass substrate. Importantly, the evaluation focused on the force range under which the crystal maintained structural integrity without severe bending deformation. As shown in **Figure S8**, **Table S4**, **Movies S9** and **S10**, a Y-phase crystal with a volume of 7.8×10^{-14} m^{3} displaced a glass plate weighing 1.4 mg without noticeable bending, whereas pronounced bending deformation was observed when a 3.7, 2.4, 5.2×10^{-14} m^{3} crystal attempted to displace a heavier glass plate (2.4–9.1 mg). Based on these observations, the threshold for the onset of substantial bending was estimated to lie between force densities of 1.6 and 5.8×10^{8} N m^{-3}. These values are in excellent agreement with the load-cell measurements, supporting the reliability of the estimated force output. Notably, although the Y→C transition is accompanied by slight striation and

microcrack formation, the crystals nevertheless retain sufficient mechanical integrity to generate substantial force without catastrophic fragmentation.

The work density was further estimated using a bead-displacement experiment.[2] Because the Y-phase single crystals were extremely thin and high-aspect-ratio, force transfer from the transforming crystal to the bead was likely inefficient; part of the generated mechanical energy may be dissipated through bending deformation before bead acceleration. Thus, the kinetic-energy-based values should be regarded as conservative estimates of the mechanical work output. Nevertheless, upon the Y→C transformation, Y-phase crystals with volumes of 2.6–9.8 × 10^{-14} m^3 displaced zirconia beads with masses of 0.12–0.15 mg (**Figure S9**, **Table S5**). From the resulting bead kinetic energy, the apparent work density and corresponding power density were estimated to be 63–168 J m^{-3} and 996–5260 W m^{-3}, respectively.

4. Supplementary Note 3: Nanoindentation analysis of the B- and Y-phases

To investigate whether the distinct molecular conformations and packing topologies of the B- and Y-phases influence their mechanical characteristics, nanoindentation measurements were performed on the major accessible crystal faces, namely the (100) face of the B-phase and the (001) face of the Y-phase. Representative load–displacement curves are shown in **Figure S10**, revealing reproducible mechanical responses for both polymorphs. Quantitative analysis based on the Oliver–Pharr method yielded a contact stiffness of 40.6 ± 0.7 mN μm^{-1}, an elastic modulus of 12.5 ± 0.3 GPa, and a hardness of 0.28 ± 0.01 GPa for the B-phase, compared to 35.8 ± 1.4 mN μm^{-1}, 10.2 ± 0.4 GPa, and 0.20 ± 0.01 GPa for the Y-phase, respectively. Although both phases fall within a comparable mechanical regime typical of molecular crystals, the consistently lower modulus and hardness of the Y-phase indicate a somewhat more compliant mechanical response. In addition, the loading curves of the Y-phase exhibit more pronounced pop-in events, suggesting easier activation of localized deformation processes such as slip or defect-mediated structural rearrangement. This behavior is likely associated with the more planar π-stacked packing motif of the Y-phase relative to the twisted μ-herringbone packing of the B-phase. Overall, these results indicate that the distinct molecular conformations and packing topologies of the two polymorphs are reflected not only in their thermoelastic responses but also in their mechanical characteristics.

5. Supplementary Note 4: Exciton Splitting in the αDDDCS polymorphs

The TD-DFT tetramer calculations can also reproduce the trend for the exciton splitting (ΔE_{ES}); to obtain absolute numbers for the latter, however, large number of molecules are required until convergence is reached.[24] In experiment ΔE_{ES} can be estimated as the energy difference of the absorption maxima of the NP spectrum and the onset of emission, amounting 0.97 eV for B, and 1.29 eV for Y, that is an increase of 33%. In the tetramer calculations, ΔE_{ES} can be extracted as the energy difference between the state that carries the highest oscillator strength (**Table 3**) and S_1. This gives 0.28 eV for B_T and 0.40 eV for Y_T, i.e. an increase of 42%, in reasonable agreement with experiment. For the C-phase, whose structure has not been resolved, only the experimental ΔE_{ES} could be determined; this amounts to 0.93 eV, which is very similar to that of the B-phase.

6. Supplementary Note 5: Radiative Dynamics in the αDDDCS Polymorphs

Besides the strong color contrast found in αDDDCS, also the solid-state fluorescence quantum yield and lifetime Φ_F, τ_F are crucial parameters for application of stimuli-responsive materials. In the B-phase, Φ_F is found to be as high as 91% (**Table 2**), and maintains high with 46% in the Y-phase. (**Figure S13**) The origin for the reduction of Φ_F in Y can be elucidated by rate constant analysis, where the radiative and nonradiative rates are extracted as $k_r = \Phi_F / \tau_F$ and $k_r = (1 - \Phi_F)/ \tau_F$. As seen in **Table 2**, k_{nr} are similar for both phases, adopting a small value of 0.014 and 0.025 ns^{-1} for B and Y, respectively; this is in accordance with former results on related DCS compounds,[25] and reflects that the two main quenching pathways in organic solids are minimized in the αDDDCS single crystal, that is (i) internal conversion (IC) due to suppression of large amplitude motion on the path to the conical intersection which governs IC,[25, 26] and (ii) quenching by trap sites at surfaces/interfaces, due to the high purity, relatively large size, and small surface:volume ratio of the single crystals.[27, 28] These factors are expected to be the same for the B- and Y-phase, which is confirmed in the same, low k_{nr} rate. Therefore, the main difference in Φ_F can be traced back to k_r, which in fact is seven time higher in the B-phase (0.14 ns^{-1}) compared to Y (0.02 ns^{-1}). According to the Strickler-Berg relation,[27, 29] this should be directly reflected in a higher oscillator strength f of B compared to Y. This is exactly found in the tetramer calculations, where $f(S_1)$ for B_T is five times higher than Y_T. This large difference is due to the fact that in the Y-phase the conjugated backbones of side-by-side packed nearest neighbors are virtually identical (they differ in the conformation of the side chains, **Figure 4**), so that Y-phase represents an almost ideal H-aggregate, with consequently small $f(S_1)$.[27, 30] On the other side, in the B-phase, side-by-side oriented nearest neighbors adapt a μ-herringbone type H-aggregate, which results in a non-negligible angle between the transition dipole moments, and thus a significant increase of $f(S_1)$.[27, 30] In any case, the very

low k_r arising from the small $f(S_1)$ in the Y-phase can still compete with the low k_{nr} resulting in the reasonably high Φ_F of 46%.

Further inspection of the fluorescence time traces of the polymorphs in **Figure 5** and **Figure S14** reveal a rise time of ca. 5.9 ns for the Y-phase (detected at the emission maximum), while for the B-phase and C-phase this is not observed; this is fully in line with earlier investigations of DCS compound, where rise times (in the order of 1 ns) were found exclusively for nearly perfect π-stacks, with distinct excimer features,[31] just like in the current case. This resembles early works on pyrene single crystals, where rise times were attributed to excimer formation as a subsequent step after photoexcitation.[32, 33] It should be anyway remarked that the rise time in pyrene crystals is much shorter (20 ps at ambient conditions), reflecting smaller activation barriers; the deceleration of this process in DCS systems will be discussed in more detail in a forthcoming work.

7. Supplementary Note 6: Structural Implications for the C-phase

Although full structural refinement of the C-phase by SCXRD was not achieved, VT-PXRD indexing and Pawley-type refinement establish that the C-phase lattice is closely related to that of the B-phase. This lattice-level similarity provides the primary structural basis for assigning the C-phase to a B-like framework, rather than to the π-stacked Y-phase arrangement.

The photophysical data provide independent support for this structural assignment. The optical spectra of the C-phase remain much closer to those of the B-phase than to those of the Y-phase, exhibiting only a modest bathochromic shift in emission (E_{em} = 2.57 eV, 482 nm for the C-phase vs. 2.61 eV, 475 nm for the B-phase) and a minor difference in the NC absorption maximum (E_{abs} = 3.91 eV for the C-phase vs. 3.96 eV for the B-phase). Correspondingly, the overall crystal shift relative to solution remains comparable in magnitude ($\Delta E_{cryst,em}$ = −0.16 eV for the C-phase vs. −0.20 eV for the B-phase; **Table 3**), indicating that the excitonic regime does not change substantially between these two phases.

Further insight is obtained from the experimentally estimated exciton splitting and fluorescence decay behavior. The C-phase exhibits ΔE_{ES} = 0.93 eV, which is very close to that of the B-phase (0.97 eV) and substantially smaller than that of the Y-phase (1.29 eV). Given that ΔE_{ES} is sensitive to excitonic coupling strength and aggregate symmetry, this similarity suggests that the C-phase does not approach the nearly ideal H-aggregate behavior observed in the Y-phase. Rather, it appears to remain within a distorted H-type coupling regime comparable to that of the B-phase. Consistently, the absence of a rise time in the fluorescence decay profile of the C-phase, as in the B-phase and in contrast to the Y-phase, further argues against formation of the nearly perfect π-stacked arrangement associated with excimer-like relaxation.

This photophysical assignment is consistent with the lattice-level relationship between the SCXRD-resolved B-phase and the VT-PXRD-derived C-phase, as well as with the macroscopic dimensional changes observed by optical microscopy. Under the axis transformation $b_B \to c_C$ and $c_B \to a_C$, the C-phase lattice shows only small changes relative to the B-phase (**Table 1**), with a contraction of approximately −2.0% along the interplane separation direction and a nearly unchanged lattice length (+0.1%) along the orthogonal direction. Optical microscopy independently shows small dimensional changes of −1.3% and +1.2% along the corresponding *b*- and *c*-axis directions, respectively (**Figure 6**b), in good agreement with the Pawley-derived lattice changes. A schematic B-/C-phase overlay model constructed from these observed crystal dimensional changes visualizes the modest in-plane lattice distortion associated with the transformation (**Figure S28**). In addition, the low-angle PXRD reflections characteristic of the lamellar framework are retained in the C-phase, while their shift to lower angles indicates a slight increase in the lamellar spacing (**Figure S27**). Together, these observations indicate that the B→C transformation largely preserves the B-like μ-HB-type framework, while allowing only minor adjustments in molecular uprightness, x-slip, and interplane separation.

Within this B-like framework, the modest bathochromic shift of the C-phase relative to the B-phase can be rationalized without invoking a substantial rearrangement of aggregate symmetry. As detailed in the Photophysical Analysis section, because the magnitude of the crystal shift is primarily governed by torsional geometry when the packing framework and interplane separations are comparable, the small lattice and registry changes associated with the B→C transformation may be accompanied by limited planarization of the molecular torsion (**Figure S28**). This subtle geometric adjustment plausibly accounts for the modest red-shift of the emission spectrum in the C-phase relative to the B-phase, while remaining consistent with

the VT-PXRD, dimensional, excitonic, and dynamical evidence. Although full atomic-level refinement of the C-phase will require further crystallographic characterization, the combined evidence supports a C-phase structure that retains a B-like μ-HB-type framework rather than adopting the π-stacked Y-phase arrangement.

8. Synthesis

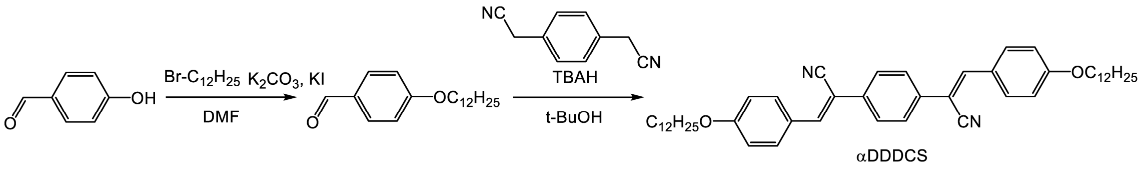

Scheme S1. Synthesis route for obtaining (3) αDDDCS

(2Z,2'Z)-2,2'-(1,4-phenylene)bis(3-(4-(dodecyloxy)phenyl)acrylonitrile) (αDDDCS): αDDDCS was synthesized by following the previously reported procedure.[1] ^{1}H-NMR (600 MHz, $CDCl_3$): δ = 7.90 (d, 4H), 7.71 (s, 4H), 7.52 (s, 2H), 6.97 (d, 4H), 4.02 (t, 4H), 1.79 (m, 4H), 1.44 (m, 4H), 1.27 (m, 32H), 0.87 ppm (m, 6H) (**Figure S1**). MS (MALDI-TOF) (EI, m/z): [M − H]+ calcd for C48H64N2O2, 701.05, found, 700.4.

9. Supplementary Tables

Table S1. Reported deformation for the representative thermoelastic organic crystals.

	Materials	Mechanism	Packing motif	Deformation	Reference
-	αDDDCS (Y→C)	Packing motif change	π-stacking to μ-herringbone	27%	This work
1	Hexamethylbenzene	Molecular sliding	π-stacking	5%	(34)
2	2-(5-(benzo[d]thiazol-2-yl)-1,3,4-oxadia-zol-2-yl)-5-(diethylamino)pheno	Molecular sliding	π-stacking	6%	(35)
3	2DQTT-o-B	Molecular sliding	herringbone	4%	(36)
4	6-Chloronicotinic acid	Molecular sliding	π-stacking	7%	(37)
5	(phenylazophenyl)palladium hexafluoroacetylacetonate	Molecular sliding	π-stacking	10%	(38)
6	[7]helquat	Molecular sliding	π-stacking	~1%	(39)
7	BEzH	Molecular sliding	a partially herringbone	2%	(40)
8	$[Co(NO_3)_2(L)]$	Molecular sliding	π-stacking	7%	(41)
9	Bis(triisopropylsilylethynyl)pentacene	Molecular sliding	π-stacking	10%	(42)
10	5FC-C14	Molecular sliding	π-stacking	15.4%	(43)
11	2'''',3'-diethyl-4,4'''''-dipentyl-1,1':4',1'':4'',1''':4''',1'''':4'''',1'''''-sexiphenyl	Molecular sliding	μ-herringbone	8.2%	(44)
12	Terephthalic acid	Molecular sliding	π-stacking	26%	(20)
13	4,7-Bis[5-(4-nonylphenyl)-2-thienyl]-5,6-dimethoxy-2,1,3-benzothiadiazole	Molecular sliding	herringbone	33%	(45)
14	Guanidinium nitrate	Molecular sliding	-	51%	(21)
15	Ditert-butyl [1] benzothieno[3,2-b][1]1benzothiophene	Molecular rotation	herringbone	~1%	(46)
16	arylgold(I) (N-heterocyclic carbene)	Molecular rotation	π-stacking	10%	(47)
17	(2Z,20Z)-2,20-(1,4-Phenylene)bis(3-(4-bromophenyl)acrylonitrile)	Molecular rotation	π-stacking	6%	(48)

18	Penciclovir	Molecular rotation	π-stacking	3%	(49)
19	$[Ni^{II}(en)_3](ox)$ complex	Molecular rotation	-	5%	(50)
20	2,7-Di([1,1′-biphenyl]-4-yl)-fluorenone	Conformational change	herringbone	10%	(2)
21	Naphthalene-2,3-diyl bis(4-fluorobenzoate)	Conformational change	π-stacking	13%	(51)
22	DNDI	Conformational change	π-stacking	10%	(52)
23	(E)-4-Chloro-N0-(4-(tri-fluoromethoxy)benzylidene)benzo hydrazide	Conformational change	herringbone	8%	(53)
24	6,6'-((1E,1'E)-(butane-1,4-diylbis(azaneylylidene))bis(metha neylylidene))bis(2,4-dichlorophenol)	Conformational change	π-stacking	3.6%	(54)
25	dodecafluorosuberic acid / 1,2-bis(4-pyridyl)ethane	Conformational change	-	~1%	(55)
26	2,7-Dioctylbenzothieno[3,2-b]benzothiophene	Packing motif change	herringbone to π-stacking	13%	(56)
27	3,7-Di([1,1′-biphenyl]-4-yl)dibenzo[b,d]thiophene 5,5-dioxide	Packing motif change	a partially herringbone to a fully herringbone	15%	(57)
28	4-dimethylaminobenzaldehyde(4-cyanophenyl-ethylidene)-hydrazone (E, E)	Packing motif change	herringbone to π-stacking	20%	(58)

Table S2. Single crystal X-ray structures of αDDDCS at temperatures of 298 and 100 K.

Name		B-phase	B-phase	Y-phase	Y-phase
Empirical formula		$C_{48}H_{64}N_2O_2$	$C_{48}H_{64}N_2O_2$	$C_{48}H_{64}N_2O_2$	$C_{48}H_{64}N_2O_2$
Formula weight		701.01	701.01	701.01	701.01
Temperature		298(2) K	101(2) K	297(2) K	100(2) K
Wavelength		0.700 Å	0.700 Å	0.700 Å	0.700 Å
Crystal system		Monoclinic	Monoclinic	Triclinic	Triclinic
Space group		$P2_1/c$	$P2_1/c$	P-1	P-1
Cell Parameters	a (Å)	43.062(9)	42.765(9)	5.7600(12)	5.7010(11)
	b (Å)	7.1710(14)	7.0040(14)	7.6300(15)	7.4030(15)
	c (Å)	6.7310(14)	6.7970(14)	48.274(10)	48.250(10)
	α (°)	90	90	89.19(3)	88.30(3)
	β (°)	90.46(3)	90.39	87.29(3)	87.70(3)
	γ (°)	90	90	89.12(3)	89.92(3)
Volume (Å^3)		2078.4(7)	2035.8(7)	2118.8(7)	2033.8(7)
Z		2	2	2	2
Density (Mg/m^3)		1.120	1.144	1.099	1.145
Absorption coefficient (mm^{-1})		0.064	0.066	0.063	0.066
F(000)		764	764	764	764
Crystal size		0.020 x 0.015 x 0.002 mm^3	0.101 x 0.006 x 0.005 mm^3	0.020 x 0.016 x 0.002 mm^3	0.125 x 0.009 x 0.008 mm^3
Theta range for data collection		1.863 to 24.993°	1.407 to 26.998°	1.664 to 24.999°	1.665 to 26.993°
Index ranges		−51<=h<=51, −8<=k<=8, −8<=l<=8	−55<=h<=55, −8<=k<=8, −8<=l<=8	−6<=h<=6, −9<=k<=9, −58<=l<=58	−7<=h<=7, −9<=k<=9, −59<=l<=57
Reflections collected		12097	11930	12151	12543
Independent reflections		3454 [R(int) = 0.0905]	3630 [R(int) = 0.1448]	6338 [R(int) = 0.0980]	6646 [R(int) = 0.1146]
Completeness to theta = 24.835°		90.4%	75.5%	81.1%	81.6%
Absorption correction		Empirical	Empirical	Empirical	Empirical
Max. and min. transmission		1.000 and 0.895	1.000 and 0.922	1.000 and 0.817	1.000 and 0.890
Refinement method		Full-matrix least-squares on F^2	Full-matrix least-squares on F^2	Full-matrix least-squares on F^2	Full-matrix least-squares on F^2
Data / restraints / parameters		3454 / 0 / 236	3630 / 0 / 237	6338 / 0 / 472	6646 / 0 / 471
Goodness-of-fit on F^2		1.878	0.964	0.894	0.899
Final R indices [I>2sigma(I)]		R_1 = 0.1851 wR_2 = 0.4955	R1 = 0.0850 wR2 = 0.2092	R_1 = 0.0820 wR_2 = 0.1899	R1 = 0.0863 wR2 = 0.1872

R indices (all data)	R_1 = 0.2268<br>wR_2 = 0.5275	R1 = 0.1924<br>wR2 = 0.2816	R1 = 0.2470<br>wR2 = 0.2830	R1 = 0.2540<br>wR2 = 0.2611
Extinction coefficient	0.46(10)	0.043(8)	0.026(4)	n/a
Largest diff. peak and hole	0.609 and −0.485 e·Å^{-3}	0.366 and −0.297 e·Å^{-3}	0.171 and −0.227 e·Å^{-3}	0.291 and −0.243 e·Å^{-3}
CCDC	2537082	2537624	2537081	2537080

Table S3. Crystal dimensions and load-cell-based force-density analysis of Y-phase single crystals during the Y→C transformation.

	Length (m)	Width (m)	Thickness (m)	Volume (m^3)	Force (N)	Force density (N m^{-3})
1	3.5×10^{-4}	8.0×10^{-5}	1.4×10^{-5}	3.9×10^{-13}	5.6×10^{-5}	1.4×10^{8}
2	2.8×10^{-4}	4.3×10^{-5}	7.0×10^{-6}	8.3×10^{-14}	5.3×10^{-5}	6.4×10^{8}
3	3.3×10^{-4}	5.9×10^{-5}	1.1×10^{-5}	2.1×10^{-13}	3.0×10^{-5}	1.4×10^{8}
4	4.0×10^{-4}	5.8×10^{-5}	1.0×10^{-5}	2.4×10^{-13}	1.9×10^{-4}	8.0×10^{8}
5	4.1×10^{-4}	7.1×10^{-5}	1.0×10^{-5}	2.9×10^{-13}	2.2×10^{-5}	7.7×10^{7}

Table S4. Crystal dimensions and glass-pushing-based force-density analysis of Y-phase single crystals during the Y→C transformation.

	Glass weight (mg)	Length (m)	Width (m)	Thickness (m)	Volume (m^3)	Force (N)	Force density ($N\ m^{-3}$)	Stroke (m)	Bend
1	1.4	2.7×10^{-4}	2.9×10^{-5}	1.0×10^{-5}	7.8×10^{-14}	1.2×10^{-5}	1.6×10^{8}	5.3×10^{-5}	X
2	2.4	2.6×10^{-4}	2.6×10^{-5}	5.4×10^{-6}	3.7×10^{-14}	2.1×10^{-5}	5.8×10^{8}	3.9×10^{-5}	O
3	5.4	1.9×10^{-4}	3.2×10^{-5}	4.0×10^{-6}	2.4×10^{-14}	4.8×10^{-5}	2.0×10^{9}	2.1×10^{-5}	O
4	9.1	2.9×10^{-4}	2.5×10^{-5}	7.0×10^{-6}	5.2×10^{-14}	8.0×10^{-5}	1.5×10^{9}	4.9×10^{-5}	O

Table S5. Crystal dimensions and bead-pushing-based work-density analysis of Y-phase single crystals during the Y→C transformation.

	Bead type	Bead radius (m)	Bead mass (mg)[a]	Length (m)	Width (m)	Thickness (m)	Volume (m^3)	Bead displacement (m)	Response time (ms)	Work density[b] (J/m^3)	Power density (W/m^3)
1	Zirconia	1.7×10^{-4}	0.12	2.0×10^{-4}	2.6×10^{-5}	5.2×10^{-6}	2.6×10^{-14}	1.8×10^{-4}	31	71	2299
2	Zirconia	1.8×10^{-4}	0.15	1.8×10^{-4}	5.2×10^{-5}	7.9×10^{-6}	7.5×10^{-14}	4.1×10^{-4}	32	168	5260
3	Zirconia	1.8×10^{-4}	0.15	2.3×10^{-4}	4.4×10^{-5}	9.8×10^{-6}	9.8×10^{-14}	5.8×10^{-4}	63	63	996
4	Zirconia	1.7×10^{-4}	0.12	2.2×10^{-4}	3.0×10^{-5}	4.8×10^{-6}	3.2×10^{-14}	2.4×10^{-4}	32	106	3304

[a] The bead mass was calculated from its radius, assuming spherical geometry and using ρ = 5.9 g cm^{-3} for zirconia beads.

[b] The work density was estimated from the bead kinetic energy normalized by the crystal volume.

Table S6. Single point TD-DFT results on monomers and tetramers for relevant electronic transitions: vertical transition energies, oscillator strength *f*, electronic description, with H (L) as the highest (lowest) (un)occupied molecular orbital.

	Transitions	Energy (eV)	λ (nm)	*f*	Electronic description	
F_M	S_0-S_1	3.50	354	1.959	H→L	88%
					H-1→L+1	10%
	S_0-S_2	4.11	302	0	H-1→L	45%
					H→L+1	52%
B_M	S_0-S_1	3.64	340	1.790	H→L	87%
					H-1→L+1	11%
	S_0-S_2	4.20	295	0	H-1→L	56%
					H→L+1	40%
Y_M	S_0-S_1	3.37	368	2.006	H→L	90%
					H-1→L+1	8%
	S_0-S_2	4.10	303	0.005	H-1→L	45%
					H→L+1	51%
B_T	S_0-S_1	3.50	355	0.011	H-1→L	15%
					H-1→L+1	25%
					H→L+3	21%
	S_0-S_2	3.53	351	0.061	H-3→L	18%
					H-3→L+1	10%
					H-2→L+2	39%
	S_0-S_4	3.78	328	5.343	H-3→L	10%
					H-2→L+2	22%
					H-1→L+1	10%
					H→L+3	11%
Y_T	S_0-S_1	3.02	410.3	0.002	H-2→L+3	10%
					H→L	24%
					H→L+1	15%
					H→L+2	34%
	S_0-S_2	3.05	406	0.002	H-1→L	46%
					H-1→L+1	23%
	S_0-S_4	3.42	363	4.169	H-1→L+1	21%
					H-1→L+2	16%
					H→L+3	33%
B_T (Y_M)	S_0-S_1	3.07	404	0.132	H-3→L	26%
					H-2→L+1	32%
					H-1→L+1	8%
	S_0-S_2	3.11	398	0.085	multi-conf.	
	S_0-S_5	3.49	355	4.102	multi-conf.	

10. Supplementary Figures

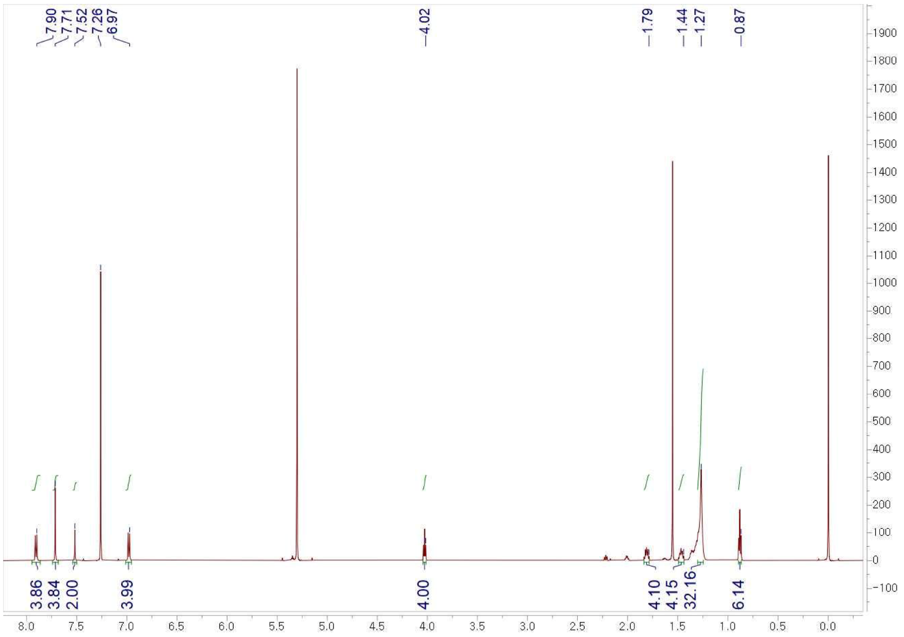

Figure S1. ^{1}H-NMR (600 MHz, $CDCl_3$). δ = 7.90 (d, 4H), 7.71 (s, 4H), 7.52 (s, 2H), 6.97 (d, 4H), 4.02 (t, 4H), 1.79 (m, 4H), 1.44 (m, 4H), 1.27 (m, 32H), 0.87 ppm (m, 6H).

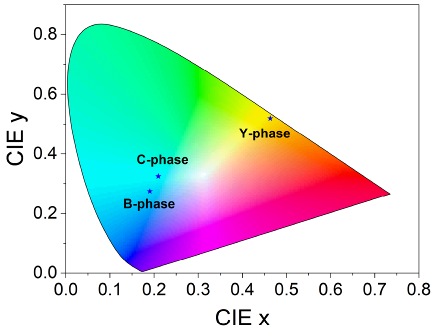

Figure S2. CIE 1931 chromaticity plot of the emission (x, y) coordinates calculated from the PL spectra of the B-, C-, and Y-phases.

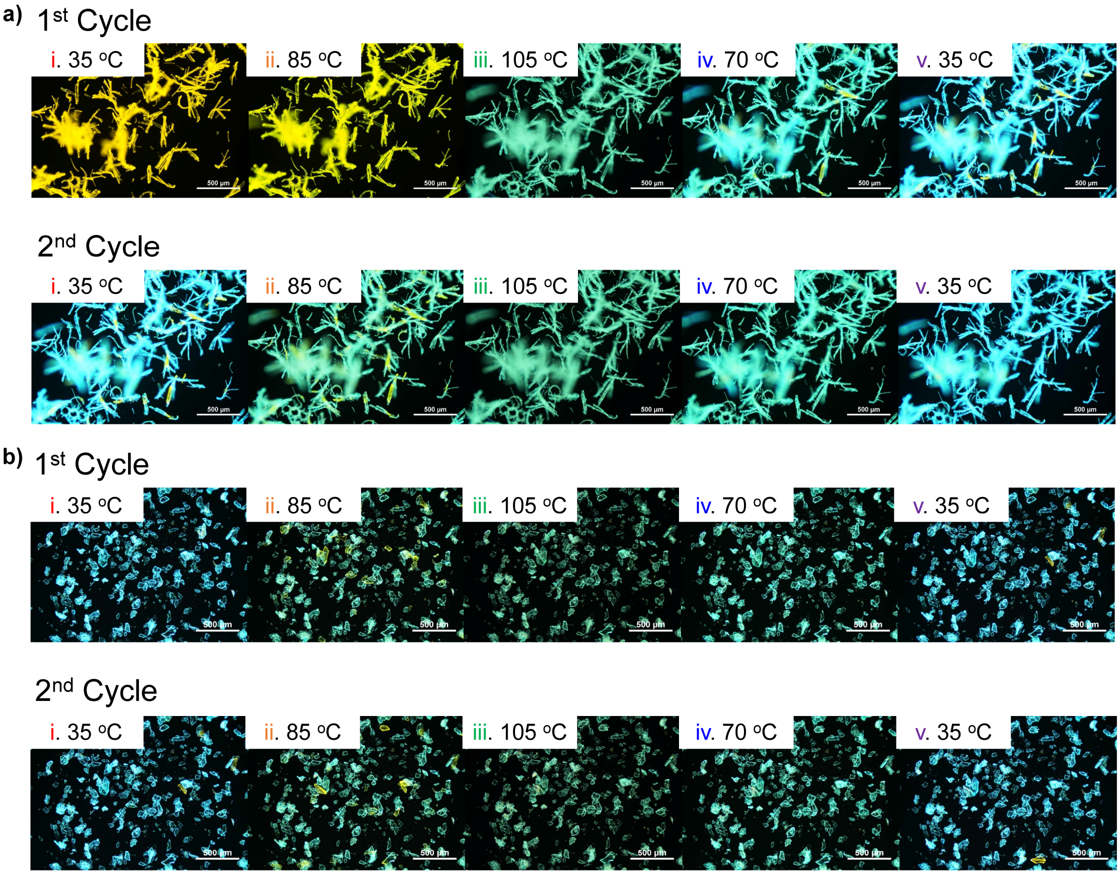

Figure S3. VT-FM images of αDDDCS crystalline powders acquired during the first two thermal cycles of five-cycle measurements starting from a, the as-prepared Y-phase and b, the B-phase. Full cycling movies are provided in **Movies S1** and **S2**.

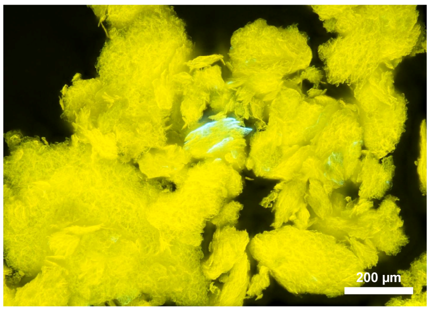

Figure S4. Fluorescence microscopy image of the as-prepared Y-phase powder showing trace B-phase impurity crystals, identified by blue emission within the predominantly yellow-emissive sample.

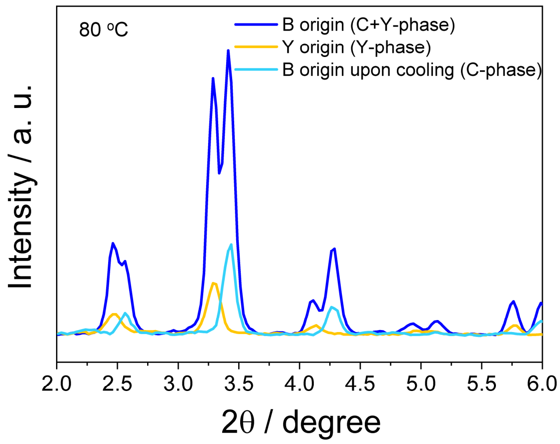

Figure S5. PXRD patterns in the lamellar reflection region at 80 °C for the mixed Y/C-phase pattern obtained from the B-origin sample during heating, the directly crystallized Y-phase collected during heating, and the C-phase reference obtained from the B-origin sample during cooling.

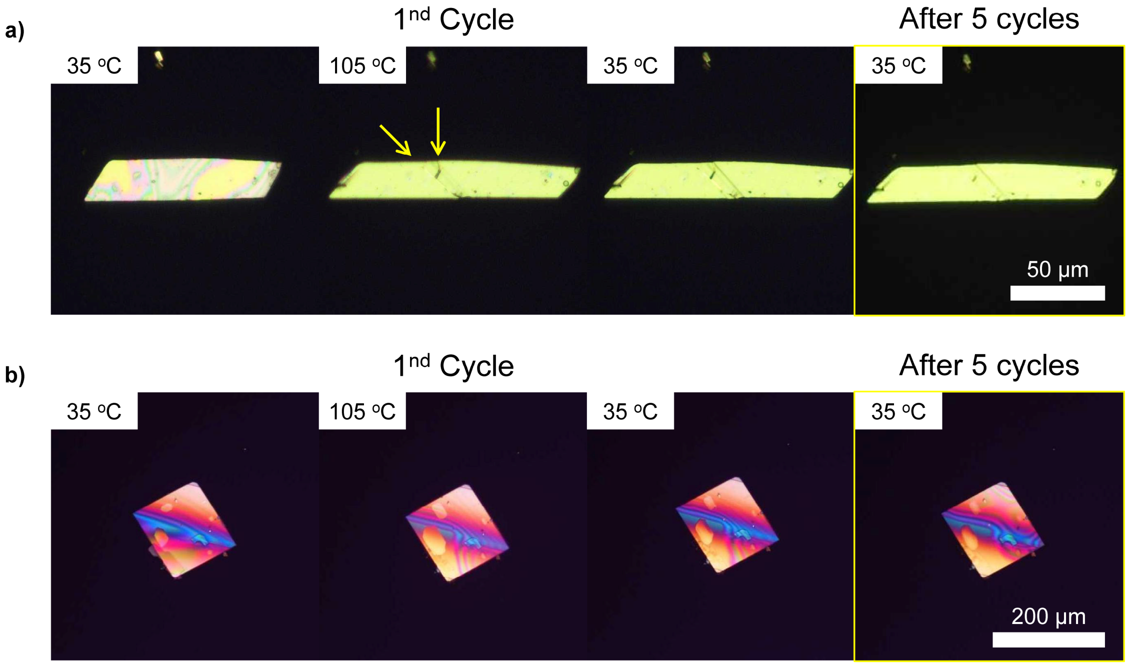

Figure S6. VT-CPOM images of αDDDCS single crystals during five thermal cycles starting from a, the Y-phase and b, the B-phase. The Y-phase crystal exhibits slight striation and microcrack formation after the initial Y→C transformation, as indicated by yellow arrows, whereas the B-phase crystal maintains its structural integrity during repeated B↔C cycling.

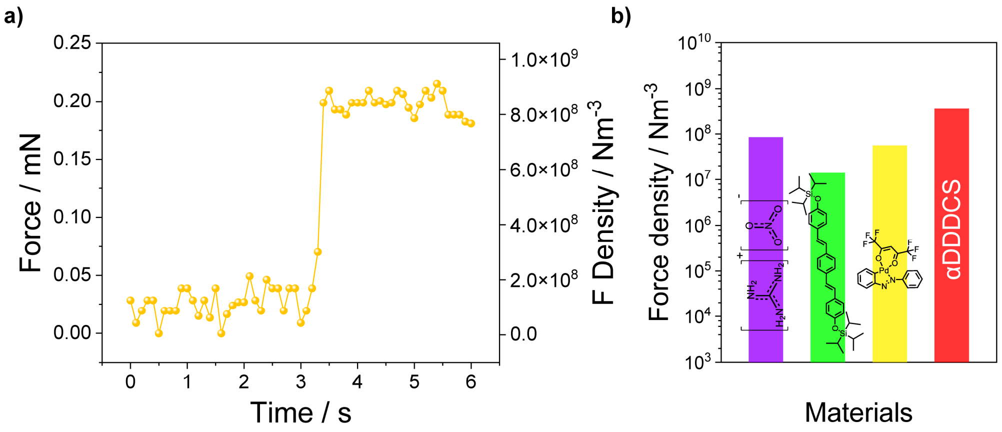

Figure S7. Force-density measurement of Y-phase single crystals during the Y→C transition using a miniature load cell. a, Representative force–time curve obtained during the Y→C transformation as the crystal pushes against the load cell. b, Comparison of the corresponding force density with those reported for representative dynamic molecular crystals.

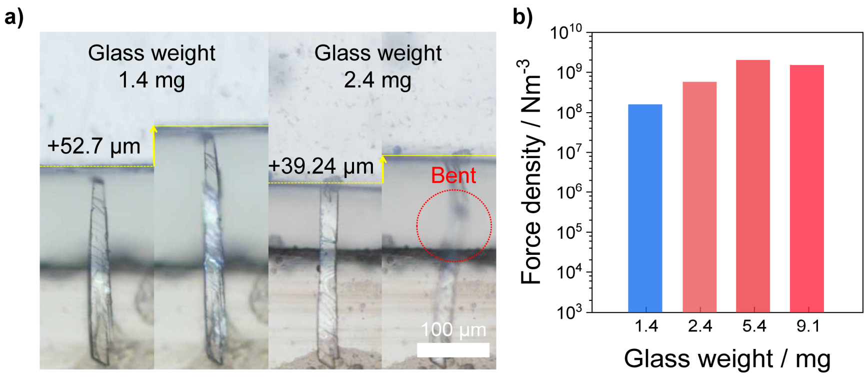

Figure S8. Glass-plate pushing experiments during the Y→C transformation of Y-phase crystals, performed to validate the force density estimated from load-cell measurements. a, Optical microscopy images showing displacement of glass plates with weights of 1.4 and 2.4 mg by transforming Y-phase crystals; bending deformation is observed under the higher load. b, Force-density values estimated from glass-plate pushing experiments with different glass weights. Based on these observations, the threshold for the onset of substantial bending is estimated to lie between 1.6 and 5.8 × 10^8 N m^{-3}.

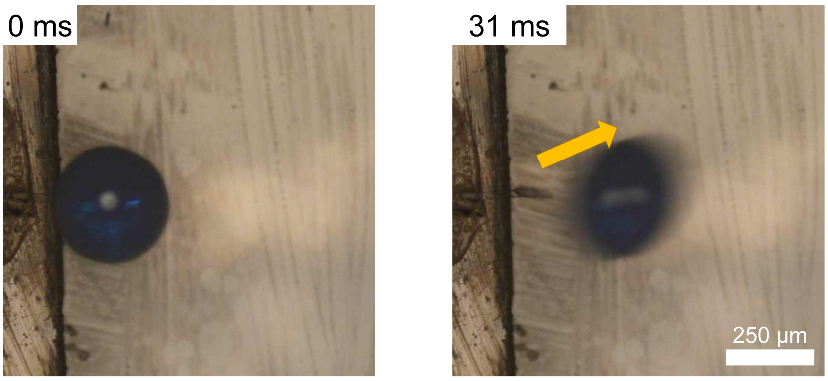

Figure S9. Bead-displacement experiments during the Y→C transformation of Y-phase crystals, performed to estimate the work density. Representative sequential optical microscopy images showing displacement of a zirconia bead by a Y-phase single crystal upon the Y→C transformation. The bead motion occurred within 31 ms, demonstrating measurable mechanical work output from the thermoelastic transition. The yellow arrow indicates the direction of bead displacement.

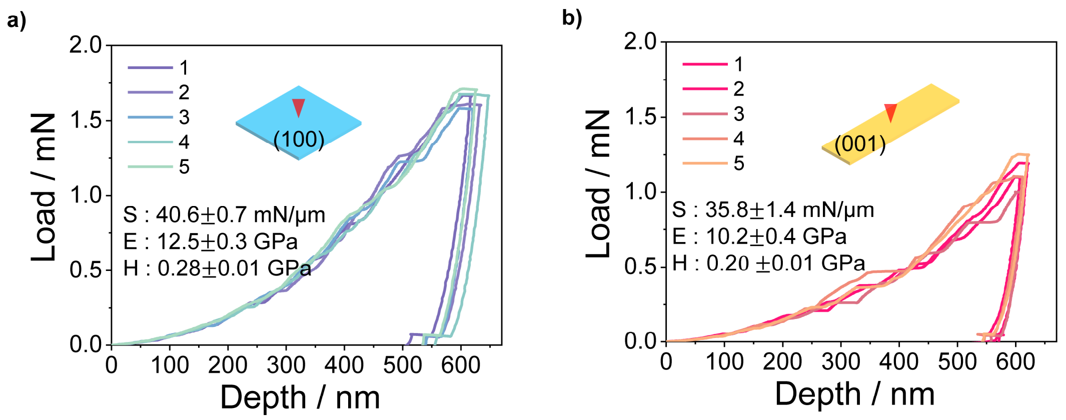

Figure S10. Nanoindentation measurements of B- and Y-phase single crystals. a, Load-displacement curves of the B-phase measured on the (100) face. b, Load-displacement curves of the Y-phase measured on the (001) face.

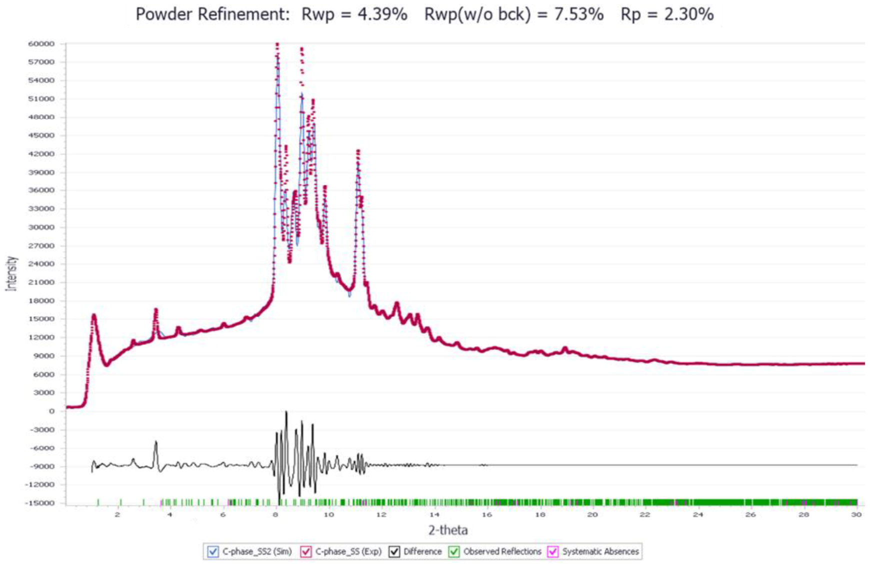

Figure S11. Pawley refinement of the VT-PXRD pattern collected for the high-temperature C-phase of αDDDCS at 378 K (B-phase origin). The refinement was performed using the Reflex Plus module in Materials Studio, yielding refined lattice parameters of a = 6.7369 Å, b = 47.654 Å, c = 7.0282 Å, β = 90.65°, and V = 2256.2 Å^3 with a monoclinic space group (P2/c) (R_{wp} = 4.39% and R_p = 2.30%). The refinement supports a lattice metric closely related to that of the B-phase, consistent with preservation of the μ-herringbone-type packing motif.

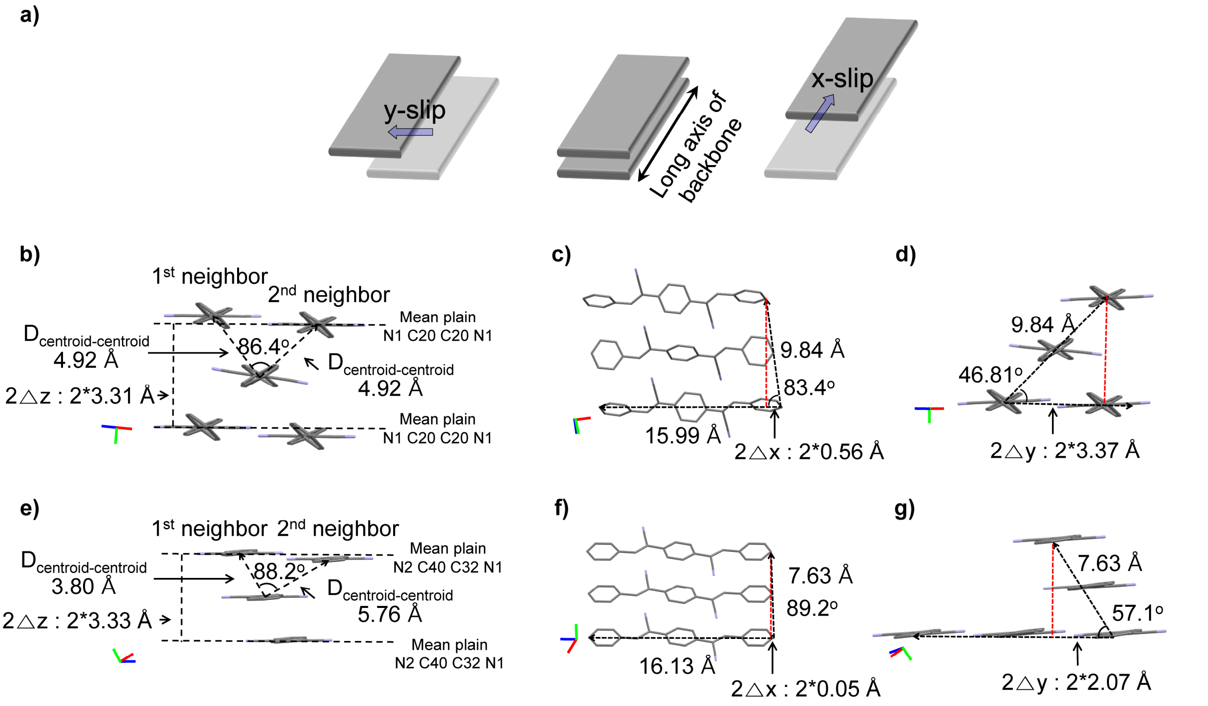

Figure S12. Average slips analysis from translationally equivalent molecules in αDDDCS crystal structures. a, Schematic definition of y-slip (along short axis of the backbone) and x-slip (along long axis of the backbone). b, z-slip ($\Delta z = 3.31$ Å) based on the mean planes of equivalent molecules and the center-to-center distance between neighboring molecules (1st and 2nd neighbors: 4.92 Å) in the B-phase. c, Molecular length (C_α-C_ω = 15.99 Å) of the DCS core measured between terminal carbon atoms (C13–C13) and determination of x-slip ($\Delta x = 0.56$ Å) in the B-phase. d, y-slip ($\Delta y = 3.37$ Å) in the B-phase. e, Definition of z-slip ($\Delta z = 3.33$ Å) between the mean planes of equivalent molecules and the center-to-center distance between neighboring molecules (1st: 3.80 Å, 2nd: 5.76 Å) in the Y-phase. f, Molecular length (Cα-Cω = 16.13 Å) of the DCS core measured between terminal carbon atoms (C25–C46) and determination of x-slip ($\Delta x = 0.05$ Å) in the Y-phase. g, y-slip ($\Delta y = 2.07$ Å) in the Y-phase.

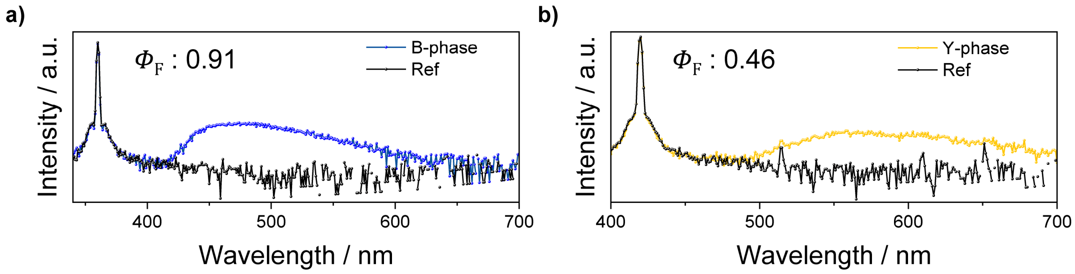

Figure S13. Quantum yields of αDDDCS polymorphs. a, B-phase (blue) with $\Phi_F = 0.91$; b, Y-phase (yellow) with $\Phi_F = 0.46$.

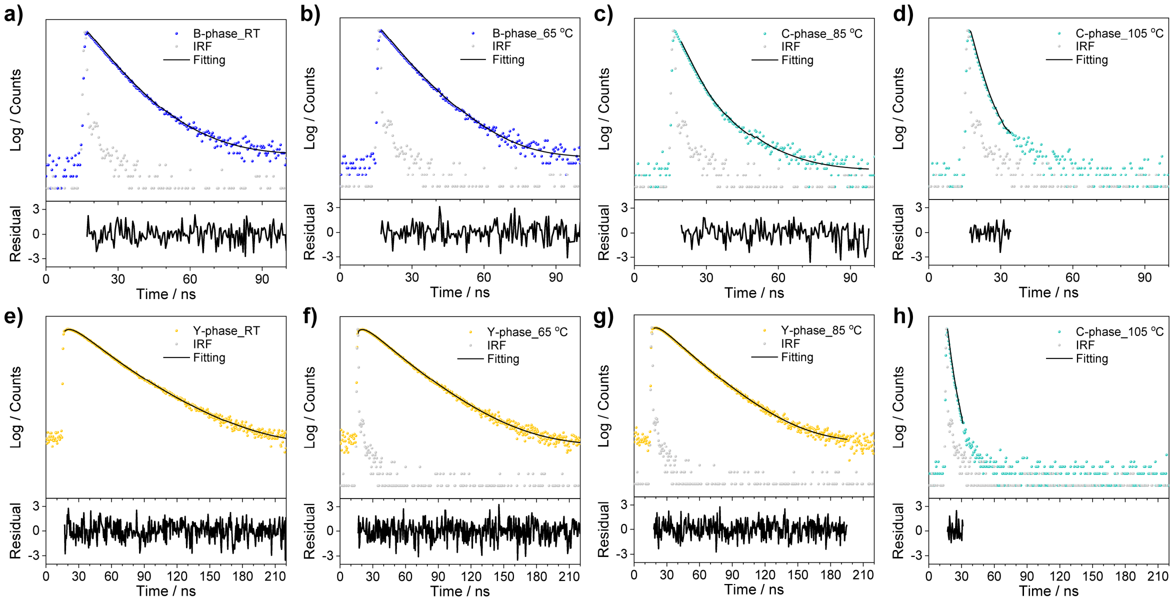

i)

Materials	Temp (℃)	A_1	τ_1 (ns)	A_2	τ_2 (ns)	A_3	τ_3 (ns)	f_1	f_2	f_3	χ	Average lifetime	
												$\tau_{amp}{}^{a}$ (ns)	$\tau_{int}{}^{b}$ (ns)
B	RT	0.93	5.51	0.07	12.41	-	-	0.85	0.15	-	1.07	5.96	6.45
B	65	0.86	5.26	0.14	10.36	-	-	0.76	0.24	-	1.13	5.94	6.44
$C_{from\ B}$	85	0.98	3.29	0.02	11.42	-	-	0.94	0.06	-	1.17	3.46	3.87
$C_{from\ B}$	105	0.94	1.71	0.06	3.78	-	-	0.88	0.12	-	0.97	1.83	1.96
Y	RT	-0.67	5.85	0.82	17.20	0.18	31.35	-	0.60	0.40	1.12	19.80	21.32
Y	65	-0.52	5.00	0.86	16.55	0.14	29.52	-	0.65	0.35	1.19	18.40	19.51
Y	85	-0.41	4.44	0.84	15.56	0.16	26.26	-	0.59	0.41	0.93	17.28	18.17
$C_{from\ Y}$	105	0.74	1.54	0.26	2.85	-	-	0.60	0.40	-	1.10	1.88	2.06

a) Amplitude-averaged lifetimes : $\tau_{amp} = \sum A_i\tau_i / \sum A_i$

b) Intensity-averaged lifetimes : $\tau_{int} = \sum A_i\tau_i^2 / \sum A_i\tau_i$

* The amplitude- and intensity-averaged lifetimes for Y-phase were calculated using τ_2 and τ_3.

Figure S14. Temperature-dependent TRPL decays and bi-exponential fitting for B-, Y-, and C-phases. a–d, TRPL decays of the B-phase at RT and 65 °C, and the C-phase at 85 and 105 °C. e–h, TRPL decays of the Y-phase at RT, 65, and 85 °C, and the C-phase at 105 °C. i, Fitting parameters of the TRPL decays for the B- and C-phases obtained by two-exponential fitting and for the Y-phase obtained by three-exponential fitting.

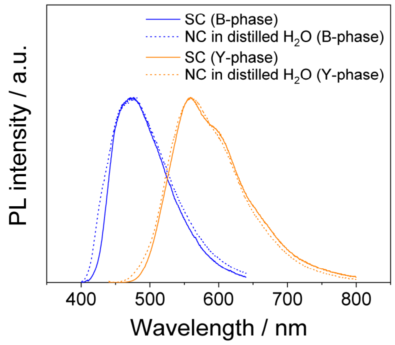

Figure S15. PL spectra of B- and Y-phase measured for single crystals and crystal suspensions in distilled H_2O.

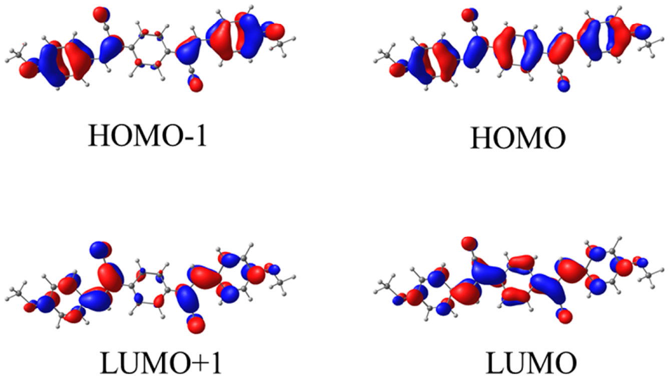

Figure S16. Molecular orbitals (MOs) of F_M, from DFT (HOMO = Highest occupied MO, LUMO = Lowest unoccupied MO).

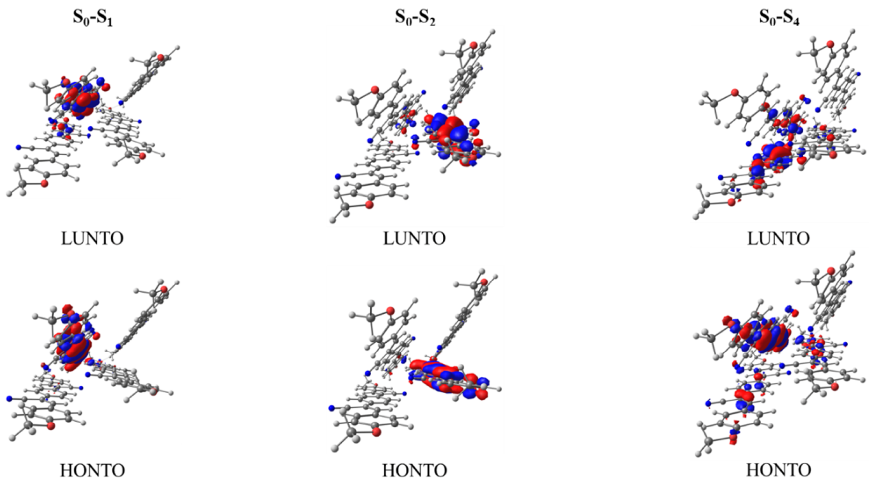

Figure S17. Natural transition orbitals (NTOs) of B_T, from TD-DFT (HONTO = Highest occupied NTO, LUNTO = Lowest unoccupied NTO).

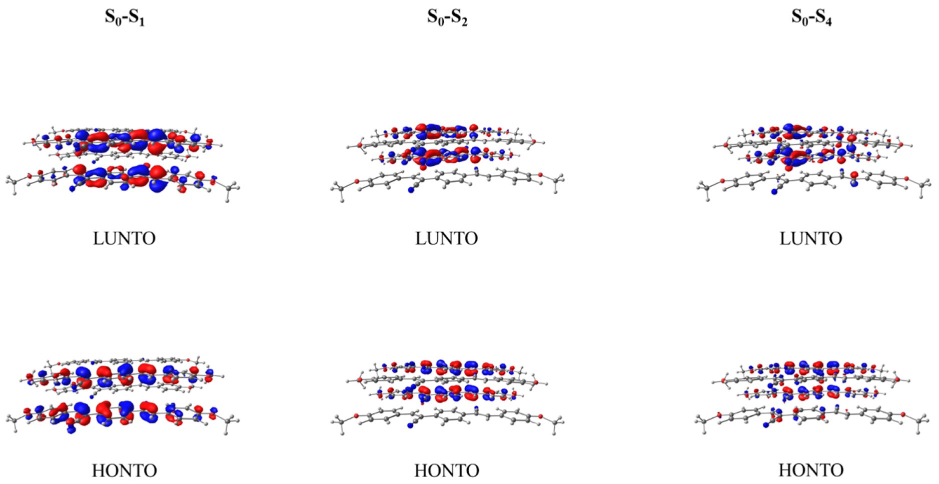

Figure S18. Natural transition orbitals (NTOs) of Y_T, from TD-DFT (HONTO = Highest occupied NTO, LUNTO = Lowest unoccupied NTO).

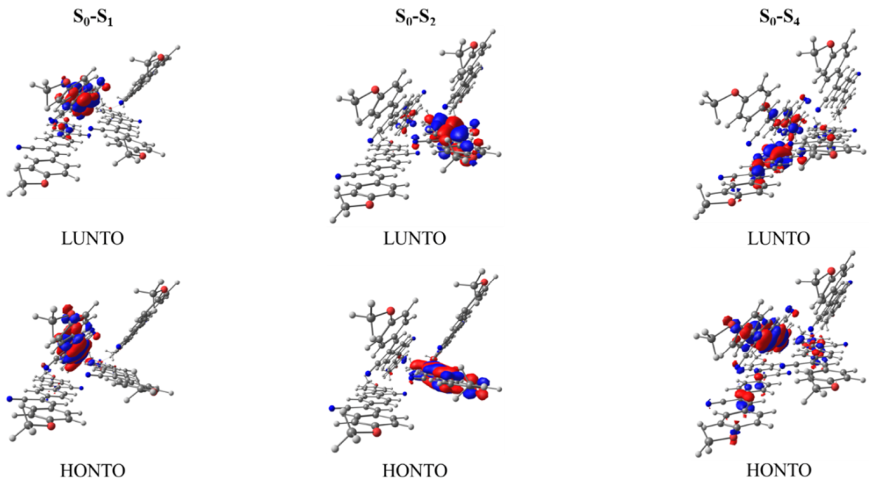

Figure S19. Natural transition orbitals (NTOs) of $B_T(Y_M)$ from TD-DFT (HONTO = Highest occupied NTO, LUNTO = Lowest unoccupied NTO).

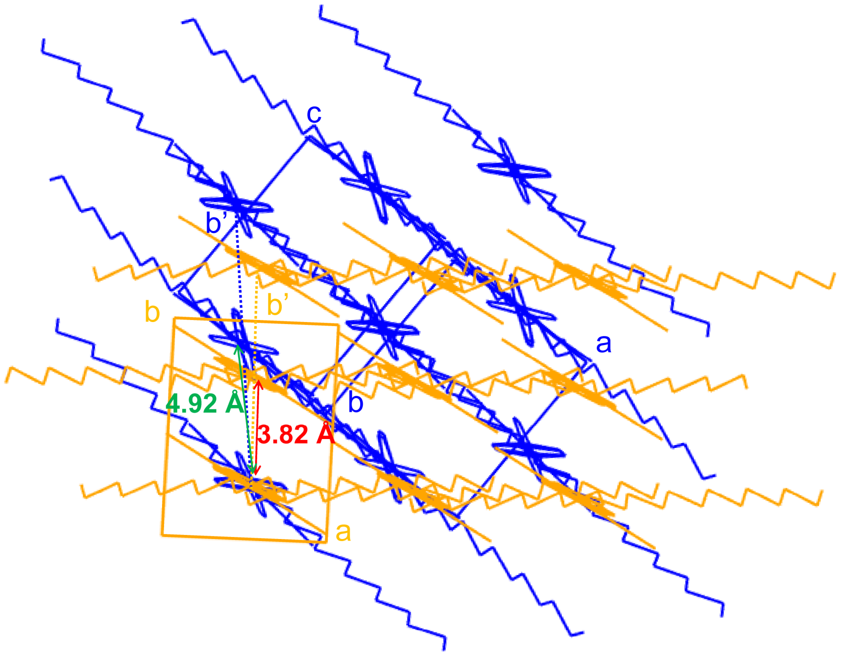

Figure S20. Supercell-based comparison of the Y- and B-phase crystal structures. The structural overlay shows the one-to-one molecular relationship and relative displacement along the stacking direction, explaining the large macroscopic deformation observed experimentally.

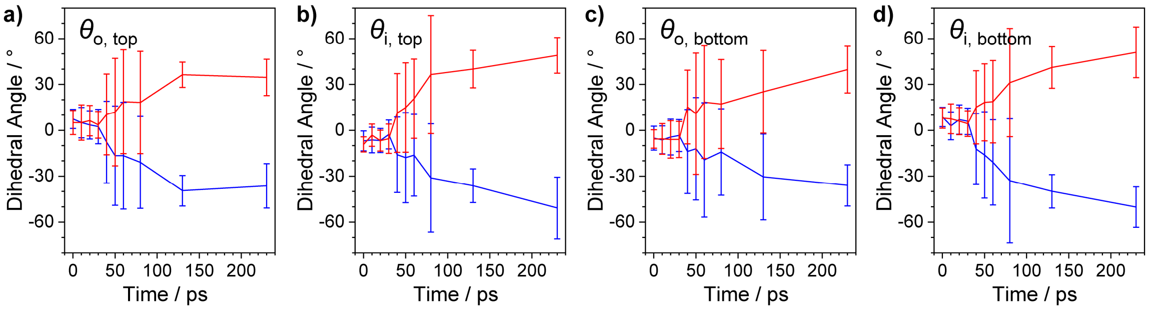

Figure S21. Time-dependent mean and standard deviation of the four backbone torsional angles. a, $\theta_{\text{o, top}}$, b, $\theta_{\text{i, top}}$, c, $\theta_{\text{o, bottom}}$, and d, $\theta_{\text{i, bottom}}$ for the positively (red) and negatively (blue) rotated molecular groups.

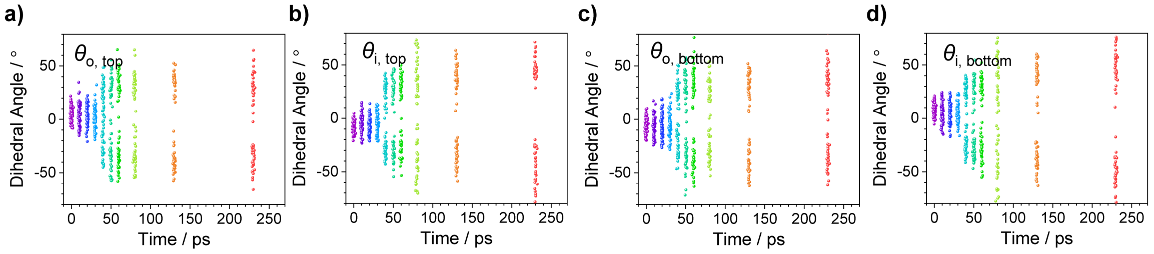

e)

		Average dihedral angles (°)									
		0.1 ps	10 ps	20 ps	30 ps	40 ps	50 ps	60 ps	80 ps	130 ps	230 ps
$\theta_{o,\ top}$	1st layer	5.1±7.9	5.1±11.5	6.3±9.9	3.6±8.7	10.4±26.4	11.9±35.1	18.7±34	18.2±33.5	36.4±8.3	34.6±11.9
	2nd layer	7.4±6.2	5.1±9.9	3.5±9.3	2.5±11.3	-7.8±26.8	-16.6±32.4	-16.6±34.9	-21.0±30.0	-39.5±9.9	-36.3±14.5
$\theta_{i,\ top}$	1st layer	-9.2±4.9	-3.2±7.3	-6.8±6.9	-5.5±9.6	11.4±25.7	14.9±29.2	207±25.9	36.5±38.6	40.1±12.2	49.0±11.7
	2nd layer	-6.9±6.5	-6.3±8.3	-6.7±7.8	-2.7±9.4	-15.9±24.6	-17.9±29.4	-16.3±26.7	-31.1±35.4	-36.3±11.0	-50.8±20.1
$\theta_{o,\ bottom}$	1st layer	-5.7±6.1	-5.6±10.0	-6.0±12.2	-6.0±11.8	15.2±24.1	10.9±39.7	18.4±36.9	17.2±29.1	25.2±27.0	39.7±15.3
	2nd layer	-5.1±7.8	-6.2±9.2	-4.4±11.7	-3.4±10.4	-13.8±27.5	-12.1±33.5	-19.3±37.4	-14.2±28.1	-30.4±28.1	-36.0±13.5
$\theta_{i,\ bottom}$	1st layer	8.0±6.6	7.5±9.7	6.0±8.8	4.4±8.4	15.1±24.0	18.3±25.2	18.7±27.0	31.2±35.4	41.1±13.6	51.0±16.6
	2nd layer	8.7±6.3	2.9±9.0	7.0±9.6	5.9±8.6	-12.3±23.2	-16.1±28.2	-20.9±27.9	-33.0±40.6	-39.9±10.9	-50.2±13.2

Figure S22. Time evolution of the four backbone torsional angles from MD simulations. a–d, Distributions of (a) $\theta_{o,\ top}$, (b) $\theta_{i,\ top}$, (c) $\theta_{o,\ bottom}$, and (d) $\theta_{i,\ bottom}$ for all molecules in the two layers. e, Corresponding average dihedral angles for each torsion in the first and second layers.

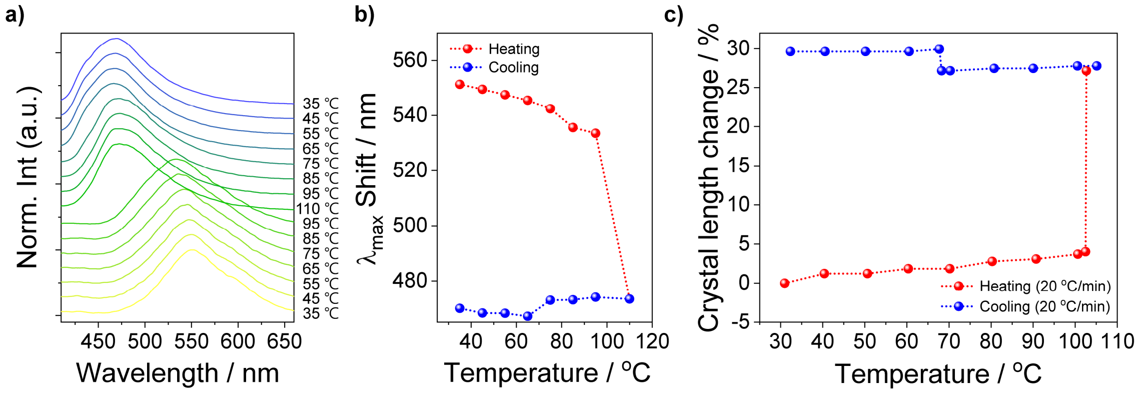

Figure S23. Temperature-dependent photoluminescence and crystal length change during heating and cooling. a, Normalized PL spectra at variable temperature. b,c, Temperature-dependent evolution of (b) the emission maximum (λ_{max}) and (c) relative crystal length change.

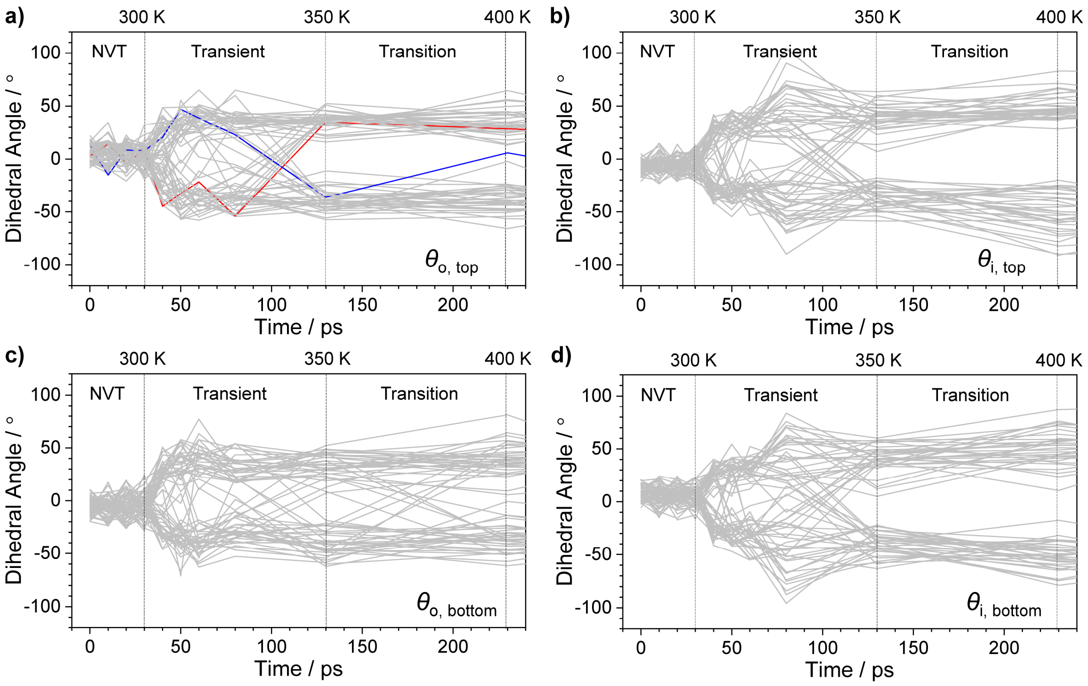

Figure S24. Trajectories of the four backbone torsional angles from MD simulations. a–d, Individual trajectories of (a) $\theta_{o,\ top}$, (b) $\theta_{i,\ top}$, (c) $\theta_{o,\ bottom}$, and (d) $\theta_{i,\ bottom}$ for all molecules, separated into NVT (300 K), transient (350 K), and transition (400 K) regimes as indicated.

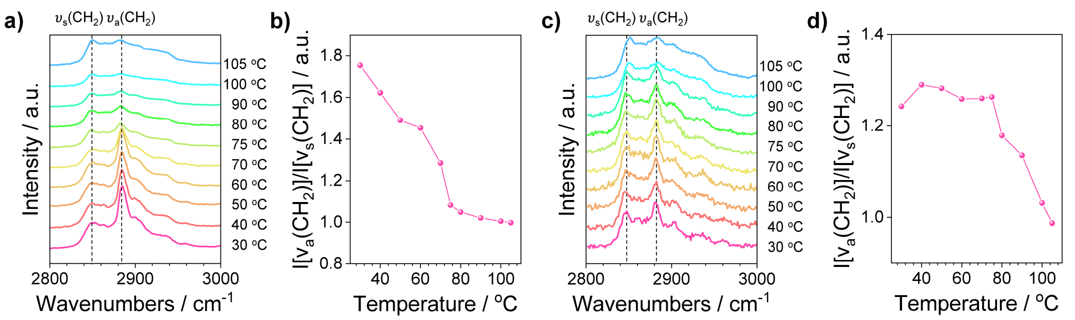

Figure S25. VT-Raman analysis of alkyl-chain disordering during the phase transitions of αDDDCS. a, b, B→C transition measured using a 514 nm excitation laser. c, d, Y→C transition measured using a 785 nm excitation laser. The decrease in the $I[\nu_a(CH_2)]/I[\nu_s(CH_2)]$ ratio indicates increased rotational and conformational disorder of the alkyl chains in the C-phase.

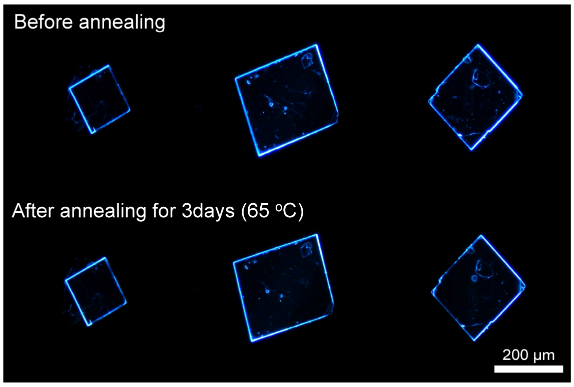

Figure S26. Fluorescence microscopy images of B-phase crystals a, before and b, after thermal annealing at 65 °C in a convection oven for 3 days. No observable B→Y conversion is detected, indicating that the B→Y transition is not readily triggered by thermal activation alone under these conditions.

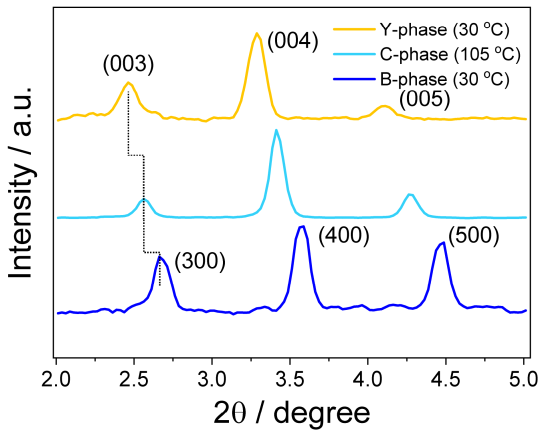

Figure S27. Powder X-ray diffraction patterns of αDDDCS for crystalline powders in the B-phase at 30 °C, C-phase at 105 °C and Y-phase at 30 °C.

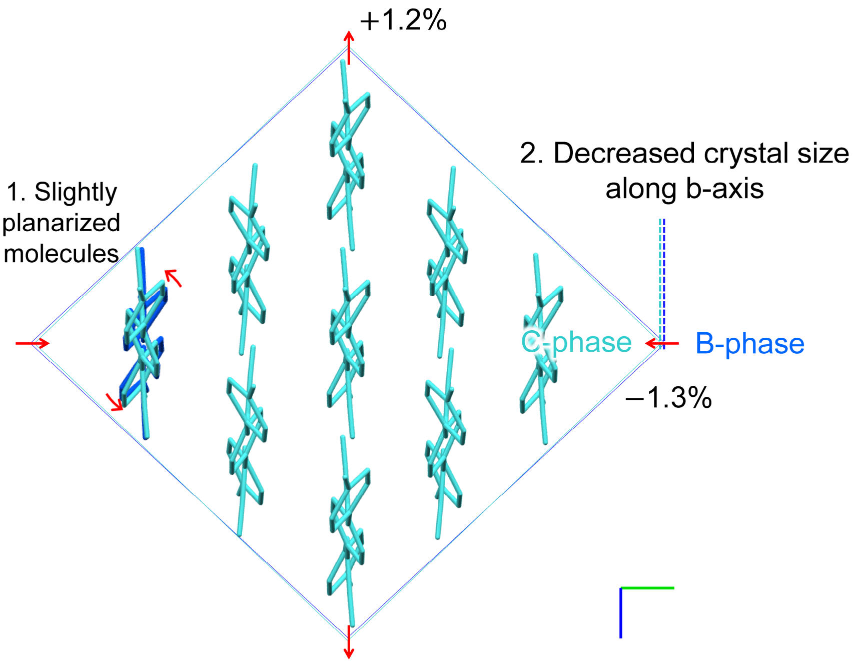

Figure S28. Schematic B-/C-phase overlay model constructed from the observed crystal dimensional changes during the B→C transition. The model visualizes the modest nature of the transformation, including slight contraction along the B-phase *b*-axis direction and limited molecular planarization.

11. Captions for Supplementary Movies

Movie S1. Five thermal cycles of Y-phase crystalline powder monitored by VT-FM during repeated heating–cooling cycles.

Movie S2. Five thermal cycles of B-phase crystalline powder monitored by VT-FM during repeated heating–cooling cycles.

Movie S3. Y-to-C phase transition of the single crystal observed by VT-FM during heating and cooling.

Movie S4. Y-to-C phase transition of the single crystal observed by VT-POM during heating and cooling.

Movie S5. B-to-C phase transition of the single crystal observed by VT-FM during heating and cooling.

Movie S6. B-to-C phase transition of the single crystal observed by VT-POM during heating and cooling.

Movie S7. Y-to-C phase transitions of the single crystal attached to a substrate via a thin adhesion layer.

Movie S8. B-to-C & -Y phase transitions of the single crystal attached to a substrate via a thin adhesion layer.

Movie S9. Glass-pushing experiment of a Y-phase single crystal during the Y→C transition: a glass weight of 1.4 mg and a crystal volume of 7.8×10^{-14} m^3.

Movie S10. Glass-pushing experiment of a Y-phase single crystal during the Y→C transition: a glass weight of 2.4 mg and a crystal volume of 3.7×10^{-14} m^3.

Movie S11. Bead-displacement experiment of a Y-phase single crystal during the Y→C transition: a zirconia bead weight of 0.12 mg and a crystal volume of 2.6×10^{-14} m^3.

Movie S12. Mechanosalient transition at the variable temperature (from 55 to 90 ºC) by mechanical probing the single crystals.

Movie S13. Shear along the [010] direction of the B phase at 73 °C.

Movie S14. Shear along the arbitrary direction of the B phase at 73 °C.

12. References

1. Kim, H. J.; Gierschner, J.; Park, S. Y., Tricolor fluorescence switching in a single component mechanochromic molecular material. *J. Mater. Chem. C* **2020,** *8*, 7417-7421.

2. Duan, Y.; Semin, S.; Tinnemans, P.; Cuppen, H.; Xu, J.; Rasing, T., Robust thermoelastic microactuator based on an organic molecular crystal. *Nat. Commun.* **2019,** *10*, 4573.

3. Shin, J. W.; Eom, K.; Moon, D., BL2D-SMC, the supramolecular crystallography beamline at the Pohang Light Source II, Korea. *J. Synchrotron Radiat.* **2016,** *23*, 369-373.

4. Otwinowski, Z.; Minor, W., Processing of X-ray diffraction data collected in oscillation mode. *Methods Enzymol.* **1997,** *276*, 307-326.

5. Sheldrick, G. M., SHELXT - integrated space-group and crystal-structure determination. *Acta Crystallogr. A* **2015,** *71*, 3-8.

6. Sheldrick, G. M., Crystal structure refinement with SHELXL. *Acta Crystallogr. C* **2015,** *71*, 3-8.

7. Hammersley, A. Fit2D program, ESRF; 6 Rue Jules Horowitz, BP 220 38043, Grenoble CEDEX 9, France.

8. Neumann, M. A., X-Cell: a novel indexing algorithm for routine tasks and difficult cases. *J. Appl. Crystallogr.* **2003,** *36*, 356-365.

9. Pawley, G. S., Unit-cell refinement from powder diffraction scans. *J. Appl. Crystallogr.* **1981,** *14*, 357-361.

10. Bérar, J.-F.; Baldinozzi, G., Modeling of line-shape asymmetry in powder diffraction. *J. Appl. Crystallogr.* **1993,** *26*, 128-129.

11. Frisch, M. J.; Trucks, G. W.; Schlegel, H. B.; Scuseria, G. E.; Robb, M. A.; Cheeseman, J. R.; Scalmani, G.; Barone, V.; Petersson, G. A.; Nakatsuji, H.; Li, X.; Caricato, M.; Marenich, A. V.; Bloino, J.; Janesko, B. G.; Gomperts, R.; Mennucci, B.; Hratchian, H. P.; Ortiz, J. V.; Izmaylov, A. F.; Sonnenberg, J. L.; Williams-Young, D.; Ding, F.; Lipparini, F.; Egidi, F.; Goings, J.; Peng, B.; Petrone, A.; Henderson, T.; Ranasinghe, D.; Zakrzewski, V. G.; Gao, J.; Rega, N.; Zheng, G.; Liang, W.; Hada, M.; Ehara, M.; Toyota, K.; Fukuda, R.; Hasegawa, J.; Ishida, M.; Nakajima, T.; Honda, Y.; Kitao, O.; Nakai, H.; Vreven, T.; Throssell, K.; Montgomery, J. A., Jr.; Peralta, J. E.; Ogliaro, F.; Bearpark, M. J.; Heyd, J. J.; Brothers, E. N.; Kudin, K. N.; Staroverov, V. N.; Keith, T. A.; Kobayashi, R.; Normand, J.; Raghavachari, K.; Rendell, A. P.; Burant, J. C.; Iyengar, S. S.; Tomasi, J.; Cossi, M.; Millam, J. M.; Klene, M.; Adamo, C.; Cammi, R.; Ochterski, J. W.; Martin, R. L.; Morokuma, K.; Farkas, O.; Foresman, J. B.; Fox, D. J., Gaussian 16, Revision C.01. *Gaussian, Inc.: Wallingford, CT,* **2016**.

12. Tkatchenko, A.; Scheffler, M., Accurate molecular van der Waals interactions from ground-state electron density and free-atom reference data. *Phys. Rev. Lett.* **2009,** *102*, 073005.

13. Abozeed, A.; Sayed, M.; Younis, O.; Tolba, M. S.; Hassanien, R.; El-Dean, A. M. K.; Ibrahim, S. M.; Salah, A.; Shakir, A.; El-Sayed, R.; El-Ossaily, Y. A.; Al-Hossainy, A. F., Characterization and optical behavior of a new indole Schiff base using experimental data and TD-DFT/DMOl computations. *Opt. Mater.* **2022,** *131*, 112594.

14. Plimpton, S., Fast Parallel Algorithms for Short-Range Molecular-Dynamics. *J. Comput. Phys.* **1995,** *117*, 1-19.

15. Duke, R.; Giese, T.; Gohlke, H.; Goetz, A.; Homeyer, N.; Izadi, S.; Janowski, P.; Kaus, J.; Kovalenko, A.; Lee, T., AMBER 2016; University of California: San Francisco. **2016**.

16. Neese, F., The ORCA program system. *Wiley Interdisciplinary Reviews-Computational Molecular Science* **2012,** *2*, 73-78.

17. Neese, F., Software Update: The ORCA Program System-Version 6.0. *Wiley Interdisciplinary Reviews-Computational Molecular Science* **2025,** *15*, e70019.

18. Ewald, P. P., Die Berechnung optischer und elektrostatischer Gitterpotentiale. *Ann. Phys.* **1921,** *369*, 253-287.

19. de Leeuw, S. W.; Perram, J. W.; Smith, E. R., Simulation of electrostatic systems in periodic boundary conditions. I. Lattice sums and dielectric constants. *Proc. R. Soc. Lond. A* **1980,** *373*, 27-56.

20. Karothu, D. P.; Weston, J.; Desta, I. T.; Naumov, P., Shape-Memory and Self-Healing Effects in Mechanosalient Molecular Crystals. *J. Am. Chem. Soc.* **2016,** *138*, 13298-13306.

21. Karothu, D. P.; Ferreira, R.; Dushaq, G.; Ahmed, E.; Catalano, L.; Halabi, J. M.; Alhaddad, Z.; Tahir, I.; Li, L.; Mohamed, S.; Rasras, M.; Naumov, P., Exceptionally high work density of a ferroelectric dynamic organic crystal around room temperature. *Nat. Commun.* **2022,** *13*, 2823.

22. Hwang, K.; Sin, G.; Jang, M.; Choi, Y. M.; Moon, D.; Park, H.; Park, S. K., Amplifying Colossal Thermal Expansion of a Martensitic Molecular Crystal through Interlayer Shear-Induced Side-Chain Liberation. *Angew. Chem. Int. Ed.* **2025,** *64*, e202415821.

23. Khalil, A.; Karothu, D. P.; Naumov, P., Direct quantification of rapid and efficient single-stroke actuation by a martensitic transition in a thermosalient crystal. *J. Am. Chem. Soc.* **2019,** *141*, 3371-3375.

24. Gierschner, J.; Huang, Y. S.; Van Averbeke, B.; Cornil, J.; Friend, R. H.; Beljonne, D., Excitonic versus electronic couplings in molecular assemblies: The importance of non-nearest neighbor interactions. *J. Chem. Phys.* **2009,** *130*, 044105.

25. Shi, J. Q.; Suarez, L. E. A.; Yoon, S. J.; Varghese, S.; Serpa, C.; Park, S. Y.; Lüer, L.; Roca-Sanjuán, D.; Milián-Medina, B.; Gierschner, J., Solid State Luminescence Enhancement in π-Conjugated Materials: Unraveling the Mechanism beyond the Framework of AIE/AIEE. *J. Phys. Chem. C* **2017,** *121*, 23166-23183.

26. Shi, J. Q.; Izquierdo, M. A.; Oh, S.; Park, S. Y.; Milián-Medina, B.; Roca-Sanjuán, D.; Gierschner, J., Inverted energy gap law for the nonradiative decay in fluorescent floppy molecules: larger fluorescence quantum yields for smaller energy gaps. *Org. Chem. Front.* **2019,** *6*, 1948-1954.

27. Gierschner, J.; Shi, J. Q.; Milián-Medina, B.; Roca-Sanjuán, D.; Varghese, S.; Park, S. Y., Luminescence in Crystalline Organic Materials: From Molecules to Molecular Solids. *Adv. Optical Mater.* **2021,** *9*, 2002251.

28. Gierschner, J.; Lüer, L.; Milián-Medina, B.; Oelkrug, D.; Egelhaaf, H.-J., Highly Emissive H-Aggregates or Aggregation-Induced Emission Quenching? The Photophysics of All-Trans para-Distyrylbenzene. *J. Phys. Chem. Lett.* **2013,** *4*, 2686-2697.

29. Strickler, S.; Berg, R. A., Relationship between absorption intensity and fluorescence lifetime of molecules. *J. Chem. Phys.* **1962,** *37*, 814-822.

30. Hestand, N. J.; Spano, F. C., Expanded Theory of H- and J-Molecular Aggregates: The Effects of Vibronic Coupling and Intermolecular Charge Transfer. *Chem. Rev.* **2018,** *118*, 7069-7163.

31. Yoon, S. J.; Varghese, S.; Park, S. K.; Wannemacher, R.; Gierschner, J.; Park, S. Y., Color-Tuned, Highly Emissive Dicyanodistyrylbenzene Single Crystals: Manipulating Intermolecular Stacking Interactions for Spontaneous and Stimulated Emission Characteristics. *Adv. Optical Mater.* **2013,** *1*, 232-237.

32. Birks, J.; Kazzaz, A.; King, T., 'Excimer'fluorescence-IX. Lifetime studies of pyrene crystals. *Proc. R. Soc. Lond. A* **1966,** *291*, 556-569.

33. Seyfang, R.; Port, H.; Fischer, P.; Wolf, H., Picosecond study on excimer formation in pyrene crystals: Part III: Complete analysis of the high temperature phase between 5 and 300 K. *J. Lumin.* **1992,** *51*, 197-208.

34. Li, L.; Commins, P.; Al-Handawi, M. B.; Karothu, D. P.; Halabi, J. M.; Schramm, S.; Weston, J.; Rezgui, R.; Naumov, P., Martensitic organic crystals as soft actuators. *Chem. Sci.* **2019,** *10*, 7327-7332.

35. Borbone, F.; Tuzi, A.; Carella, A.; Marabello, D.; Oscurato, S. L.; Lettieri, S.; Maddalena, P.; Centore, R., High-Temperature Reversible Martensitic Transition in an Excited-State Intramolecular Proton Transfer Fluorophore. *Cryst. Growth Des.* **2019,** *19*, 6519-6526.

36. Davies, D. W.; Seo, B.; Park, S. K.; Shiring, S. B.; Chung, H.; Kafle, P.; Yuan, D.; Strzalka, J. W.; Weber, R.; Zhu, X.; Savoie, B. M.; Diao, Y., Unraveling two distinct polymorph transition mechanisms in one n-type single crystal for dynamic electronics. *Nat. Commun.* **2023,** *14*, 1304.

37. Lin, J. W.; Guo, Z. X.; Zhang, K. K.; Zhao, P.; Wu, S. G.; Xu, J.; Gong, J. B.; Bao, Y., Mechanical Motion and Modulation of Thermal-Actuation Properties in a Robust Organic Molecular Crystal Actuator. *Adv. Funct. Mater.* **2022,** *32*, 2203004.

38. Panda, M. K.; Runcevski, T.; Sahoo, S. C.; Belik, A. A.; Nath, N. K.; Dinnebier, R. E.; Naumov, P., Colossal positive and negative thermal expansion and thermosalient effect in a pentamorphic organometallic martensite. *Nat. Commun.* **2014,** *5*, 4811.

39. Nath, N. K.; Severa, L.; Kunetskiy, R. A.; Cisarova, I.; Fulem, M.; Ruzicka, K.; Koval, D.; Kasicka, V.; Teply, F.; Naumov, P., Single-Crystal-to-Single-Crystal Transition in an Enantiopure [7]Helquat Salt: The First Observation of a Reversible Phase Transition in a Helicene-Like Compound. *Chem. Eur. J.* **2015,** *21*, 13508-13512.

40. Wang, Z.; Cheng, P.; Han, W.; Shi, R.; Xu, J.; Zheng, Y.; Xu, J.; Bu, X. H., Thermoelastic twisting-assisted crystal jumping based on a self-healing molecular crystal. *Proc. Natl. Acad. Sci. U.S.A.* **2025,** *122*, e2417901122.

41. Su, S. Q.; Kamachi, T.; Yao, Z. S.; Huang, Y. G.; Shiota, Y.; Yoshizawa, K.; Azuma, N.; Miyazaki, Y.; Nakano, M.; Maruta, G.; Takeda, S.; Kang, S.; Kanegawa, S.; Sato, O., Assembling an alkyl rotor to access abrupt and reversible crystalline deformation of a cobalt(II) complex. *Nat. Commun.* **2015,** *6*, 8810.

42. Park, S. K.; Sun, H.; Chung, H.; Patel, B. B.; Zhang, F.; Davies, D. W.; Woods, T. J.; Zhao, K.; Diao, Y., Super- and Ferroelastic Organic Semiconductors for Ultraflexible Single-Crystal Electronics. *Angew. Chem. Int. Ed.* **2020,** *59*, 13004-13012.

43. Meng, J.; Su, Y.; Zhu, H.; Zhang, J.; Cai, T., Dynamic molecular cocrystals with alkyl chain dependent thermosalient phase transitions. *Adv. Sci.* **2025,** *12*, 2502692.

44. Parisi, E.; Santagata, E.; Kula, P.; Herman, J.; Gupta, S.; Simone, E.; Zarrella, S.; Korter, T. M.; Centore, R., Mechanical Transitions in Crystals: The Low-Temperature Thermosalient Transition of a Mesogenic Polyphenyl. *J. Am. Chem. Soc.* **2025,** *147*, 14731-14738.

45. Uddin, M. A.; Martin, R.; Gamez-Valenzuela, S.; Echeverri, M.; Ruiz Delgado, M. C.; Gutierrez Puebla, E.; Monge, A.; Gomez-Lor, B., Giant Thermosalient Effect in a Molecular Single Crystal: Dynamic Transformations and Mechanistic Insights. *J. Am. Chem. Soc.* **2024,** *146*, 27690-27700.

46. Chung, H.; Dudenko, D.; Zhang, F.; D'Avino, G.; Ruzie, C.; Richard, A.; Schweicher, G.; Cornil, J.; Beljonne, D.; Geerts, Y.; Diao, Y., Rotator side chains trigger cooperative transition for shape and function memory effect in organic semiconductors. *Nat. Commun.* **2018,** *9*, 278.

47. Feng, C.; Seki, T.; Sakamoto, S.; Sasaki, T.; Takamizawa, S.; Ito, H., Mechanical deformation and multiple thermal restoration of organic crystals: reversible multi-stage shape-changing effect with luminescence-color changes. *Chem. Sci.* **2022,** *13*, 9544-9551.

48. Manoharan, D.; Ranjan, S.; Emmerling, F.; Bhattacharya, B.; Takamizawa, S.; Ghosh, S., Elasto-plastic behaviour with reversible thermosalient expansion in acrylonitrile-based organic crystals. *J. Mater. Chem. C* **2024,** *12*, 2515-2525.

49. Meng, J.; Su, Y.; Zhu, H.; Cai, T., Shape memory and self-healing in a molecular crystal with inverse temperature symmetry breaking. *Chem. Sci.* **2024,** *15*, 5738-5745.

50. Yao, Z. S.; Mito, M.; Kamachi, T.; Shiota, Y.; Yoshizawa, K.; Azuma, N.; Miyazaki, Y.; Takahashi, K.; Zhang, K.; Nakanishi, T.; Kang, S.; Kanegawa, S.; Sato, O., Molecular motor-driven abrupt anisotropic shape change in a single crystal of a Ni complex. *Nat. Chem.* **2014,** *6*, 1079-1083.

51. Tamboli, M. I.; Karothu, D. P.; Shashidhar, M. S.; Gonnade, R. G.; Naumov, P., Effect of Crystal Packing on the Thermosalient Effect of the Pincer-Type Diester Naphthalene-2,3-diyl-bis(4-fluorobenzoate): A New Class II Thermosalient Solid. *Chem. Eur. J.* **2018,** *24*, 4133-4139.

52. Dharmarwardana, M.; Pakhira, S.; Welch, R. P.; Caicedo-Narvaez, C.; Luzuriaga, M. A.; Arimilli, B. S.; McCandless, G. T.; Fahimi, B.; Mendoza-Cortes, J. L.; Gassensmith, J. J., Rapidly Reversible Organic Crystalline Switch for Conversion of Heat into Mechanical Energy. *J. Am. Chem. Soc.* **2021,** *143*, 5951-5957.

53. Wu, W.; Chen, K.; Yu, H.; Zhu, J.; Feng, Y.; Wang, J.; Huang, X.; Li, L.; Hao, H.; Wang, T.; Wang, N.; Naumov, P., Trimodal operation of a robust smart organic crystal. *Chem. Sci.* **2024,** *15*, 9287-9297.

54. Nag, S.; Bhowmik, A.; Mishra, M. K.; Ghosh, S., Integrating Thermal Actuation and Acidochromism in Schiff Base Molecular Crystals. *Cryst. Growth Des.* **2025,** *25*, 10527-10540.

55. Ranjan, S.; Honda, H.; Takamizawa, S., Thermo-mechanical reversibility in a shape memory organic salt. *J. Mater. Chem. C* **2022,** *10*, 12765-12775.

56. Catalano, L.; Sharma, R.; Karothu, D. P.; Saccone, M.; Elishav, O.; Chen, C.; Juneja, N.; Volpi, M.; Jouclas, R.; Chen, H. Y.; Liu, J.; Liu, G.; Gopi, E.; Ruzie, C.; Klimis, N.; Kennedy, A. R.; Vanderlick, T. K.; McCulloch, I.; Ruggiero, M. T.; Naumov, P.; Schweicher, G.; Yaffe, O.; Geerts, Y. H., Toward On-Demand Polymorphic Transitions of Organic Crystals via Side Chain and Lattice Dynamics Engineering. *J. Am. Chem. Soc.* **2024,** *146*, 31911-31919.

57. Wang, Z. H.; Shi, R. C.; Tahir, I.; Karothu, D. P.; Cheng, P. X.; Han, W. Q.; Li, L.; Zheng, Y. S.; Naumov, P.; Xu, J. L.; Bu, X. H., Thiophene Sulfone Single Crystal as a Reversible Thermoelastic Linear Actuator with an Extended Stroke and Second-Harmonic Generation Switching. *J. Am. Chem. Soc.* **2025,** *147*, 7749-7756.

58. Centore, R.; Causà, M., Translating microscopic molecular motion into macroscopic body motion: Reversible self-reshaping in the solid state transition of an organic crystal. *Cryst. Growth Des.* **2018,** *18*, 3535-3543.
