# Supplementary material for: Large Thermo- and Mechanosalient Actuation via Cooperative Twist Elasticity-Induced Packing Motif Conversion
Source: J Am Chem Soc. 2026 Jul 1;148(27):28602–15. doi: 10.1021/jacs.6c05413 (PMC13383629; doi:10.1021/jacs.6c05413)

checkCIF/PLATON report

You have not supplied any structure factors. As a result the full set of tests cannot be run.

THIS REPORT IS FOR GUIDANCE ONLY. IF USED AS PART OF A REVIEW PROCEDURE FOR PUBLICATION, IT SHOULD NOT REPLACE THE EXPERTISE OF AN EXPERIENCED CRYSTALLOGRAPHIC REFEREE.

No syntax errors found. CIF dictionary Interpreting this report

Datablock: B5_R

```
Bond precision:   C-C = 0.0126 A          Wavelength=0.70000

Cell:              a=43.062(9)      b=7.1710(14)       c=6.7310(14)
                   alpha=90         beta=90.46(3)      gamma=90
Temperature:       298 K
```

	Calculated	Reported
Volume	2078.5(7)	2078.4(7)
Space group	P 21/c	P 21/c
Hall group	-P 2ybc	-P 2ybc
Moiety formula	C48 H64 N2 O2	C48 H64 N2 O2
Sum formula	C48 H64 N2 O2	C48 H64 N2 O2
Mr	701.01	701.01
Dx,g cm-3	1.120	1.120
Z	2	2
Mu (mm-1)	0.064	0.064
F000	764.0	764.0
F000'	764.25	
h,k,lmax	51,8,8	51,8,8
Nref	3824	3454
Tmin,Tmax	0.999,1.000	0.895,1.000
Tmin'	0.999	

```
Correction method= # Reported T Limits: Tmin=0.895 Tmax=1.000
AbsCorr = EMPIRICAL

Data completeness= 0.903           Theta(max)= 24.993

R(reflections)= 0.1851( 1942)                     wR2(reflections)=
                                                  0.5275( 3454)
S = 1.878                   Npar= 236
```

The following ALERTS were generated. Each ALERT has the format
test-name_ALERT_alert-type_alert-level.
Click on the hyperlinks for more details of the test.

Alert level A

PLAT029_ALERT_3_A _diffrn_measured_fraction_theta_full value Low . 0.904 Why?

Author Response: Since the Pohang Accelerator Laboratory 2D Beamline goniostat has only one omega-axis, diffrn_measured_fraction_theta_full is not fully covered as 0.904, especially for the low symmetry such as the monoclinic system. As this is an inherent problem, other command and option were not helpful to improve the completeness.

PLAT084_ALERT_3_A High wR2 Value (i.e. > 0.25) .... 0.53 Report

Author Response: Weakly diffracting crystal leading to relatively poor data of challenging sample.

Alert level B

PLAT082_ALERT_2_B High R1 Value .... 0.19 Report

Author Response: Lack of the crystal quality for this class of compounds. Weakly diffracting cause by very tiny crystal size.

PLAT220_ALERT_2_B NonSolvent Resd 1 C Ueq(max)/Ueq(min) Range 6.2 Ratio

Author Response: Unresolvable disorder due to limited resolution of crystals data.

PLAT242_ALERT_2_B Low 'MainMol' Ueq as Compared to Neighbors of C11 Check

Author Response: The atom is located in a position were either thermal motion or multip positions are possible. Modeling of the disorder was attempted and unsuccessful. The enlarged displacement parameters are indicative of the probable thermal moti which result in this alert.

PLAT340_ALERT_3_B Low Bond Precision on C-C Bonds .... 0.01262 Ang.

Author Response: Limited resolution and relatively imprecise diffraction data from challenging crystal.

Alert level C

```
DIFMX02_ALERT_1_C  The maximum difference density is > 0.1*ZMAX*0.75
            The relevant atom site should be identified.
PLAT097_ALERT_2_C Large Reported Max.  (Positive) Residual Density       0.61 eA-3
PLAT222_ALERT_3_C NonSolvent Resd 1  H   Uiso(max)/Uiso(min) Range        7.0 Ratio
PLAT234_ALERT_4_C Large Hirshfeld Difference C6       --C7       .       0.17 Ang.
PLAT234_ALERT_4_C Large Hirshfeld Difference C17      --C18      .       0.16 Ang.
PLAT242_ALERT_2_C Low    'MainMol' Ueq as Compared to Neighbors of         C9 Check
```

Author Response: The atom is located in a position were either thermal motion or multip positions are possible. Modeling of the disorder was attempted and unsuccessful. The enlarged displacement parameters are indicative of the probable thermal moti which result in this alert.

```
PLAT250_ALERT_2_C Large U3/U1 Ratio for <U(i,j)> Tensor(Resd    1)        2.2 Note
PLAT260_ALERT_2_C Large Average Ueq of Residue Including       O1      0.128 Check
```

Alert level G

```
ABSMU01_ALERT_1_G  Calculation of _exptl_absorpt_correction_mu
                not performed for this radiation type.
PLAT802_ALERT_4_G CIF Input Record(s) with more than 80 Characters          2 Info
PLAT804_ALERT_5_G Number of ARU-Code Packing Problem(s) in PLATON           1 Info
PLAT883_ALERT_1_G No Info/Value for _atom_sites_solution_primary .     Please Do !
PLAT933_ALERT_2_G Number of HKL-OMIT Records in Embedded .res File          1 Note
               -5  1  1,
PLAT941_ALERT_3_G Average HKL Measurement Multiplicity ..........         3.5 Low
PLAT967_ALERT_5_G Note: Two-Theta Cutoff Value in Embedded .res ..       50.0 Degree
```

```
   2 ALERT level A = Most likely a serious problem - resolve or explain
   4 ALERT level B = A potentially serious problem, consider carefully
   8 ALERT level C = Check. Ensure it is not caused by an omission or oversight
   7 ALERT level G = General information/check it is not something unexpected

   3 ALERT type 1 CIF construction/syntax error, inconsistent or missing data
   8 ALERT type 2 Indicator that the structure model may be wrong or deficient
   5 ALERT type 3 Indicator that the structure quality may be low
   3 ALERT type 4 Improvement, methodology, query or suggestion
   2 ALERT type 5 Informative message, check
```

It is advisable to attempt to resolve as many as possible of the alerts in all categories. Often the minor alerts point to easily fixed oversights, errors and omissions in your CIF or refinement strategy, so attention to these fine details can be worthwhile. In order to resolve some of the more serious problems it may be necessary to carry out additional measurements or structure refinements. However, the purpose of your study may justify the reported deviations and the more serious of these should normally be commented upon in the discussion or experimental section of a paper or in the "special_details" fields of the CIF. checkCIF was carefully designed to identify outliers and unusual parameters, but every test has its limitations and alerts that are not important in a particular case may appear. Conversely, the absence of alerts does not guarantee there are no aspects of the results needing attention. It is up to the individual to critically assess their own results and, if necessary, seek expert advice.

Publication of your CIF in IUCr journals

A basic structural check has been run on your CIF. These basic checks will be run on all CIFs submitted for publication in IUCr journals (*Acta Crystallographica*, *Journal of Applied Crystallography*, *Journal of Synchrotron Radiation*); however, if you intend to submit to *Acta Crystallographica Section C* or *E* or *IUCrData*, you should make sure that full publication checks are run on the final version of your CIF prior to submission.

Publication of your CIF in other journals

Please refer to the *Notes for Authors* of the relevant journal for any special instructions relating to CIF submission.

PLATON version of 22/08/2024; check.def file version of 21/08/2024

Datablock B5_R - ellipsoid plot

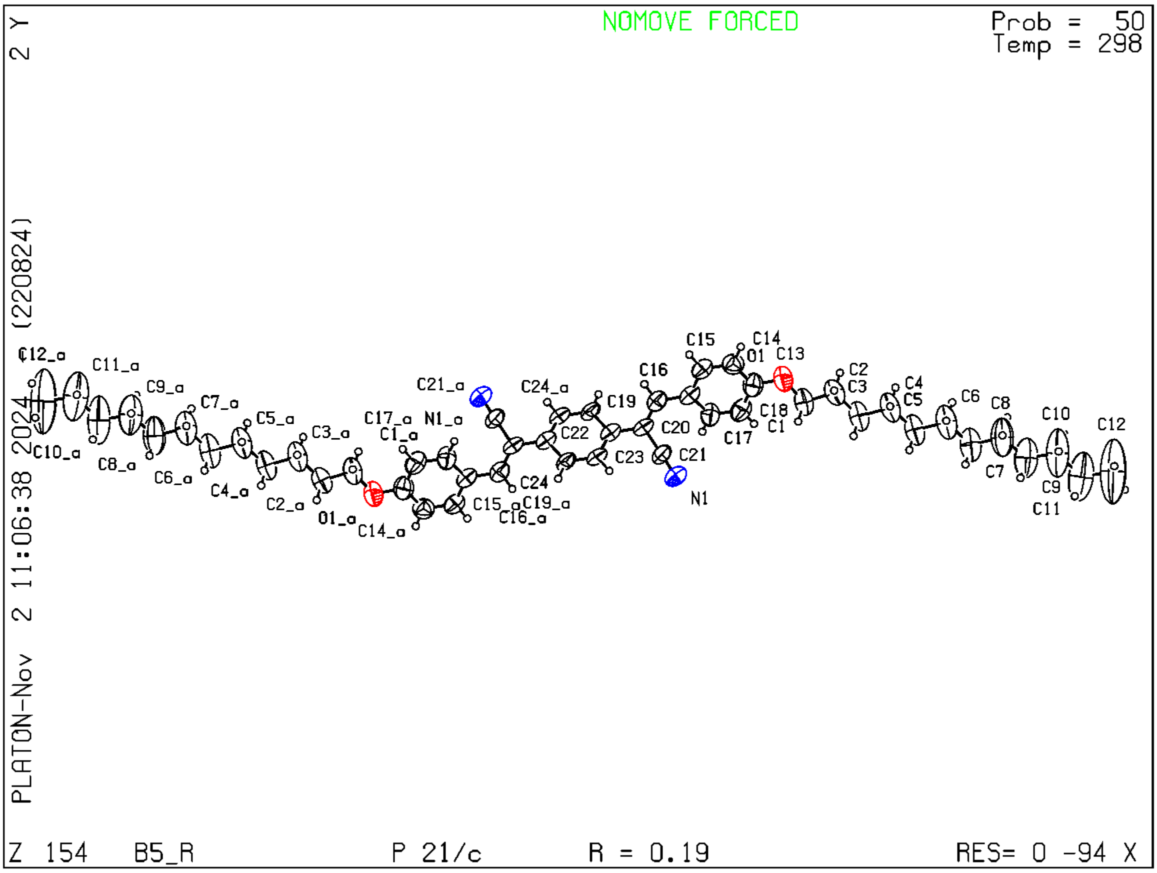

Supplement: Supplementary file 1 [file B-phase_checkcif.pdf › Cif and checkcif/B-phase_checkcif.pdf]
