# Supplementary material for: Large Thermo- and Mechanosalient Actuation via Cooperative Twist Elasticity-Induced Packing Motif Conversion
Source: J Am Chem Soc. 2026 Jul 1;148(27):28602–15. doi: 10.1021/jacs.6c05413 (PMC13383629; doi:10.1021/jacs.6c05413)

checkCIF/PLATON report

You have not supplied any structure factors. As a result the full set of tests cannot be run.

THIS REPORT IS FOR GUIDANCE ONLY. IF USED AS PART OF A REVIEW PROCEDURE FOR PUBLICATION, IT SHOULD NOT REPLACE THE EXPERTISE OF AN EXPERIENCED CRYSTALLOGRAPHIC REFEREE.

No syntax errors found.   CIF dictionary   Interpreting this report

Datablock: a12Y100

```
Bond precision:   C-C = 0.0087 A           Wavelength=0.70000

Cell:             a=5.7010(11)       b=7.4030(15)       c=48.25(1)
                  alpha=88.30(3)     beta=87.70(3)      gamma=89.92(3)
Temperature:      100 K

                  Calculated                    Reported
Volume            2033.8(7)                     2033.8(7)
Space group       P -1                          P -1
Hall group        -P 1                          -P 1
Moiety formula    C48 H64 N2 O2                 C48 H64 N2 O2
Sum formula       C48 H64 N2 O2                 C48 H64 N2 O2
Mr                701.01                        701.01
Dx,g cm-3         1.145                         1.145
Z                 2                             2
Mu (mm-1)         0.066                         0.066
F000              764.0                         764.0
F000'             764.25
h,k,lmax          7,9,62                        7,9,59
Nref              9317                          6646
Tmin,Tmax         0.999,0.999                   0.890,1.000
Tmin'             0.992

Correction method= # Reported T Limits: Tmin=0.890 Tmax=1.000
AbsCorr = EMPIRICAL

Data completeness= 0.713           Theta(max)= 26.993

                                                     wR2(reflections)=
R(reflections)= 0.0863( 1968)                        0.2611( 6646)

S = 0.899                    Npar= 471
```

The following ALERTS were generated. Each ALERT has the format
test-name_ALERT_alert-type_alert-level.
Click on the hyperlinks for more details of the test.

Alert level A

```
PLAT029_ALERT_3_A _diffrn_measured_fraction_theta_full value Low .      0.816 Why?
```

Author Response: The diffrn_measured_fraction_theta_full value is 0.816, which is less than ideal, due to instrumental limitations. The 2D beamline at the Pohang Accelerator Laboratory is equipped with a goniostat that has only a single omega axis, making it difficult to achieve full data completeness, particularly for low-symmetry systems such as triclinic crystals. Also,

Alert level B

```
PLAT026_ALERT_3_B Ratio Observed / Unique Reflections (too) Low ..        30% Check
```

Author Response: The crystal, being of limited quality and very tiny and thin, does not diffract X-rays well. Consequently, the number of observed reflections is rather low.

Alert level C

```
PLAT084_ALERT_3_C High wR2 Value (i.e. > 0.25) ...................       0.26 Report
PLAT250_ALERT_2_C Large U3/U1 Ratio for <U(i,j)> Tensor(Resd    1)        2.3 Note
PLAT340_ALERT_3_C Low Bond Precision on  C-C Bonds ...............    0.00869 Ang.
```

Alert level G

```
ABSMU01_ALERT_1_G  Calculation of _exptl_absorpt_correction_mu
                 not performed for this radiation type.
PLAT013_ALERT_1_G N.O.K.   _shelx_hkl_checksum Found in CIF ......     Please Check
PLAT154_ALERT_1_G The s.u.'s on the Cell Angles are Equal ..(Note)       0.03 Degree
PLAT180_ALERT_4_G Check Cell Rounding: # of Values Ending with 0 =          4 Note
PLAT883_ALERT_1_G Absent Datum for _atom_sites_solution_primary ..     Please Do !
PLAT933_ALERT_2_G Number of HKL-OMIT Records in Embedded .res File          9 Note
                 2 -2 34,   4  0 29,   2  6  3,  -3 -6  1,   7  1 15,   2  4  2,
                -7  2 18,   2  4  1,  -1 -2  1,
PLAT941_ALERT_3_G Average HKL Measurement Multiplicity ..........         1.9 Low
PLAT952_ALERT_5_G Calculated (ThMax) and CIF-Reported Lmax Differ.          3 Units
PLAT967_ALERT_5_G Note: Two-Theta Cutoff Value in Embedded .res ..       54.0 Degree
```

1 **ALERT level A** = Most likely a serious problem - resolve or explain
1 **ALERT level B** = A potentially serious problem, consider carefully
3 **ALERT level C** = Check. Ensure it is not caused by an omission or oversight
9 **ALERT level G** = General information/check it is not something unexpected

```
4 ALERT type 1 CIF construction/syntax error, inconsistent or missing data
2 ALERT type 2 Indicator that the structure model may be wrong or deficient
5 ALERT type 3 Indicator that the structure quality may be low
1 ALERT type 4 Improvement, methodology, query or suggestion
2 ALERT type 5 Informative message, check
```

It is advisable to attempt to resolve as many as possible of the alerts in all categories. Often the minor alerts point to easily fixed oversights, errors and omissions in your CIF or refinement strategy, so attention to these fine details can be worthwhile. In order to resolve some of the more serious problems it may be necessary to carry out additional measurements or structure refinements. However, the purpose of your study may justify the reported deviations and the more serious of these should normally be commented upon in the discussion or experimental section of a paper or in the "special_details" fields of the CIF. checkCIF was carefully designed to identify outliers and unusual parameters, but every test has its limitations and alerts that are not important in a particular case may appear. Conversely, the absence of alerts does not guarantee there are no aspects of the results needing attention. It is up to the individual to critically assess their own results and, if necessary, seek expert advice.

Publication of your CIF in IUCr journals

A basic structural check has been run on your CIF. These basic checks will be run on all CIFs submitted for publication in IUCr journals (*Acta Crystallographica*, *Journal of Applied Crystallography*, *Journal of Synchrotron Radiation*); however, if you intend to submit to *Acta Crystallographica Section C* or *E* or *IUCrData*, you should make sure that full publication checks are run on the final version of your CIF prior to submission.

Publication of your CIF in other journals

Please refer to the *Notes for Authors* of the relevant journal for any special instructions relating to CIF submission.

PLATON version of 04/06/2025; check.def file version of 30/05/2025

Datablock a12Y100 - ellipsoid plot

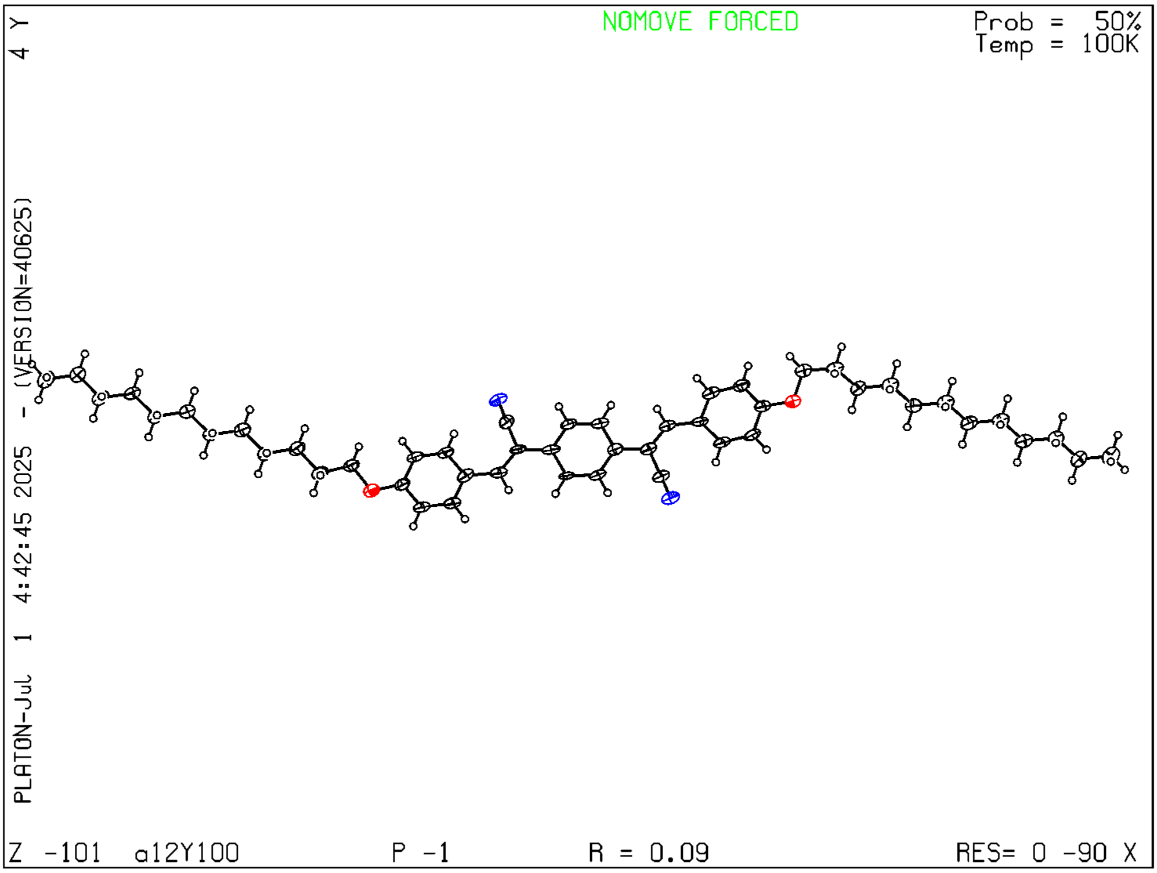

Supplement: Supplementary file 1 [file Y-phase_100K.pdf › Cif and checkcif/Y-phase_100K.pdf]
